# Squaramide-Tethered Sulfonamides and Coumarins: Synthesis, Inhibition of Tumor-Associated CAs IX and XII and Docking Simulations

**DOI:** 10.3390/ijms23147685

**Published:** 2022-07-12

**Authors:** Giulia Arrighi, Adrián Puerta, Andrea Petrini, Francisco J. Hicke, Alessio Nocentini, Miguel X. Fernandes, José M. Padrón, Claudiu T. Supuran, José G. Fernández-Bolaños, Óscar López

**Affiliations:** 1 Departamento de Química Orgánica, Facultad de Química, Universidad de Sevilla, Apartado 1203, E-41071 Seville, Spain; giulia.arrighi1@stud.unifi.it (G.A.); javipoke@hotmail.es (F.J.H.); bolanos@us.es (J.G.F.-B.); 2 BioLab, Instituto Universitario de Bio-Orgánica Antonio González (IUBO-AG), Universidad de La Laguna, c/Astrofísico Francisco Sánchez 2, E-38206 La Laguna, Spain; apuertaa@ull.es (A.P.); mfernand@ull.es (M.X.F.); jmpadron@ull.es (J.M.P.); 3 NEUROFARBA Department, Sezione di Scienze Farmaceutiche e Nutraceutiche, University of Florence, 50019 Florence, Italy; andrea.petreni@unifi.it (A.P.); alessio.nocentini@unifi.it (A.N.)

**Keywords:** carbonic anhydrases, sulfonamides, coumarins, squaramides, docking simulations

## Abstract

**(1) Background:** carbonic anhydrases (CAs) are attractive targets for the development of new anticancer therapies; in particular, CAs IX and XII isoforms are overexpressed in numerous tumors. **(2) Methods:** following the *tail approach*, we have appended a hydrophobic aromatic tail to a pharmacophore responsible for the CA inhibition (aryl sulfonamide, coumarin). As a linker, we have used squaramides, featured with strong hydrogen bond acceptor and donor capacities. **(3) Results:** Starting from easily accessible dimethyl squarate, the title compounds were successfully obtained as crystalline solids, avoiding the use of chromatographic purifications. Interesting and valuable SARs could be obtained upon modification of the length of the hydrocarbon chain, position of the sulfonamido moiety, distance of the aryl sulfonamide scaffold to the squaramide, stereoelectronic effects on the aromatic ring, as well as the number and type of substituents on C-3 and C-4 positions of the coumarin. **(4) Conclusions:** For sulfonamides, the best profile was achieved for the *m*-substituted derivative **11** (*K*_i_ = 29.4, 9.15 nM, CA IX and XII, respectively), with improved selectivity compared to acetazolamide, a standard drug. Coumarin derivatives afforded an outstanding selectivity (*K*_i_ > 10,000 nM for CA I, II); the lead compound (**16c**) was a strong CA IX and XII inhibitor (*K*_i_ = 19.2, 7.23 nM, respectively). Docking simulations revealed the key ligand-enzyme interactions.

## 1. Introduction

Squaric acid (3,4-dihydroxycyclobut-3-ene-1,2-dione), also called quadratic acid due to its shape—close to a perfect square—is a four-membered ring system that exhibits some astonishing properties, like strong acidity, and strong hydrogen bonding; the latter feature is responsible for its high melting point temperature and low water solubility [1]. Squaric acid can be easily transformed into squarate esters upon reaction with alcohols; squarates can be further transformed into mixed squaramate (reaction with one equivalent of an amine), and also into symmetrical and non-symmetrical squaramides by condensation with one (2.0 mol. equiv.) or two amines (1.0 mol. equiv. each) [1]. The most remarkable physical property of squaramides is their capacity of participating in bidirectional hydrogen bonding interactions (if prepared from primary amines), as acceptors (carbonyl groups), and as donors (NH groups) [2]. Moreover, it has been found that the intrinsic aromatic character of the cyclobut-3-ene-1,2-dione system is directly correlated with the strength of the hydrogen bonding in squaramides [2].

Squaramides are currently gaining great attention and have found a myriad of applications [3] in diverse fields like: molecular sensors [4], including ion pair receptors [5,6], metal-organic frameworks (MOFs, due to their self-assembly properties) [7], organocatalysis [8], including Henry [9], Mannich [10], and aldol [11] reactions, asymmetric fluorinations [12], and cycloadditions/cascade reactions [13] to furnish carbo- and heterocycles, and also within medicinal chemistry [14]. Interestingly, squaramides are currently considered as vinylogous amides with increased conformational restriction and have been used in the bio-isosteric replacement of frequent connectors in drug design, like (thio)ureas, guanidines, and cyanoguanidines [15]. In this context, the squaramido motif has been incorporated, for instance, into RNA polymerase [16], histone deacetylase (HDAC) [17] and cholinesterase inhibitors [18], anti-chagasic [19] and anti-tuberculosis agents [20], fluorescent ligands for cell imaging [21], some of which also providing antiproliferative properties [22], photocages for photoactivated chemotherapy (PACT) [23], or into radiopharmaceuticals that conjugate chelators of radiometals and vectors with interest in radio-imaging and chemotherapy [24].

We herein envisioned the possibility of using squaramides as an interesting tether in the design of novel carbonic anhydrase (CA, EC 4.2.1.1) inhibitors, an area that has not been exploited so far. CAs are a superfamily of ubiquitous metalloenzymes in both, prokaryotic and eukaryotic organisms (in most of the cases, with Zn^2+^ as the prosthetic group) [25] that catalyze a simple, yet essential process, that is the reversible hydration of CO_2_ to furnish HCO_3_^−^ plus a proton [26]. The spontaneous and non-catalyzed reaction is not fast enough to cover the physiological demand; high levels of CO_2_ have deleterious effects in organisms, and pH homeostasis is essential in numerous biochemical pathways and processes [27], like electrolyte secretion, lipogenesis, gluconeogenesis, or ureagenesis [28]. Remarkably, CA-mediated biocatalysis of this reaction affords a significant rate increase (*k*_cat_ 10^4^–10^6^) [29], making CAs one of the fastest enzymes identified so far.

There are currently eight gene families of CAs, identified by Greek letters (α, β, γ, δ, ζ, η, θ, ι), and distributed among all kingdoms of life [29]. Mammals only encode for α-CAs, and in the case of humans, 15 isoforms can be found in different tissues and with different functions [30]: hCAs I, II, III, VII, VIII, X, XI, XIII can be found in cytosol; hCA IV is a glycosylphosphatidylinositol (GPI)-anchored protein; hCA VA and VB are located in the mitochondrial matrix; hCA VI is secreted in saliva and milk; hCA IX, XII and XIV are transmembrane enzymes. The only isoforms devoid of a known activity are hCAs VIII, X and XI, the so-called CARPs (CA-related proteins) [31].

Due to the countless implications of CAs in biological processes, they are currently a validated drug target, with some molecules already marketed [32]; in fact, the development of activators [33,34] and inhibitors [28,35,36] of CAs is a hot topic in the medicinal chemistry area. There are several inhibition mechanisms [37], among which metal chelation (sulfonamides and their isosteric sulfamates, dithiocarbamates, hydroxamates), or entry blockade through prior CA-mediated transformations of the inhibitor (coumarins) are the most widely exploited.

CA inhibitors can be used for the development of anti-infective agents (CAs are responsible for the survival and virulence of certain pathogens) [38], for the treatment of glaucoma [39], cerebral ischemia [40], rheumatoid arthritis [41], obesity [42], neuropathic pain [43], epilepsy [44], or cancer [45]; in this context, it is important to highlight that CA IX and XII are overexpressed in numerous tumors, where they control the microenvironment pH (acidification of the extracellular medium) [46] and certain metabolic processes connected to tumor growth and metastasis [47]. Recent evidence also correlates CA inhibition with neuroprotective effects in Alzheimer’s disease [48].

## 2. Results and Discussion

### 2.1. Drug Design, Synthesis and Characterization

**Figure 1:**
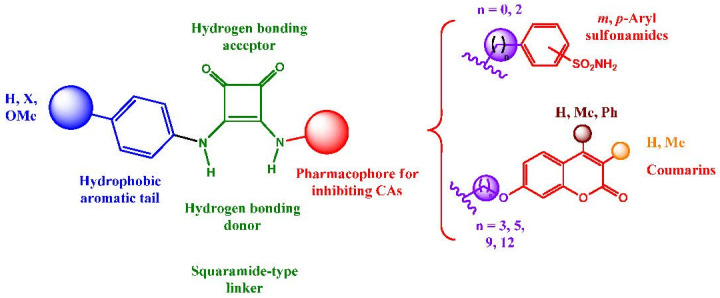
Structural design of novel squaramide-based inhibitors of CAs.

Encouraged by the remarkable properties of squaramides within the medicinal chemistry field, we decided to install it in a novel series of CA inhibitors and accomplish a comprehensive analysis of their bioactivities. Following the well-known *tail approach* [49], we envisioned the structures depicted in Figure 1. Aryl sulfonamides and coumarins were selected as the active pharmacophores for the inhibition of the metalloenzymes. Sulfonamides and isosteric sulfamates are reported to inhibit CAs by chelating the Zn^2+^ ion from the catalytic active site [50], whereas coumarins behave as *suicide inhibitors* [51]; esterase activity of CAs provokes a hydrolysis of the lactam skeleton of the coumarin to give a 2-hydroxy cinnamic acid derivative which occludes the entry to the enzyme active site [52].

In order to favor the anchorage of the inhibitor to the CA, a hydrophobic tail was incorporated for binding the hydrophobic subsite of the enzyme. Appropriate elongation might allow interaction of the ligand, not only with the catalytic site, but also with the middle and outer rims of the enzyme; modification of the electronic effects of the substituents located on the aromatic residue might also modulate the inhibitory properties. Structural modifications on the pharmacophores were also considered. And last, but not least, the squaramide tether, behaving as a vinylogous amide, or urea linkage [15] can act both, as a strong hydrogen bonding donor and acceptor and might contribute to the interaction with the hydrophilic subsite of the enzyme. Moreover, the use of a conformationally-restricted linker, as a bioisostere of more conventional linkages, can minimize the entropy loss in the formation of the enzyme-inhibitor complex, and enhance its potency/selectivity; this is a well validated approach in the drug discovery strategies that can even hinder or retard the drug degradation upon metabolization [53].

**Scheme 1:**
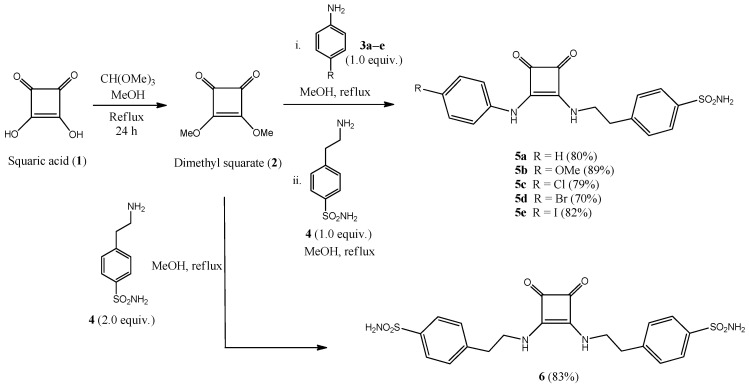
Preparation of sulfonamide-containing squaramides **5** and **6**.

The key synthetic intermediate for preparing the target compounds is 3,4-dimethoxycyclobut-3-ene-1,2-dione (dimethyl squarate, **2**), which is accessible in a multigram scale by treatment of commercially available squaric acid **1** with methanolic trimethyl orthoformate [54] under refluxing conditions (Scheme 1).

**Scheme 2:**
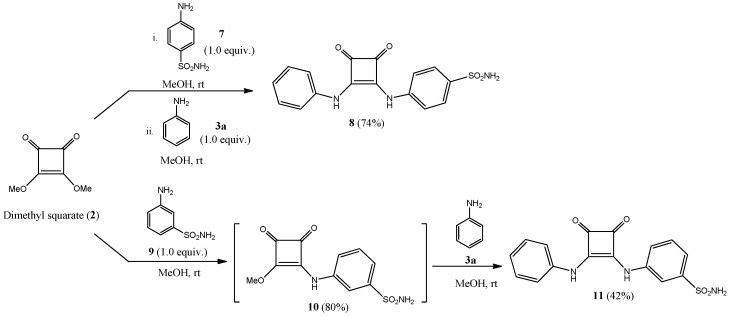
Preparation of sulfonamide-containing squaramides **8** and **11**.

Sulfonamide-containing ureido/peptide-mimetics **5**, **6**, **8** and **11** were obtained in a one-pot, two-step methodology (Scheme 1 and Scheme 2) by sequential nucleophilic displacement of the two methoxy groups with amino-derivatives **3** (anilines) and **4**, **7**, **9** (sulfonamides); final compounds were obtained as highly crystalline derivatives just by filtration, without the need of chromatographic purifications.

Reactions proceeded in higher rate and yields if the least nucleophilic amino compound was added in the first step. This is the reason for starting the synthesis with anilines **3** in Scheme 1, or with aminobenzenesulfonamides **7** and **9** in Scheme 2. 

^13^C-NMR supported the proposed structures; thus, resonances at roughly 180–184 ppm (assigned to C=O) and at 164–169 ppm (C-3 and C-4) are in agreement with reported squaramides bearing *N*,*N*′-diaryl or *N*-alkyl-*N′*-aryl substituents [55].

Squaramides **5** derived from sulfonamide **4** were obtained under refluxing conditions for both steps, in a short period of time and high yields (70–89%). Dimeric derivative **6** was obtained in a similar fashion by using 2.0 mol. equivalents of sulfonamide **4** (83% yield).

Attempts to reproduce the aforementioned conditions for *p*- and *m*-substituted aminobenzenesulfonamides **7** and **9** proved to be unsuccessful, as desired compounds were obtained in a non-resolved mixture of compounds, including symmetrical dimeric species. In order to overcome such problems, and reduce the rate of side-products, both nucleophilic displacements on dimethyl squarate **2** were attempted at rt during significantly more prolonged reaction times (5 days for **8** and 17 days for **11**).

In sulfonamides **5**, **6**, **8** and **11** several key structural motifs were modified in order to get valuable structure-activity relationships concerning their inhibitory properties against CAs: on the one hand, the stereoelectronic effects of the appended substituents on the aromatic scaffold (unsubstituted, electron-donating and electron-withdrawing, compounds **5a**–**e**); unfortunately, the *p*-fluorophenyl derivative could not be obtained in a pure form; on the other hand, the distance between the arylsulfonamido moiety and the squaramide (**5a** vs. **8**); and finally, the position of the sulfonamido motif (**8** vs. **11**).

**Scheme 3:**
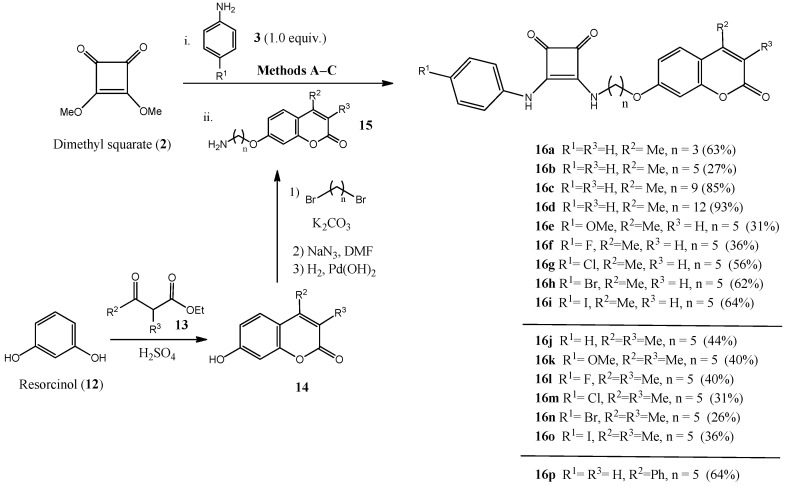
Preparation of coumarin-containing squaramides **16**.

We also envisioned the possibility of exploring a different pharmacophore for targeting CAs; we therefore replaced the arylsulfonamido motif, which very frequently leads to moderate selectivities, with coumarins (2*H*-chromen-2-ones), a natural and privileged structure within medicinal chemistry [56]. The coumarin skeleton, decorated with different substituents on C-3 and C-4 positions (compounds **14**), is easily accessible using the acid-catalyzed Pechmann condensation [57] between resorcinol **12** and the appropriate β-ketoesters **13** (Scheme 3). Williamson synthesis on the free phenolic hydroxyl group of **14** at C-7 with an excess of α,ω-dibromoalkanes, followed by nucleophilic displacement of the terminal bromine atom with NaN_3_ and Pd-catalyzed hydrogenolysis afforded amino-coumarins **15**, which were anchored on the cyclobutene-1,2-dione motif (Scheme 3) after reaction of dimethyl squarate **2** with different anilines **3** (R^1^ = H, OMe, F, Cl, Br, I). This gave access to squaramido-containing coumarins **16** in moderate to excellent yields (26–93%). Regarding the squaramide moiety, similar spectroscopic data as those obtained for arylsulfonamides were observed.

### 2.2. Biological Assessments

The vast panel of compounds reported herein, that is, sulfonamides **5**, **6**, **8** and **11**, and coumarins **16**, were tested in vitro against membrane-bound CAs IX and XII, with therapeutic interest against hypoxic tumors [58], and their activities were compared with off-target isoforms I and II (cytosolic) in order to calculate the selectivity index (S.I.). For that purpose, the stopped-flow CO_2_ hydration assay was used (Table 1). The drug acetazolamide (AAZ) was used as the reference compound. To the best of our knowledge, there is only one reported example of the use of squaramides in the inhibition of CAs; in this case, the squaramide motif was decorated with a bis-benzoxaborole fragment and turned out to be a moderate inhibitor of CAs [59].

Regarding sulfonamides, incorporation of substituents on the *p*-position of the phenyl ring (Table 1, derivatives **5b**–**e** vs. **5a**) yielded an impairment of the inhibition of off-target enzymes (*K*_i_ = 373–910 nM); nevertheless, this observation was translated into an improvement of selectivity just for *p*-bromophenyl derivative **5d**, which turned out to be the strongest inhibitors of this first series against CA IX (*K*_i_ = 67.6 nM) and XII (*K*_i_ = 85.5 nM). Insertion of electron-donating groups (OMe, **5b**) was particularly unfavorable for the inhibition of CA XII.

Dimeric sulfonamide **6**, despite keeping a good inhibitory level against tumor-associated CAs, provoked a considerable improvement in the inhibition of the off-target enzymes, and thus, a sharp decrease in the selectivity (Table 1).

The shortening of the distance between the aryl sulfonamide and the squaramide moieties (**8** vs. **5a**) led to an increased inhibitory activity against CAs I and II, but also for CA IX and XII, affording a clear selectivity improvement, particularly for CA XII, reaching the low nanomolar range (*K*_i_ = 6.57 nM). Thus, S.I. (I/XII) were found to be 2.2 and 15 for compounds **5a** and **8**, respectively, and S.I. (II/XII), 7.4 and 10 for the same two compounds (Table 1).

Further SAR analysis revealed that *m*-regioisomers **10** (intermediate compound), and **11** kept within the low nM inhibition range for CA XII. Interestingly, the latter compound can be considered the lead compound of the sulfonamide series, as a clear impairment of activity against CA I and II was observed, improving the selectivity indexes of the reference drug AAZ (Table 1).

Table 2 depicts the same kind of data for squaramide-containing coumarins **16a**–**p**; the first relevant conclusion that can be reached is that outstanding selectivities are observed for all compounds of this second series, as no relevant activities were detected for concentrations as high as 10 µM against CA I and II. This observation constitutes a significant difference with related sulfonamido counterparts.

Compounds **16a**–**d**, with an unsubstituted phenyl residue, provided information about the influence of the length of the hydrocarbon chain connecting the coumarin and squaramide moieties. Clearly, elongation led to an improvement of activity against tumor associated CAs, reaching the highest activities for *n* = 12 (**16d**) and 9 (**16c**) for CA IX and XII, respectively. Low nanomolar activities were achieved (*K*_i_ = 19.2, 18.1 nM for CA IX; 7.23, 7.91 nM for CA XII).

The second sub-series of compounds is comprised of derivatives **16e**–**i**, where the hydrocarbon chain was kept unchanged (*n* = 5), and the stereoelectronic effects (electron withdrawing and donating effects) on the *p*-position of the phenyl ring were modified, and the effects, compared with the unsubstituted counterpart **16b**. Data shown in Table 2 indicate that such substituents do not exert much influence on the bioactivities; just a moderate impairment of activity was observed for **16e** (R = OMe), **16g** (R = Cl), **16i** (R = I).

The third set of compounds includes coumarins **16j**–**o**, which were designed for analyzing the influence of a disubstituted pattern on C-3 and C-4 positions of the coumarin on the inhibition profile. The same hydrocarbon chain length (*n* = 5) and substitution pattern on the phenyl ring as previously indicated was settled. Comparison with unsubstituted **16b** revealed (Table 2) an impairment of activity for CA IX and XII in all the members of the series, CA IX being more affected. Therefore, an increase in the steric hindrance was found to be detrimental on the activity.

The same conclusion can be reached by replacing the Me group on **16b** with a Ph residue (**16p**); although being monosubstituted just on the C-4 position, such group is endowed with a high steric demand, that furnished a roughly 6.1-fold decreased activity for both enzymes when compared with **16b**.

Some representative compounds (**5a**–**e**, **16b**, **16e**–**i**) were also tested as potential antiproliferative agents against a panel of six human tumor cell lines: A549 (non-small cell lung), HBL-100 (breast), HeLa (cervix), SW1573 (non-small cell lung), T-47D (breast), WiDr (colon). Unfortunately, no relevant activity was found for the tested compounds. Derivatives **16g**–**i** were not soluble under the assay conditions; compounds **5a**–**e**, **6**, **16e** and **16f** had GI_50_ > 100 µM for all the cell lines. Compound **16b** had GI_50_ = 94 ± 10 µM for line A549, and >100 µM for the rest.

Previous data were generated under normoxic conditions, when small concentrations of CAs are present; however, under hypoxic conditions, CA IX/XII are overexpressed and constitute a survival mechanism of the tumor cells. There are extensive data in literature indicating that under hypoxic conditions, the inhibition of such enzymes constitutes a potent antitumor mechanism [60,61,62,63,64].

Moreover, it is important to mention that a series of inhibitors of CA XII have also been found to inhibit the drug efflux transporter P-glycoprotein (P-gp) [65], one of the most common mechanisms for elimination of xenobiotics, and thus, responsible for the development of chemoresistance [66], one of the major challenges to be overcome in the development of new anticancer therapies.

### 2.3. Docking Simulations

The lead compounds in each series (*m*-benzenesulfonamide **11** and coumarin **16c**) were subjected to docking simulations in order to predict the key interactions involved in their inhibition of CA XII, what can be of interest for future drug design. Squaramate **10**, although not exhibiting an optimal selectivity profile, was also included in the calculations, as it was endowed with the strongest inhibition of CA XII within both sub-series. Sulfonamides are reported [37] to interact with CAs through its deprotonated form, and thus, as anionic species; accordingly, such observations were taken into consideration for the in silico study.

**Figure 2:**
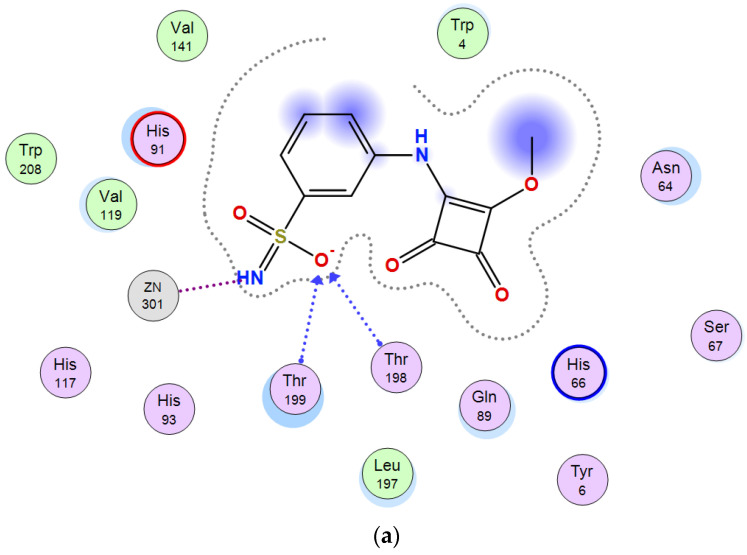

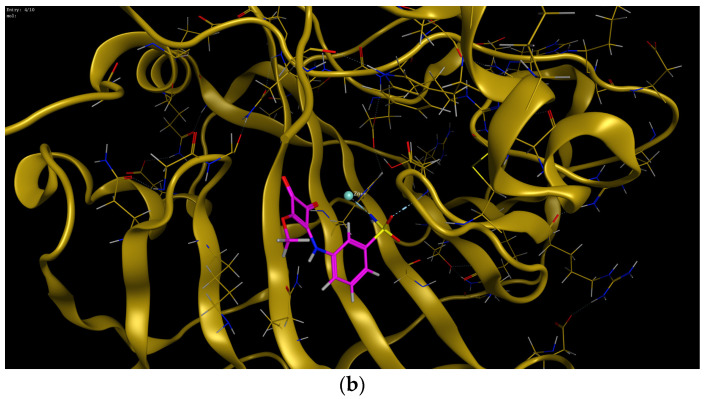
Predicted binding mode of **10** and CA XII. (**a**) Two-dimensional view of main residues involved in the ligand-protein interactions. (**b**) Three-dimensional structure of CA XII showing the binding site.

Figure 2 shows the predicted interactions of one of the most favorable poses for compound **10** binding the active site of CA XII; in this pose, the deprotonated sulfonamido motif exhibits interactions with the Zn^2+^ cation through the NH residue. Hydrogen bonding interactions between one of the oxygen atoms of the sulfonamido moiety and Thr 198, Thr 199 residues are also observed. This situation is similar to the interactions shown in crystal structures of arylsulfonamides-CA (II, IX) complexes [67]. Some other favourable poses also predict interaction, together with NH, of the SO_2_ residue and the Zn(II), due to the existence of a partial negative charge on the oxygen atoms.

**Figure 3:**
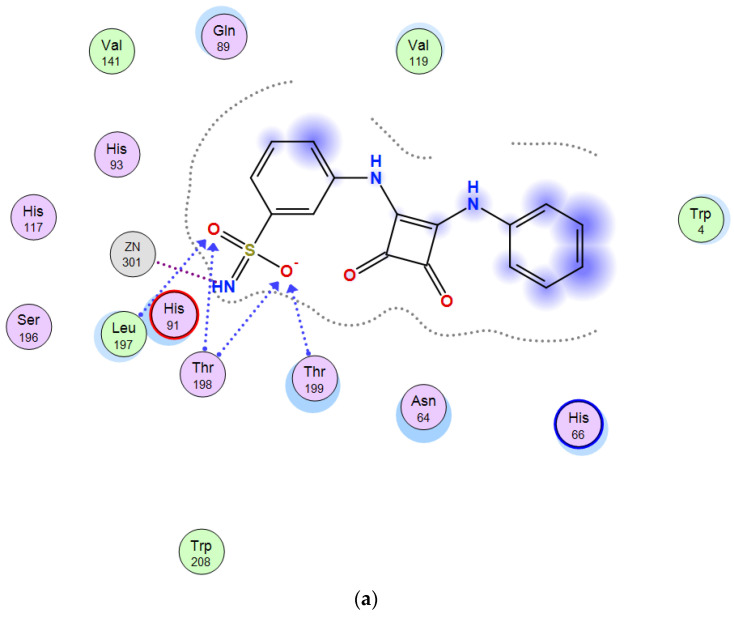

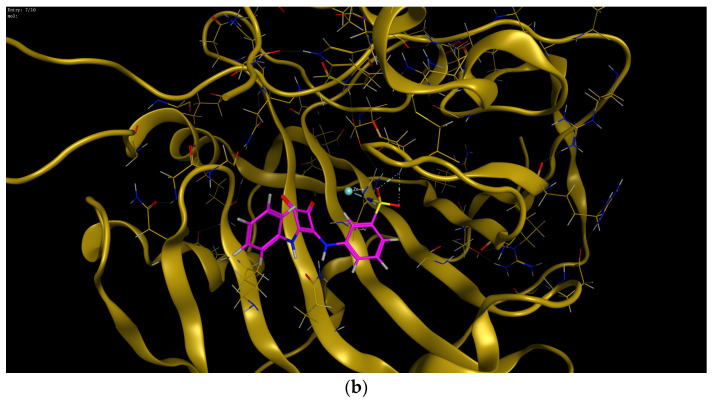
Predicted binding mode of **11** and CA XII. (**a**) Two-dimensional view of main residues involved in the ligand-protein interactions. (**b**) Three-dimensional structure of CA XII showing the binding site.

When shifting to structurally related *m*-squaramide **11**, the larger size of the appended phenyl residue compared to the methoxy group of **10**, provokes its orientation towards the exit of the cavity. Similarly, to **10**, interactions of the deprotonated sulfonamido group with Zn^2+^ (NH) and Thr 198, Thr 199 Leu 197 (oxygen atoms of the sulfonamido moiety) were observed in the most favorable poses (Figure 3).

**Scheme 4:**
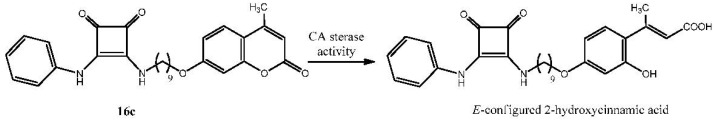
Mode of inhibition of squaramide **16c**.

As previously indicated, coumarins behave as prodrugs, as the lactone functionality is hydrolyzed by CAs to furnish the corresponding 2-hydroxycinnamic acid derivative [52], presumably with *E*-configuration (Scheme 4). This inhibitory mechanism was previously demonstrated both by X-ray crystallography and mass spectrometry [68].

In fact, the binding energy of the non-hydrolyzed form shows a remarkable impairment compared to the cinnamic acid derivative binding the enzyme in the active site (Table 3).

**Figure 4:**
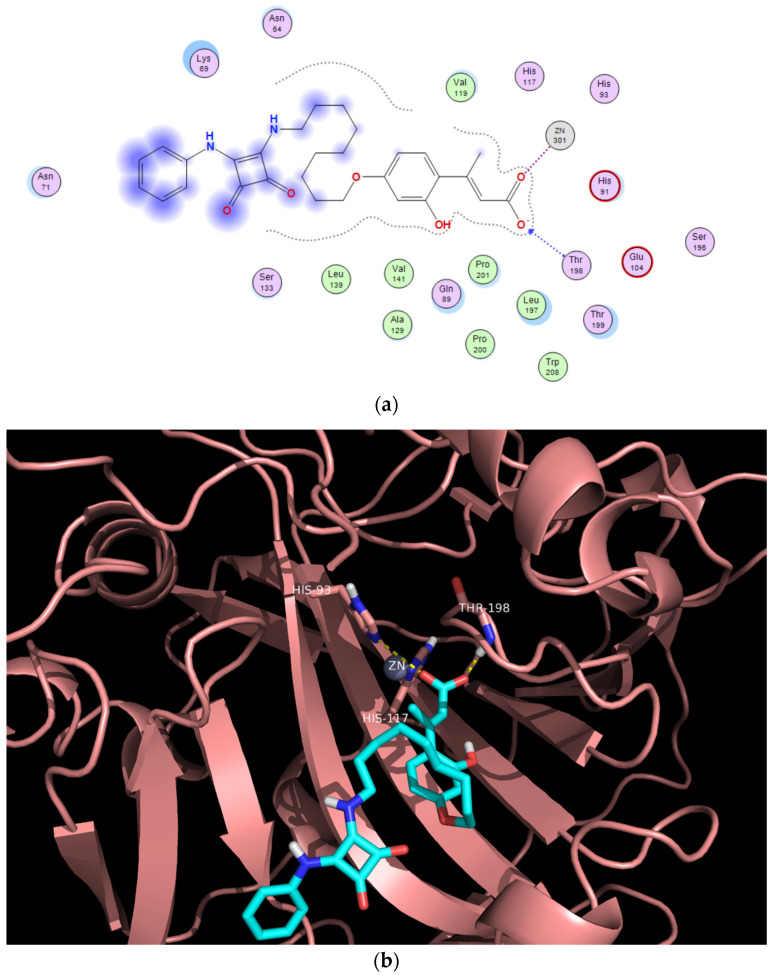
Predicted binding mode of the open form of **16c** and CA XII within the active site. (**a**) Two-dimensional view of main residues involved in the ligand-protein interactions. (**b**) Three-dimensional structure of CA XII showing the binding site.

**Figure 5:**
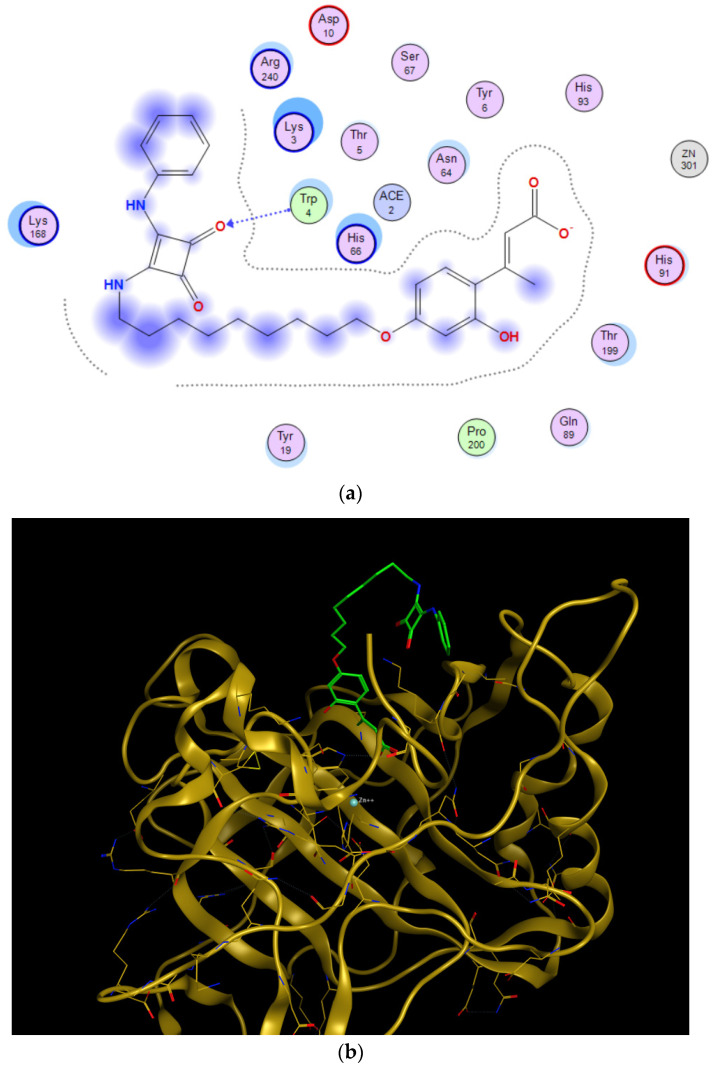
Predicted binding mode of the open form of **16c** and CA XII outside the active site. (**a**) Two-dimensional view of main residues involved in the ligand-protein interactions. (**b**) Three-dimensional structure of CA XII showing the binding outside the active site.

In this context, we have accomplished two different docking simulations for derivative **16c**, one on the cavity of the enzyme active site (Figure 4), and another one in the entry to the cavity (Figure 5), in order to predict the most favorable binding region with the corresponding *E*-configured 2-hydroxycinnamic acid derivative.

Regarding the simulation within the active site, it is the concomitant carboxylate moiety that interacts with the Zn^2+^ cation, whereas the bulky nonyl and squaramido moieties occlude the entry to the cavity of the catalytic site (Figure 4).

Nevertheless, simulations conducted at the entry of the cavity (Figure 5), where the carboxylate moiety cannot interact with the Zn(II) cation, led to a significant impairment in the binding energy (Table 3, roughly −10 kcal/mol vs. −6.9 kcal/mol). This would predict that the hydrolyzed coumarin system has the appropriate shape and size to be accommodated within the enzyme active site and interact with the Zn(II) through ionic interactions. A similar situation was recently reported for psoralen derivatives [69].

### 2.4. Prediction of Physicochemical Properties

A series of relevant molecular physicochemical properties, like logP, logS (Ali) [70], H-bond acceptors/donors, TPSA (SwissADME freeware suite) [71,72], or pKa (MolGpka) [73,74] were calculated for squaramides in order to predict their drug likeness properties (Table 4).

As it can be observed from Table 4, all compounds have the required lipophilicity, polar surface, number of H-bond acceptors/donors and molecular size so as to fulfill the Lipinsky rule of five, and therefore, to be considered to have drug likeness properties. Only derivatives **16d,h,I,n,o** slightly exceeded the limitation of 500 g/mol for their molecular weights. Sulfonamido-containing **5**, **6**, **8**, **10**, **11** are expected to be either moderately soluble, or soluble in water; coumarin derivatives **16a,b** are predicted to be moderately soluble in water, while the rest compounds of the series, with longer tethers, a disubstituted pattern on C-3/C-4 positions of the coumarin, or bearing a Ph ring at C-4 position, are expected to have poor water solubility.

With the exceptions of dimeric sulfonamide **6** and coumarin derivative **16d**, the rest of the compounds of the series are predicted to have high gastrointestinal absorption, in contrast with the reference drug AAZ. This feature might ensure an appropriate bioavailability of the title compounds.

## 3. Materials and Methods

### 3.1. Chemistry

#### 3.1.1. General Methods

TLCs (Merck 60 F_254_, gel thickness 0.25 mm) were performed using aluminum-coated sheets, using the appropriate eluant. Spots were visualized by UV light (λ = 254 nm), and by charring with 10% ethanolic vanillin containing 1% H_2_SO_4_, or with 3% ninhydrin in EtOH. Column chromatography purifications were performed using silica gel stationary phase (Merck 60, particle size 40–63 µm), eluting by gravity, or with a mild pressure, using the eluant indicated in the experimental section.

NMR spectra were registered in the Centro de Investigación, Tecnología e Innovación de la Universidad de Sevilla (CITIUS), using Bruker Avance III 300 and 500 spectrometers (300 and 500 MHz for ^1^H, 75.5 and 125.7 MHz for ^13^C), and DMSO-*d*_6_ as solvent (see Supplementary Material). Chemical shifts (δ) are expressed in ppm, and coupling constants (*J*), in Hz. Residual signals from the solvent are used as internal references [75]. Mass spectra were registered using a Q Exactive spectrometer, using Electrospray Ionization (ESI).

#### 3.1.2. General Procedure for the Preparation of Squaramides **5a**–**e**

A mixture of dimethyl squarate **2** (1.0 equiv.) and the corresponding aniline derivative **3** (1.0 equiv.) in MeOH (5 mL) was refluxed for 2 h. Then, 4-(2′-aminoethyl)benzenesulfonamide **4** (1.0 equiv.) was added, and refluxed for further 2 h. After that, the crude reaction mixture was filtered and the solid was washed with cold MeOH.

3-Phenylamino-4-[2′-(4″-sulfonamidophenyl)ethylamino]cyclobut-3-ene-1,2-dione (**5a**). Aniline (33 µL, 0.36 mmol) and 4-(2′-aminoethyl)benzenesulfonamide (72.6 mg, 0.36 mmol) were used. Compound **5a** was obtained as a grey solid. Yield: 106.6 mg (80%). Mp > 250 °C. ^1^H-NMR (500 MHz, DMSO-*d*_6_) δ 9.69 (brs, 1H, N*H*-Ar), 7.78 (m, 2H, H-3″, H-5″), 7.70 (brs, 1H, N*H*-CH_2_), 7.47 (m, 2H, H-2″, H-6″), 7.40 (m, 2H, Ar-H), 7.36–7.25 (m, 2H, SONH_2_), 7.33 (m, 2H, Ar-H), 7.02 (tt, 1H, *J*_Ar-3,4_ = *J*_Ar-4,5_ = 7.5 Hz, *J*_Ar-2,4_ = *J*_Ar-4,6_ = 1.2 Hz, Ar-H4), 3.90 (brt, *J*_H,H_ = 6.6 Hz, CH_2_N), 2.99 (t, 1H, C*H*_2_-Ar) ppm; ^13^C-NMR (125.7 MHz, DMSO-*d*_6_) δ 184.0, 180.4 (C=O), 169.1, 163.7 (C-3, C-4), 142.7, 142.4 (C-1″, C-4″), 139.0 (Ar-C*ipso*), 129.3 (Ar-C), 125.8 (Ar-C), 122.7 (Ar-C), 118.1 (Ar-C*p*), 44.6 (CH_2_-N), 36.6 (CH_2_-Ar) ppm; HRESI-MS *m*/*z* calcd. for C_18_H_17_N_3_NaO_4_S ([M + Na]^+^): 394.0832, found: 394.0829.

3-(*p*-Methoxyphenyl)amino-4-[2′-(4″-sulfonamidophenyl)ethylamino]cyclobut-3-ene-1,2-dione (**5b**). *p*-Methoxyaniline (45.6 mg, 0.36 mmol) and 4-(2′-aminoethyl)benzenesulfonamide (72.6 mg, 0.36 mmol) were used. Compound **5b** was obtained as a grey solid. Yield: 128.7 mg (89%). Mp > 250 °C. ^1^H-NMR (500 MHz, DMSO-*d*_6_) δ 9.57 (brs, 1H, N*H*-Ar), 7.77 (m, 2H, H-3″, H-5″), 7.55 (brs, 1H, N*H*-CH_2_), 7.46 (m, 2H, H-2″, H-6″), 7.30 (m, 4H, 2Ar-H, NH_2_), 6.90 (m, 2H, 2Ar-H), 3.87 (brq, *J*_H,H_ = *J*_NH,CH3_ = 6.7 Hz, C*H*_2_-NH), 2.98 (t, 2H, CH_2_-Ar) ppm; ^13^C-NMR (125.7 MHz, DMSO- *d*_6_) δ 183.5, 180.6 (C=O), 168.6, 163.8 (C-3, C-4), 155.3 (Ar-*C*-OMe), 142.7, 142.3 (C-1″, C-4″), 132.1 (Ar-C*ipso*), 129.3, 125.8 (C-2″, C-3″, C-5″, C-6″), 119.7 (Ar-C*o*), 114.5 (Ar-C*m*), 55.3 (OMe), 44.5 (CH_2_-N), 36.6 (C*H*_2_-Ar) ppm; HRESI-MS *m*/*z* calcd. for C_19_H_19_N_3_NaO_5_S ([M + Na]^+^): 424.0938, found: 424.0931.

3-(*p*-Chlorophenyl)amino-4-[2′-(4″-sulfonamidophenyl)ethylamino]cyclobut-3-ene-1,2-dione (**5c**). *p*-Chloroaniline (46.7 mg, 0.36 mmol) and 4-(2′-aminoethyl)benzenesulfonamide (72.6 mg, 0.36 mmol) were used. Compound **5c** was obtained as a white solid. Yield: 115.6 mg (79%). Mp > 250 °C. ^1^H-NMR (500 MHz, DMSO-*d*_6_) δ 9.72 (brs, 1H, N*H*-Ar), 7.78 (m, 2H, H-3″, H-5″), 7.62 (brs, 1H, N*H*-CH_2_), 7.46 (m, 2H, H-2″, H-6″), 7.40–7.35 (m, 4H, 2Ar-H, NH2), 7.30 (m, 2H, 2Ar-H), 3.87 (brq, *J*_H,H_ = *J*_NH,CH3_ = 6.5 Hz, C*H*_2_-NH), 2.97 (t, 2H, CH_2_-Ar) ppm; ^13^C-NMR (125.7 MHz, DMSO-*d*_6_) δ 184.1, 180.3 (C=O), 169.1, 163.2 (C-3, C-4), 142.5, 142.3 (C-1″, C-4″), 137.9 (Ar-C*ipso*), 129.3, 129.1, 126.5, 125.7 (Ar-C), 44.5 (CH_2_-N), 36.6 (CH_2_-Ar) ppm; HRESI-MS *m*/*z* calcd. for C_18_H_16_ClN_3_NaO_4_S ([M + Na]^+^): 428.0442, found: 428.0435.

3-(*p*-Bromophenyl)amino-4-[2′-(4″-sulfonamidophenyl)ethylamino]cyclobut-3-ene-1,2-dione (**5d**). *p*-bromoaniline (61.8 mg, 0.36 mmol) and 4-(2′-aminoethyl)benzenesulfonamide (72.6 mg, 0.36 mmol) were used. Compound **5d** was obtained as a white solid. Yield: 113.7 mg (70%). Mp > 250 °C. ^1^H-NMR (500 MHz, DMSO-*d*_6_) δ 9.73 (brs, 1H, N*H*-Ar), 7.77 (m, 2H, H-3″, H-5″), 7.64 (brs, 1H, N*H*-CH_2_), 7.49 (m, 2H, 2Ar-H), 7.46 (m, 2H, H-2″, H-6″), 7.34 (m, 2H, 2Ar-H), 7.31 (brs, 2H, CH_2_), 3.88 (brq, *J*_H,H_ = *J*_NH,CH3_ = 6.8 Hz, CH_2_-NH), 2.98 (t, 2H, C*H*_2_-Ar) ppm; ^13^C-NMR (125.7 MHz, DMSO-*d*_6_) δ 184.2, 180.4 (C=O), 169.2, 163.3 (C-3, C-4), 142.6, 142.4 (C-1″, C-4″), 138.4 (Ar-C*ipso*), 132.1, 129.3, 125.8, 120.1, 114.5 (Ar-C), 44.6 (CH_2_-N), 36.6 (CH_2_-Ar) ppm; HRESI-MS *m*/*z* calcd. for C_18_H_16_^79^BrN_3_NaO_4_S ([M + Na]^+^): 471.9937, found: 471.9932; *m*/*z* calcd. for C_18_H_16_^81^BrN_3_NaO_4_S ([M + Na]^+^): 473.9917, found: 473.9908.

3-(*p*-Iodophenyl)amino-4-[2′-(4″-sulfonamidophenyl)ethylamino]cyclobut-3-ene-1,2-dione (**5e**). *p*-iodoaniline (81.2 mg, 0.36 mmol) and 4-(2′-aminoethyl)benzenesulfonamide (72.6 mg, 0.36 mmol) were used. Compound **5e** was obtained as a yellowish solid. Yield: 147.6 mg (82%). Mp > 250 °C. ^1^H-NMR (500 MHz, DMSO-*d*_6_) δ 9.70 (brs, 1H, N*H*-Ar), 7.77 (m, 2H, H-3″, H-5″), 7.64 (m, 3H, 2Ar-H, N*H*-CH_2_), 7.46 (m, 2H, 2Ar-H), 7.30 (brs, 2H, NH_2_), 7.21 (m, 2H, 2Ar-H), 3.88 (brq, *J*_H,H_ = *J*_NH,CH3_ = 6.8 Hz, C*H*_2_-NH), 2.98 (t, 2H, CH_2_-Ar) ppm; ^13^C-NMR (125.7 MHz, DMSO-*d*_6_) δ 184.3, 180.4 (C=O), 169.2, 163.3 (C-3, C-4), 142.6, 142.4 (C-1″, C-4″), 138.8, 137.9, 129.3, 125.8 (Ar-C), 120.4 (Ar-Co), 86.1 (Ar-C-I), 44.6 (CH_2_-N), 36.6 (CH_2_-Ar) ppm; HRESI-MS *m*/*z* calcd. for C_18_H_16_IN_3_NaO_4_S ([M + Na]^+^): 519.9798, found: 519.9794.

3,4-Bis[2′-(4″-sulfonamidophenyl)ethylamino]cyclobut-3-ene-1,2-dione (**6**). A solution of dimethyl squarate **2** (54.4 mg, 0.38 mmol) and 4-(2′-aminoethyl)benzenesulfonamide **4** (154.9 mg, 0.77 mmol, 2.0 equiv.) in MeOH (15 mL) was refluxed for 2 h. Filtration afforded **6** as a white solid. Yield: 150.5 mg (83%). Mp > 250 °C. ^1^H-NMR (500 MHz, DMSO-*d*_6_) δ 7.75 (m, 4H, H-3″, H-5″), 7.42 (m, 4H, H-2″, H-6″), 7.29 (m, 4H, NH_2_), 3.75 (brs, 4H, C*H*_2_-NH), 2.91 (brt, 4H, *J*_H,H_ = 6.9 Hz, C*H*_2_-Ar) ppm; ^13^C-NMR (125.7 MHz, DMSO-*d*_6_) δ 182.6 (C=O), 167.7 (C-3, C-4), 142.8, 142.3 (C-1″, C-4″), 129.3, 125.8 (C-2″, C-3″, C-5″, C-6″), 44.2 (CH_2_-N), 36.7 (CH_2_-Ar) ppm; HRESI-MS *m*/*z* calcd. for C_20_H_22_N_4_NaO_6_S_2_ ([M + Na]^+^): 501.0873, found: 501.0868.

3-Phenylamino-4-[4′-(sulfonamidophenyl)]aminocyclobut-3-ene-1,2-dione (**8**). To a solution of dimethyl squarate **2** (83 mg, 0.58 mmol) in MeOH (7 mL) was added 4-aminobenzenosulfonamide **7** (100 mg, 0.58 mmol, 1.0 equiv.), and the corresponding mixture was stirred at rt for 5 days; formation of a precipitate was observed. Then, aniline (53 µL, 0.58 mmol, 1.0 equiv.) was added and the mixture was stirred at rt for further 48 h. The corresponding solid was filtrated and washed with cold MeOH to give compound **8** as a white solid. Yield: 148 mg, 74%. Mp > 250 °C. *R*_f_ 0.20 (1:1 cyclohexane-EtOAc). ^1^H-NMR (300 MHz, DMSO-*d*_6_) δ 10.15 (brs, 1H, NH), 10.02 (brs, 1H, NH), 7.80 (m, 2H, Ar-H), 7.61 (m, 2H, Ar-H), 7.50 (m, 2H, Ar-H), 7.40 (m, 2H, Ar-H), 7.29 (brs, 2H, SO_2_NH_2_), 7.11 (t, 1H, *J*_H,H_ = 7.2 Hz, H-4, Ph) ppm; ^13^C-NMR (125.7 MHz, DMSO-*d*_6_) δ 182.2, 181.5 (C=O), 166.4, 165.1 (C-3, C-4), 141.4, 138.3, 138.2, 129.4, 127.3, 123.6, 118.7, 118.2 ppm; HRESI-MS *m*/*z* calcd. for C_16_H_13_N_3_NaO_4_S ([M+Na]^+^): 366.0519, found: 366.0511.

3-Methoxy-4-[3′-(sulfonamidophenyl)]aminocyclobut-3-ene-1,2-dione (**10**) and 3-phenyl-4-[3′-(sulfonamidophenyl)]aminocyclobut-3-ene-1,2-dione (**11**). To a solution of dimethyl squarate **2** (83 mg, 0.58 mmol) in MeOH (7 mL) was added 3-aminobenzenosulfonamide **9** (100 mg, 0.58 mmol, 1.0 equiv.), and the corresponding mixture was stirred at rt for 5 days. Then, aniline (53 µL, 0.58 mmol) was added, and the mixture was stirred for 48 h. The precipitate was filtered and washed with cold MeOH to give 3-methoxy derivative **10** as a light yellow solid (131 mg, 80% yield). The solid was suspended again in MeOH (7 mL) and aniline (55 µL, 0.60 mmol) was added and the mixture was kept stirring at rt for 96 h. After that, ^1^H-NMR showed a 1:2 mixture of **10** and **11**. Aniline (12 µL, 0.13 mmol) was added and the reaction was kept stirring for a further 6 days. The precipitate was filtrated and washed with cold MeOH to give **11** as a white solid (67 mg, 42% yield). 

Data for **10**: *R*_f_ 0.2 (1:1 cyclohexane-EtOAc); ^1^H-NMR (300 MHz, DMSO-*d*_6_) δ 10.98 (brs, 1H, NH), 7.81 (m, 1H, Ar-H), 7.56 (m, 3H, Ar-H), 7.41 (brs, 2H, SONH_2_), 4.39 (brs, 3H, OMe) ppm; ^13^C-NMR (125.7 MHz, DMSO-*d*_6_) δ 187.8, 184.4 (C=O), 179.1, 169.2 (C-2, C-3), 145.1, 138.4, 130.0, 122.5, 121.0, 116.7 (Ar-C), 60.6 (OCH_3_) ppm; HRESI-MS *m*/*z* calcd. for C_11_H_10_N_2_NaO_5_S ([M + Na]^+^): 305.0203, found: 305.0197.

Data for **11**: Mp: 227–229 °C (dec.). *R*_f_ 0.2 (1:1 cyclohexane-EtOAc); ^1^H-NMR (300 MHz, DMSO-*d*_6_) δ 10.10 (brs, 1H, NH), 9.87 (brs, 1H, NH), 7.81 (m, 2H, Ar-H), 7.61–7.37 (m, 8H, Ar-H, SONH_2_), 7.11 (t, 1H, *J*_H,H_ = 7.3 Hz, H-4, Ph) ppm; ^13^C-NMR (125.7 MHz, DMSO-*d*_6_) δ 182.1, 181.5 (C=O), 166.0, 165.3 (C-3, C-4), 145.2, 139.0, 138.3, 130.3, 129.4, 123.5, 121.4, 120.1, 118.6, 115.3 (Ar-C) ppm; HRESI-MS *m*/*z* calcd. for C_16_H_13_N_3_NaO_4_S ([M + Na]^+^): 366.0519, found: 366.0514.

#### 3.1.3. General Procedures for the Preparation of Coumarin Derivatives **16a**–**p**

Method A. A solution of dimethyl squarate **2** (1.0 equiv.) and the corresponding aniline **3** (1.0 equiv.) in MeOH (5 mL) was heated in a Fisher-Porter tube at 100 °C for 2 h. Then, the corresponding amino-coumarin **15** (1.0 equiv.) was added and the mixture was heated in the Fisher-Porter tube at 100 °C for further 24 h. A precipitate was formed, which was filtered and washed with cold MeOH.

Method B. A solution of dimethyl squarate **2** (1.0 equiv.) and the corresponding aniline **3** (1.0 equiv.) in MeOH (7 mL) was refluxed for 4 h. Then, the corresponding amino-coumarin **15** (1.0 equiv.) was added and the reaction was refluxed for further 12 h. A precipitate was formed, which was filtered and washed with cold MeOH.

Method C. A solution of dimethyl squarate **2** (1.0 equiv.) and the corresponding aniline **3** (1.0 equiv.) in MeOH (7 mL) was kept at rt for 24 h. Then, the corresponding amino-coumarin **15** (1.0 equiv.) was added and the reaction was kept at the same temperature for further 24 h. A precipitate was formed, which was filtered and washed with cold MeOH.

3-{[3′-((4″-Methyl-2″-oxo-2″*H*-chromen-7″-yl)oxy)propyl]amino}-4-phenylaminocyclobut-3-ene-1,2-dione (**16a**). Method C. Dimethyl squarate (92 mg, 0.65 mmol), aniline (59 µL, 0.65 mmol), and **15** (151 mg, 0.65 mmol, *n* = 3, R^2^ = Me, R^3^ = H) were used. Yield: 166 mg (63%, white solid). Mp > 250 °C. *R*_f_ 0.40 (1:1 cyclohexane-EtOAc). ^1^H-NMR (500 MHz, DMSO-*d*_6_) δ 9.62 (brs, 1H, N*H*-Ar), 7.67 (m, 2H, H-5″, N*H*-CH_2_), 7.49–7.32 (m, 4H, Ar-H), 7.07–6.95 (m, 3H, Ar-H), 6.20 (brs, 1H, H-3″), 4.19 (m, 2H, CH_2_O), 3.78 (m, 2H, CH_2_N), 2.38 (brs, 3H, CH_3_), 2.08 (m, 2H, C*H*_2_-CH_2_-O) ppm; ^13^C-NMR (125.7 MHz, DMSO-*d*_6_) δ 183.9, 180.3 (C=O), 169.3, 163.6 (C-3, C-4), 161.5, 160.1 (C-2″, C-7″), 154.7, 153.3 (C-4″, C-8a″), 139.0, 129.3, 126.4, 122.5, 117.8, 112.4, 111.1 (Ar-C), 101.2 (C-8″), 65.5 (CH_2_O), 40.7 (CH2N), 29.9 (*C*H_2_-CH_2_-O), 18.1 (CH3) ppm; HRESI-MS *m*/*z* calcd. for C_23_H_20_N_2_NaO_5_ ([M + Na]^+^): 427.1264, found: 427.1256.

3-{[5′-((4″-Methyl-2″-oxo-2″*H*-chromen-7″-yl)oxy)pentyl]amino}-4-phenylaminocyclobut-3-ene-1,2-dione (**16b**). Method A. Dimethyl squarate (35 mg, 0.25 mmol), aniline (23 µL, 0.25 mmol), and **15** (65 mg, 0.25 mmol, *n* = 5, R^2^ = Me, R^3^ = H) were used. Yield: 29 mg (27%, light yellow solid). Mp > 250 °C. *R*_f_ 0.20 (1:1 cyclohexane-EtOAc). ^1^H-NMR (300 MHz, DMSO-*d*_6_) δ 9.59 (s, 1H, N*H*-Ar), 7.66–7.64 (m, 1H, NH), 7.65 (m, 2H, H-5″, N*H*-CH_2_), 7.44-7.30 (m, 4H, Ar-H), 7.04–6.93 (m, 3H, Ar-H), 6.19 (brs, 1H, H-3″), 4.10 (brt, 2H, *J*_H,H_ = 6.1 Hz, OCH_2_), 3.64 (brq, 2H, *J*_H,H_ = *J*_CH2,NH_ = 6.8 Hz, NCH_2_), 2.38 (brs, 3H, CH_3_), 1.79 (brquint, 2H, *J*_H,H_ = 7.0 Hz, CH2), 1.65 (quint, 2H, *J*_H,H_ = 6.8 Hz, CH2), 1.50 (quint, 2H, *J*_H,H_ = 6.2 Hz, CH_2_) ppm; ^13^C-NMR (125.7 MHz, DMSO-*d*_6_) δ 184.0, 180.1 (C=O), 169.2, 163.6 (C-3, C-4), 161.7, 160.1 (C-2″, C-7″), 154.7, 153.4 (C-4″, C-8a″), 139.0, 129.4, 126.4, 122.5, 117.9, 113.00, 112.4, 111.0 (Ar-C), 101.0 (C-8″), 68.1 (OCH2), 43.5 (NCH_2_), 30.2 (CH_2_), 28.0 (CH2), 22.3 (CH_2_), 18.1 (CH_3_) ppm; HRESI-MS *m*/*z* calcd. for C_25_H_24_NaN_2_O_5_ ([M + Na]^+^): 455.1577, found: 455.1569.

3-{[9′-((4″-Methyl-2″-oxo-2″*H*-chromen-7″-yl)oxy)nonyl]amino}-4-phenylaminocyclobut-3-ene-1,2-dione (**16c**). Method C. Dimethyl squarate (58 mg, 0.41 mmol), aniline (37 µL, 0.41 mmol) and **15** (130 mg, 0.41 mg, *n* = 9, R^2^ = Me, R^3^ = H) were used. Yield: 170 mg (85%, white solid). Mp: 200–201 °C. *R*_f_ 0.30 (1:1 cyclohexane-EtOAc). ^1^H-NMR (300 MHz, DMSO-*d*_6_) δ 9.59 (brs, 1H, N*H*-Ar), 7.68–7.64 (m, 2H, NH, H-5″), 7.44–7.42 (m, 2H, Ar-H), 7.35–7.30 (m, 2H, Ar-H), 7.01 (t, 1H, *J*_H,H_ = 7.3 Hz, H-4, Ph), 6.95–6.92 (m, 2H, Ar-H), 6.19 (d, 1H, *J*_H,CH3_ = 1.1 Hz, H-3″), 4.06 (t, 2H, *J*_H,H_ = 6.5 Hz, OCH_2_), 3.59 (brq, 2H, *J*_H,H_ = *J*_CH2,NH_ = 6.4 Hz, NCH_2_), 2.38 (d, 3H, *J*_H,CH3_ = 1.1 Hz, CH_3_), 1.75 (quint, 2H, *J*_H,H_ = 6.9 Hz, CH_2_), 1.57 (quint, 2H, *J*_H,H_ = 6.9 Hz, CH_2_), 1.39–1.35 (m, 2H, CH_2_), 1.31 (m, 8H, 4CH_2_) ppm; ^13^C-NMR (125.7 MHz, DMSO-*d*_6_) δ 183.9, 180.0 (C=O), 169.1, 163.3 (C-3, C-4), 161.6, 160.0 (C-2″, C-7″), 154.6, 153.3, 138.9, 129.2, 126.3, 122.4, 117.8, 112.8, 112.3, 110.9 (Ar-C), 101.0 (C-8″), 68.1 (OCH_2_), 43.5 (NCH_2_), 30.4, 28.7, 28.5, 28.4, 28.3, 25.7, 25.2 (CH_2_), 17.7 (CH3) ppm; HRESI-MS calcd*m*/*z*. for C_29_H_32_N_2_NaO_5_ ([M + Na]^+^): 511.2203, found: 511.2193.

3-{[12′-((4″-Methyl-2″-oxo-2″*H*-chromen-7″-yl)oxy)dodecyl]amino}-4-phenylaminocyclobut-3-ene-1,2-dione (**16d**). Method C. Dimethyl squarate (51 mg, 0.36 mmol), aniline (33 µL, 0.36 mmol) and **15** (130 mg, 0.36 mmol, *n* = 12, R^2^ = Me, R^3^ = H) were used. Yield: 178 mg (93%, light brown solid). Mp: 178–179 °C. *R*_f_ 0.40 (1:1 cyclohexane-EtOAc). ^1^H-NMR (300 MHz, DMSO-*d*_6_) δ 9.48 (brs, 1H, N*H*-Ar), 7.71 (brs, 1H, NH), 7.66 (m, 1H, H-5″), 7.44 (m, 2H, Ar-H), 7.33 (m, 2H, Ar-H), 7.01 (t, 1H, *J*_H,H_ = 7.3 Hz, H-4, Ph), 6.94–6.92 (m, 2H, Ar-H), 6.19 (d, 1H, *J*_H,H_ = 1.0 Hz, H-3″), 4.05 (brt, 2H, *J*_H,H_ = 6.5 Hz, OCH_2_), 3.59 (brt, 2H, *J*_H,H_ = 6.4 Hz, NCH_2_), 2.39 (d, 3H, *J*_H,H_ = 0.9 Hz, CH_3_), 1.72 (quint, 2H, *J*_H,H_ = 6.6 Hz, CH2), 1.56 (quint, 2H, *J*_H,H_ = 6.3 Hz, CH_2_), 1.40 (brquint, 2H, *J*_H,H_ = 6.3 Hz, CH_2_), 1.26 (m, 14H, 7CH_2_) ppm; ^13^C-NMR (125.7 MHz, DMSO-*d*_6_) δ 184.0, 180.1 (C=O), 169.2 (C-3), 163.5 (C-4), 161.8 (C-7″), 160.1 (C-2′), 154.7 (C-9), 153.4 (C-4″), 139.1 (C-1 Ph), 129.3 (Ar-C, Ph), 126.4 (C-5″), 122.5, 117.9 (Ar-C, Ph), 113.0 (C-10), 112.4 (C-6″), 111.0 (C-3″), 101.1 (C-8″), 68.2 (OCH_2_), 43.6 (NCH_2_), 30.5 (CH_2_), 28.9 (CH_2_), 28.9 (×3) (CH_2_), 28.7 (CH_2_), 28.5 (CH_2_), 28.4 (CH_2_), 25.8 (CH_2_), 25.4 (CH_2_), 18.1 (CH_3_) ppm; HRESI-MS *m*/*z* calcd. for C_32_H_38_N_2_NaO_5_ ([M + Na]^+^): 553.2673, found: 553.2662.

3-[(4′-Methoxyphenyl)amino]-4-{[5″-(4‴-methyl-2‴-oxo-2‴*H*-chromen-7‴-yl)oxypentyl]amino}cyclobut-3-ene-1,2-dione (**16e**). Method A. Dimethyl squarate (31 mg, 0.22 mmol), 4-methoxyaniline (27 mg, 0.22 mmol) and **15** (57 mg, 0.22 mmol, *n* = 5, R^2^ = Me, R^3^ = H) were used. Yield: 32 mg (31%, white solid). Mp: 215–216 °C. *R*_f_ 0.10 (1:1 cyclohexane-EtOAc). ^1^H-NMR (300 MHz, DMSO-*d*_6_) δ ^1^H-NMR (300 MHz, DMSO-*d*_6_) δ 9.49 (s, 1H, N*H*-Ar), 7.66 (d, 1H, *J*_5‴,6‴_ = 8.4 Hz, H-5‴), 7.55 (brs, 1H, NH), 7.34 (m, 2H, Ar-H), 6.97–6.90 (m, 4H, Ar-H), 6.20 (brs, 1H, H-3‴), 4.10 (t, 2H, *J*_H,H_ = 6.2 Hz, OCH_2_), 3.73 (s, 3H, OMe), 3.63 (brq, 2H, *J*_H,H_ = 6.7 Hz, NCH_2_), 2.39 (brs, 3H, CH_3_), 1.79 (quint, 2H, *J*_H,H_ = 7.0 Hz, CH_2_), 1.65 (quint, 2H, *J*_H,H_ = 7.3 Hz, CH_2_), 1.50 (quint, 2H, *J*_H,H_ = 6.8 Hz, CH_2_) ppm; ^13^C-NMR (125.7 MHz, DMSO-*d*_6_) δ 183.4, 180.3 (C=O), 168.8, 163.6 (C-3, C-4), 161.7, 160.1 (C-2‴, C-7‴), 155.2, 154.7, 153.4, 132.2, 126.4, 119.5, 114.5, 113.0, 112.4, 111.0 (Ar-C), 101.1 (C-8‴), 68.1 (OCH_2_), 55.3 (OMe), 43.5 (NCH_2_), 30.3 (CH_2_), 28.0 (CH_2_), 22.3 (CH_2_), 18.1 (CH_3_) ppm; HRESI-MS *m*/*z* calcd. for C26H26N2NaO6 ([M + Na]^+^): 485.1683, found: 485.1685.

3-[(4′-Fluorophenyl)amino]-4-{[5″-(4‴-methyl-2‴-oxo-2‴*H*-chromen-7‴-yl)oxypentyl]amino}cyclobut-3-ene-1,2-dione (**16f**). Method A. Dimethyl squarate (50 mg, 0.35 mmol), 4-fluoroaniline (33 µL, 0.35 mmol) and **15** (92 mg, 0.35 mmol, *n* = 5, R^2^ = Me, R^3^ = H) were used. Yield: 57 mg (36%, white solid). Mp: 224–226 °C. *R*_f_ 0.10 (1:1 cyclohexane-EtOAc). ^1^H-NMR (300 MHz, DMSO-*d*_6_) δ 9.61 (s, 1H, N*H*-Ar), 7.65 (d, 1H, *J*_5‴,6‴_ = 9.0 Hz, H-5‴), 7.66–7.61 (brs, 1H, NH), 7.42 (m, 2H, Ar-H), 7.17 (m, 2H, Ar-H), 6.96-6.93 (m, 2H, Ar-H), 6.19 (brd, 1H, *J*_H,CH3_ = 0.7 Hz, H-3‴), 4.10 (t, 2H, *J*_H,H_ = 6.0 Hz, OCH_2_), 3.63 (brq, 2H, *J*_H,H_ = *J*_CH2,NH_ = 6.8 Hz, NCH_2_), 2.38 (brs, 3H, CH_3_), 1.79 (quint, 2H, *J*_H,H_ = 6.8 Hz, CH2), 1.65 (quint, 2H, *J*_H,H_ = 6.9 Hz, CH_2_), 1.49 (quint, 2H, *J*_H,H_ = 6.7 Hz, CH_2_) ppm; ^13^C-NMR (125.7 MHz, DMSO-*d*_6_) δ 183.8, 180.2 (C=O), 169.1, 163.4 (C-3, C-4), 161.7, 160.1 (C-2‴, C-7‴), 158.0 (d, ^1^*J*_C,F_ = 239.1 Hz, C-4′), 154.7, 153.4 (Ar-C), 135.5 (d, ^4^*J*_C,F_ = 2.3 Hz, C-1′), 126.4 (Ar-C), 119.7 (brd, ^3^*J*_C,F_ = 6.2 Hz, C-2′), 115.9 (d, ^2^*J*_C,F_ = 22.6 Hz, C-3′), 113.0, 112.4, 111.0 (Ar-C), 101.1 (C-8‴), 68.1 (OCH_2_), 43.6 (NCH_2_), 30.3 (CH_2_), 28.0 (CH_2_), 22.3 (CH_2_), 18.1 (CH3) ppm; HRESI-MS *m*/*z* calcd. for C_25_H_23_FN_2_NaO_5_ ([M + Na]^+^): 473.1483, found: 473.1468.

3-[(4′-Chlorophenyl)amino]-4-{[5″-(4‴-methyl-2‴-oxo-2‴*H*-chromen-7‴-yl)oxypentyl]amino}cyclobut-3-ene-1,2-dione (**16g**). Method A. Dimethyl squarate (50 mg, 0.35 mmol), 4-chloroaniline (45 mg, 0.35 mmol) and **15** (92 mg, 0.35 mmol, *n* = 5, R^2^ = Me, R^3^ = H) were used. Yield: 92 mg (56%, light yellow solid). Mp: 225–227 °C. *R*_f_ 0.10 (1:1 cyclohexane-EtOAc). ^1^H-NMR (300 MHz, DMSO-*d*_6_) δ 9.68 (s, 1H, N*H*-Ar), 7.65 (d, 1H, *J*_5‴,6‴_ = 8.8 Hz, H-5‴), 7.67–7.62 (brs, 1H, NH), 7.45-7.36 (m, 4H, Ar-H), 6.96-6.93 (m, 2H, Ar-H), 6.19 (brd, 1H, *J*_H,CH3_ = 1.0 Hz, H-3‴), 4.10 (t, 2H, *J*_H,H_ = 6.3 Hz, OCH_2_), 3.64 (q, 2H, *J*_H,H_ = 6.3 Hz, NCH_2_), 2.38 (brd, 3H, *J*_H,H_ = 0.8 Hz CH_3_), 1.79 (quint, 2H, *J*_H,H_ = 7.1 Hz, CH_2_), 1.66 (quint, 2H, *J*_H,H_ = 7.2 Hz, CH_2_), 1.51 (m, 2H, CH_2_) ppm; ^13^C-NMR (125.7 MHz, DMSO-*d*_6_) δ 184.1, 180.1 (C=O), 169.3, 163.1 (C-3, C-4), 161.7, 160.1 (C-2‴, C-7‴), 154.7, 153.4, 138.1, 129.2, 129.1, 126.4, 119.6, 113.0, 112.4, 111.0 (Ar-C), 101.1 (C-8‴), 68.1 (OCH_2_), 43.6 (NCH_2_), 30.2 (CH_2_), 28.0 (CH_2_), 22.3 (CH_2_), 18.1 (CH_3_) ppm; HRESI-MS *m*/*z* calcd. for C_25_H_23_ClN_2_NaO_5_ ([M + Na]^+^): 489.1188, found: 489.1186.

3-[(4′-Bromophenyl)amino]-4-{[5″-(4‴-methyl-2‴-oxo-2‴*H*-chromen-7‴-yl)oxypentyl]amino}cyclobut-3-ene-1,2-dione (**16h**). Method A. Dimethyl squarate (50 mg, 0.35 mmol), 4-bromoaniline (61 mg, 0.35 mmol) and **15** (92 mg, 0.35 mmol, *n* = 5, R^2^ = Me, R^3^ = H) were used. Yield: 111 mg (62%, light yellow solid). Mp: 248–249 °C. *R*_f_ 0.10 (1:1 cyclohexane-EtOAc). ^1^H-NMR (300 MHz, DMSO-*d*_6_) δ 9.67 (s, 1H, N*H*-Ar), 7.67–7.63 ( brs, 1H, NH), 7.64 (d, 1H, *J*_5‴,6‴_ = 8.7 Hz, H-5‴), 7.49 (m, 2H, Ar-H), 7.37 (m, 2H, Ar-H), 6.96–6.92 (m, 2H, Ar-H), 6.19 (brd, 1H, *J*_H,H_ = 1.0 Hz, H-3‴), 4.10 (t, 2H, *J*_H,H_ = 6.4 Hz, OCH_2_), 3.63 (brq, 2H, *J*_H,H_ = 7.1 Hz, NCH_2_), 2.38 (brd, 3H, *J*_H,H_ = 0.8 Hz CH3), 1.78 (quint, 2H, *J*_H,H_ = 6.8 Hz, CH_2_), 1.65 (quint, 2H, *J*_H,H_ = 7.3 Hz, CH_2_), 1.49 (m, 2H, CH_2_) ppm; ^13^C-NMR (125.7 MHz, DMSO-*d*_6_) δ 184.2, 180.1 (C=O), 169.3, 163.0 (C-3, C-4), 161.7, 160.1 (C-2‴, C-7‴), 154.7, 153.4, 138.5, 132.1, 126.4, 120.0, 114.3, 113.0, 112.4, 111.0 (Ar-C), 101.1 (C-8‴), 68.1 (OCH_2_), 43.6 (NCH_2_), 30.2 (CH_2_), 28.0 (CH_2_), 22.3 (CH_2_), 18.1 (CH_3_) ppm; HRESI-MS *m*/*z* calcd. for C_25_H_23_^79^BrN_2_NaO_5_ ([M + Na]^+^): 533.0683, found: 533.0672.

3-[(4′-Iodophenyl)amino]-4-{[5″-(4‴-methyl-2‴-oxo-2‴*H*-chromen-7‴-yl)oxypentyl]amino}cyclobut-3-ene-1,2-dione (**16i**). Method A. Dimethyl squarate (50 mg, 0.35 mmol), 4-yodoaniline (77 mg, 0.35 mmol) and **15** (92 mg, 0.35 mmol, *n* = 5, R^2^ = Me, R^3^ = H) were used. Yield: 125 mg (64%, light yellow solid). Mp > 250 °C. *R*_f_ 0.20 (1:1 cyclohexane-EtOAc). ^1^H-NMR (300 MHz, DMSO-*d*_6_) δ 9.67 (s, 1H, N*H*-Ar), 7.65 (m, 4H, H-5‴,NH, 2Ar-H), 7.25 (m, 2H, Ar-H), 6.97–6.92 (m, 2H, Ar-H), 6.20 (brd, 1H, *J*_H,H_ = 1.1 Hz, H-3‴), 4.10 (t, 2H, *J*_H,H_ = 6.4 Hz, OCH_2_), 3.64 (brq, 2H, *J*_H,H_ = 6.2 Hz, NCH_2_), 2.39 (brd, 3H, *J*_H,H_ = 1.0 Hz, CH_3_), 1.79 (quint, 2H, *J*_H,H_ = 7.2 Hz, CH_2_), 1.65 (quint, 2H, *J*_H,H_ = 7.3 Hz, CH_2_), 1.50 (m, 2H, CH_2_) ppm; ^13^C-NMR (125.7 MHz, DMSO-*d*_6_) δ 184.2, 180.1 (C=O), 169.3, 163.0 (C-3, C-4), 161.7, 160.1 (C-2‴, C-7‴), 154.7, 153.4, 138.9, 137.8, 126.4, 120.3, 113.0, 112.4, 111.0 (Ar-C), 101.1 (C-8‴), 85.9 (C-4′), 68.1 (OCH_2_), 43.6 (NCH_2_), 30.2 (CH_2_), 28.0 (CH_2_), 22.3 (CH_2_), 18.1 (CH_3_) ppm; HRESI-MS *m*/*z* calcd. for C_25_H_23_IN_2_NaO_5_ ([M + Na]^+^): 581.0544, found: 581.0543.

3-{[5′-((3″,4″-Dimethyl-2″-oxo-2″*H*-chromen-7″-yl)oxy)pentyl]amino}-4-phenylaminocyclobut-3-ene-1,2-dione (**16j**). Method B. Dimethyl squarate (51 mg, 0.36 mmol), aniline (33 µL, 0.36 mmol), and **15** (100 mg, 0.36 mmol, *n* = 5, R^2^ = R^3^ = Me) were used. Yield: 71 mg (44%, light yellow solid). Mp > 250 °C. *R*_f_ 0.40 (1:1 cyclohexane-EtOAc). ^1^H-NMR (300 MHz, DMSO-*d*_6_) δ 9.60 (s, 1H, N*H*-Ar), 7.67–7.63 (m, 1H, NH), 7.66 (d, 1H, *J*_5″,6″_ = 8.5 Hz, H-5″), 7.44–7.30 (m, 4H, Ar-H), 7.01 (t, 1H, *J*_H,H_ = 7.3 Hz, H-4, Ph), 6.94–6.91 (m, 2H, Ar-H), 4.09 (brt, 2H, *J*_H,H_ = 6.1 Hz, OCH2), 3.63 (brs, 2H, NCH_2_), 2.35 (brs, 3H, CH_3_), 2.07 (brs, 3H, CH_3_), 1.78 (brquint, 2H, *J*_H,H_ = 7.0 Hz, CH_2_), 1.64 (brquint, 2H, *J*_H,H_ = 7.0 Hz, CH_2_), 1.51 (brquint, 2H, *J*_H,H_ = 7.0 Hz, CH_2_) ppm; ^13^C-NMR (125.7 MHz, DMSO-d6) δ 184.0, 180.1 (C=O), 169.2, 163.5 (C-3, C-4), 161.2, 160.7 (C-2″, C-7″), 153.0, 146.8, 139.0, 129.3, 126.1, 122.5, 117.9, 117.8, 113.5, 112.2 (Ar-C), 100.8 (C-8″), 68.0 (OCH_2_), 43.6 (NCH_2_), 30.2 (CH_2_), 28.0 (CH_2_), 22.4 (CH_2_), 14.9 (CH_3_), 12.9 (CH_3_) ppm; HRESI-MS *m*/*z* calcd. for C_26_H_26_N_2_NaO_5_ ([M + Na]^+^): 469.1734, found: 469.1727.

3-{[5′-(3″,4″-Dimethyl-2″-oxo-2″*H*-chromen-7″-yl)oxypentyl]amino}-4-(4‴-methoxyphenylamino)cyclobut-3-ene-1,2-dione (**16k**). Method B. Dimethyl squarate (51 mg, 0.36 mmol), 4-methoxyaniline (45 mg, 0.36 mmol), and **15** (100 mg, 0.36 mmol, *n* = 5, R^2^ = R^3^ = Me) were used. Yield: 69 mg (40%, light green solid). Mp > 250 °C. *R*_f_ 0.20 (1:1 cyclohexane-EtOAc). ^1^H-NMR (300 MHz, DMSO-d6) δ 9.49 (brs, 1H, N*H*-Ar), 7.65–7.33 (m, 4H, NH, H-5″, 2Ar-H), 6.92 (m, 4H, Ar-H), 4.08 (brs, 2H, NCH_2_), 3.72–3.61 (m, 5H, OCH_2_, OMe), 2.35 (brs, 3H, CH_3_), 2.06 (brs, 3H, CH_3_), 1.77 (m, 2H, CH_2_), 1.63 (m, 2H, CH_2_), 1.50 (m, 2H, CH_2_) ppm; ^13^C-NMR (125.7 MHz, DMSO-d6) δ 183.4, 180.3 (C=O), 168.8, 163.5 (C-3, C-4), 161.2, 160.6 (C-2″, C-7″), 155.1, 153.0, 146.8, 132.2, 126.0, 120.0, 119.5, 117.7, 114.5, 113.5, 112.2 (Ar-C), 100.8 (C-8″), 67.9 (OCH_2_), 55.2 (OCH_3_), 43.5 (NCH_2_), 30.3 (CH_2_), 28.0 (CH_2_), 22.4 (CH_2_), 14.8 (CH_3_), 12.8 (CH_3_) ppm; HRESI-MS *m*/*z* calcd. for C_2_7H_28_N_2_NaO_6_ ([M + Na]^+^): 499.1840, found: 499.1832.

3-{[5′-(3″,4″-Dimethyl-2″-oxo-2″*H*-chromen-7″-yl)oxypentyl]amino}-4-(4‴-fluorophenylamino)cyclobut-3-ene-1,2-dione (**16l**). Method B. Dimethyl squarate (51 mg, 0.36 mmol), 4-fluoroaniline (35 µL, 0.36 mmol), and **15** (100 mg, 0.36 mmol, *n* = 5, R^2^ = R^3^ = Me) were used. Yield: 67 mg (40%, yellow solid). Mp > 250 °C. *R*_f_ 0.30 (1:1 cyclohexane-EtOAc). ^1^H-NMR (300 MHz, DMSO-d6) δ 9.61 (s, 1H, N*H*-Ar), 7.70–7.61 (brs, 1H, NH), 7.66 (brd, 1H, J5″,6″= 8.3 Hz, H-5″), 7.43 (m, 2H, Ar-H), 7.22–7.15 (m, 2H, Ar-H), 6.94 (m, 2H, Ar-H), 4.09 (brt, 2H, *J*_H,H_ = 6.5 Hz, OCH_2_), 3.62 (brs, 2H, NCH_2_), 2.36 (brs, 3H, CH_3_), 2.07 (brs, 3H, CH_3_), 1.79 (brquint, 2H, *J*_H,H_ = 6.7 Hz, CH_2_), 1.64 (brquint, 2H, *J*_H,H_ = 7.1 Hz, CH_2_), 1.50 (m, 2H, CH_2_) ppm; ^13^C-NMR (125.7 MHz, DMSO-d6) δ 183.8, 180.2 (C=O), 169.1, 163.4 (C-3, C-4), 161.2, 160.6 (C-2″, C-7″), 158.0 (d, ^1^*J*_C,F_ =238.2 Hz, C-4‴), 153.0, 146.8 (Ar-C), 135.5 (d, ^4^*J*_C,F_= 2.7 Hz, C-1‴), 126.1 (Ar-C), 119.7 (brd, ^3^*J*_C,F_ = 6.2 Hz, C-2‴), 117.7 (Ar-C), 115.9, (d, ^2^*J*_C,F_ = 22.6 Hz, C-3‴), 113.5, 112.2 Ar-C), 100.8 (C-8″), 67.9 (OCH_2_), 43.6 (NCH_2_), 30.2 (CH_2_), 28.0 (CH_2_), 22.3 (CH_2_), 14.8 (CH_3_), 12.8 (CH_3_) ppm; HRESI-MS *m*/*z* calcd. for C_26_H_25_FN_2_NaO_5_ ([M + Na]^+^): 487.1640, found: 487.1630.

3-[(4′-Chlorophenyl)amino]-4-{[5″-(3‴,4‴-dimethyl-2‴-oxo-2‴*H*-chromen-7‴-yl)oxypentyl]amino}cyclobut-3-ene-1,2-dione (**16m**). Method B. Dimethyl squarate (51 mg, 0.36 mmol), 4-chloroaniline (46 mg, 0.36 mmol), and **15** (100 mg, 0.36 mmol, *n* = 5, R^2^ = R^3^ = Me) were used. Yield: 54 mg (31%, yellow solid). Mp > 250 °C. *R*_f_ 0.10 (1:1 cyclohexane-EtOAc). ^1^H-NMR (300 MHz, DMSO-d6) δ 9.68 (s, 1H, N*H*-Ar), 7.69–7.63 (m, 1H, NH), 7.65 (d, 1H, *J*_5‴,6‴_ = 9.4 Hz, H-5‴), 7.44-7.33 (m, 4H, Ar-H), 6.92–6.90 (m, 2H, Ar-H), 4.08 (t, 2H, *J*_H,H_ = 6.3 Hz, OCH_2_), 3.63 (brq, 2H, *J*_H,H_ = 6.9 Hz, NCH_2_), 2.34 (brs, 3H, CH_3_), 2.06 (brs, 3H, CH_3_), 1.78 (quint, 2H, *J*_H,H_ = 7.1 Hz, CH_2_), 1.65 (quint, 2H, *J*_H,H_ = 6.8 Hz, CH_2_), 1.49 (quint, 2H, *J*_H,H_ = 7.2 Hz, CH_2_) ppm; ^13^C-NMR (125.7 MHz, DMSO-d6) δ 184.1, 180.1 (C=O), 169.3, 163.1 (C-3, C-4), 161.2, 160.6 (C-2‴, C-7‴), 153.0, 146.8, 138.1, 129.1, 129.0, 126.1, 119.5, 117.7, 113.5, 112.2 (Ar-C), 100.8 (C-8‴), 67.9 (OCH_2_), 43.6 (NCH_2_), 30.2 (CH_2_), 28.0 (CH_2_), 22.3 (CH_2_), 14.8 (CH3), 12.8 (CH3) ppm; HRESI-MS *m*/*z* calcd. for C_26_H_25_ClN_2_NaO_5_ ([M + Na]^+^): 503.1344, found: 503.1338.

3-[(4′-Bromophenyl)amino]-4-{[5″-(3‴,4‴-dimethyl-2‴-oxo-2‴*H*-chromen-7″-yl)oxypentyl]amino}cyclobut-3-ene-1,2-dione (**16n**). Method B. Dimethyl squarate (51 mg, 0.36 mmol), 4-bromoaniline (62 mg, 0.36 mmol), and **15** (100 mg, 0.36 mmol, *n* = 5, R^2^ = R^3^ = Me) were used. Yield: 49 mg (26%, yellow solid). Mp >250 ⁰C. *R*_f_ 0.10 (1:1 cyclohexane-EtOAc). ^1^H-NMR (300 MHz, DMSO-d6) δ 9.87 (s, 1H, N*H*-Ar), 7.70–7.64 (m, 1H, NH), 7.65 (d, 1H, *J*_5‴,6‴_ = 8.8 Hz, H-5‴), 7.54–7.35 (m, 4H, Ar-H), 6.93–6.91 (m, 2H, Ar-H), 4.08 (brt, 2H, *J*_H,H_ = 6.0 Hz, OCH_2_), 3.63 (m, 2H, NCH_2_), 2.35 (brs, 3H, CH_3_), 2.07 (brs, 3H, CH_3_), 1.78 (brquint, 2H, *J*_H,H_ = 6.3 Hz, CH_2_), 1.65 (brquint, 2H, *J*_H,H_ = 6.3 Hz, CH_2_), 1.49 (brquint, 2H, *J*_H,H_ = 7.0 Hz, CH_2_) ppm; ^13^C-NMR (125.7 MHz, DMSO-d6) δ 184.1, 180.1 (C=O), 169.3, 163.0 (C-3, C-4), 161.2, 160.6 (C-2‴, C-7‴), 153.0, 146.8, 138.4, 132.0, 126.0, 119.0, 117.7, 114.3, 113.4, 112.2 (Ar-C), 100.8 (C-8‴), 67.9 (OCH_2_), 43.6 (NCH_2_), 30.2 (CH_2_), 28.0 (CH_2_), 22.3 (CH_2_), 14.8 (CH_3_), 12.8 (CH_3_) ppm; HRESI-MS *m*/*z* calcd. for C_26_H_25_^79^BrN_2_NaO_5_ ([M + Na]^+^): 547.0839, found: 547.0826; *m*/*z* calcd. for C_26_H_25_^81^BrN_2_NaO_5_ ([M + Na]^+^): 549.0819, found: 549.0805. 

3-{[5′-(3″,4″-Dimethyl-2″-oxo-2″*H*-chromen-7″-yl)oxypentyl]amino}-4-(4‴-iodophenylamino)cyclobut-3-ene-1,2-dione (**16o**). Method B. Dimethyl squarate (51 mg, 0.36 mmol), 4-iodoaniline (79 mg, 0.36 mmol), and **15** (100 mg, 0.36 mmol, *n* = 5, R^2^ = R^3^ = Me) were used. Yield: 74 mg (36%, yellow solid). Mp > 250 °C. *R*_f_ 0.20 (1:1 cyclohexane-EtOAc). ^1^H-NMR (300 MHz, DMSO-d6) δ 9.64 (s, 1H, N*H*-Ar), 7.70–7.62 (m, 4H, NH, H-5″, 2Ar-H), 7.27–7.22 (m, 2H, Ar-H), 6.93–6.90 (m, 2H, Ar-H), 4.07 (brt, 2H, *J*_H,H_ = 6.1 Hz, OCH_2_), 3.62 (brs, 2H, NCH_2_), 2.34 (brs, 3H, CH_3_), 2.06 (brs, 3H, CH_3_), 1.77 (brquint, 2H, *J*_H,H_ = 6.5 Hz, CH_2_), 1.62 (brquint, 2H, *J*_H,H_ = 6.5 Hz, CH_2_), 1.49 (quint, 2H, *J*_H,H_ = 6.7 Hz, CH_2_) ppm; ^13^C-NMR (125.7 MHz, DMSO-d6) δ 184.2, 180.1 (C=O), 169.3, 163.0 (C-3, C-4), 161.2, 160.6 (C-2″, C-7″), 153.0, 146.8, 138.9, 137.9, 137.8, 126.1, 120.3, 117.7, 113.5, 112.2 (Ar-C), 100.8 (C-8″), 85.9 (C-4‴), 67.9 (OCH_2_), 43.6 (NCH_2_), 30.2 (CH_2_), 28.0 (CH_2_), 22.3 (CH_2_), 14.9 (CH_3_), 12.9 (CH_3_) ppm; HRESI-MS *m*/*z* calcd. for C_26_H_25_IN_2_NaO_5_ ([M + Na]^+^): 595.0700, found: 595.0690.

3-Phenylamino-4-{[5′-(4″-Phenyl-2″-oxo-2″*H*-chromen-7″-yl)oxypentyl]amino}cyclobut-3-ene-1,2-dione (**16p**). Method C. Dimethyl squarate (59 mg, 0.41 mmol), aniline (38 µL, 0.41 mmol), and **15** (132.6 mg, 0.41 mmol, *n* = 5, R^2^ = Ph, R^3^ = H) were used. Yield: 130 mg (64%, yellow solid). Mp: 209–210 °C. *R*_f_ 0.20 (1:1 cyclohexane-EtOAc). ^1^H-NMR (300 MHz, DMSO-d6) δ 9.61 (brs, 1H, N*H*-Ar), 7.67 (brs, 1H, NH), 7.58–7.49 (m, 5H, H-5″, 4Ar-H), 7.42 (m, 2H, Ar-H), 7.35–7.29 (m, 3H, Ar-H), 7.08 (d, 1H, *J*_6″,8″_ = 2.4 Hz, H-8″), 7.01 (brt, 1H, *J*_H,H_ = 7.3 Hz, H-4 Ph), 6.91 (dd, 1H, *J*_6″,8″_ = 2.4 Hz, *J*_5″,6″_ = 8.9 Hz H-6″), 6.22 (brs, 1H, H-3″), 4.11 (t, 2H, *J*_H,H_ = 6.3 Hz, OCH_2_), 3.64 (brq, 2H, *J*_H,H_ = *J*_NH,CH3_ = 6.7 Hz, NCH_2_), 1.79 (quint, 2H, *J*_H,H_ = 6.9 Hz, CH_2_), 1.65 (quint, 2H, *J*_H,H_ = 7.3 Hz, CH_2_), 1.50 (quint, 2H, *J*_H,H_ = 6.5 Hz, CH_2_) ppm; ^13^C-NMR (125.7 MHz, DMSO-d6) δ 184.0, 180.1 (C=O), 169.2, 163.5 (C-3, C-4), 161.9, 160.0 (C-2″, C-7″), 155.5, 155.2, 139.0, 135.0, 129.6, 129.3, 128.8, 128.4, 127.8, 122.5, 117.9, 112.8, 111.7, 111.2 (Ar-C), 101.6 (C-8″), 68.2 (OCH_2_), 43.6 (NCH_2_), 30.3 (CH_2_), 28.0 (CH_2_), 22.3 (CH_2_) ppm; HRESI-MS *m*/*z* calcd. for C_30_H_26_N_2_NaO_5_ [M + Na]^+^): 517.1734, found: 517.1730.

### 3.2. CA Inhibition Assays

The inhibitory properties of title compounds against CAs were determined using the stopped-flow CO_2_ hydrase assay, as previously reported [40,41]. All enzymes employed were recombinant, and obtained in-house as reported [40,41,42], with concentrations in the assay ranging from 5–12 nM.

### 3.3. Antiproliferative Assays

Minor modifications of the US National Cancer Institute (NCI) protocol were used [76].

### 3.4. Docking Simulations

Previously reported methodology was used [77]. For sulfonamides, deprotonated species were considered.

## 4. Conclusions

A vast series of novel CA inhibitors have been accessed in a straightforward fashion by combining a pharmacophore targeting CA inhibition (arylsulfonamide, coumarin) and an aromatic hydrophobic appendage through a squaramide-type tether.

Considering the inhibition of tumor-associated CAs IX and XII, sulfonamides exhibited considerably lower selectivity toward off-target enzymes (CAs I and II) than coumarin counterparts. Conjugating potency and selectivity, the best profile for *p*-substituted sulfonamides was found for compound **5d** (*p*-bromophenyl derivative), with activities within the mid-nM range against CAs IX and XII (*K*_i_ = 67.6 and 85.5 nM, respectively), and good selectivity indexes (S.I. 6.8–14). Preparation of a dimer (**6**) strongly diminished selectivity. Shortening the distance between the squaramido and the aryl sulfonamido motifs furnished improved potency against targeted CAs; when this was accompanied by location of the sulfonamido functionality on the m-position (compound **11**), the lead compound of the sub-series was achieved (low nM activities for CA XII (9.5 nM) and improved selectivities compared to AAZ; S.I. 10–63 vs. 0.48–44).

Coumarin derivatives showed in all cases outstanding selectivities (*K*_i_ > 10 µM for CA I, II). Elongation of the hydrocarbon chain connecting the coumarin skeleton provoked a clear improvement in activity against CAs IX and XII, reaching low nanomolar activities for n = 9, 12; *K*_i_ values against CA IX and CA XII were found to be 19.2 and 7.23 nM for compound **16c** and 18.1, 7.91 nM for **16d**, the lead derivatives of the series. Stereoelectronic effects on the appended phenyl residue had little influence on the inhibitory profiles; moreover, increase in the steric hindrance on C-3 and/or C-4 positions of the coumarin moiety had a detrimental effect.

The main interactions involving the most favorable poses of the lead compounds of the series within the catalytic active site of CA XII were predicted using docking simulations.

Although compounds prepared herein were not active against tumor cells (measured under normoxic conditions), it would be expected that in vivo treatment, where numerous tumor cells overexpress CA IX/XII isoforms as an efficient survival mechanism (hypoxic conditions), antiproliferative activities would be observed.

## Figures and Tables

**Table 1:** Inhibition constants and selectivity indexes of sulfonamido-containing squaramides against hCAs I, II, IX and XII ^a^.

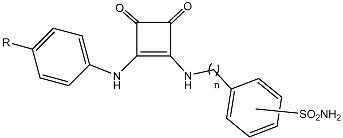								
**Compound**	***K*_i_ (nM)**				**S.I.** ^b^			
	**hCA I**	**hCA II**	**hCA IX**	**hCA XII**	**I/IX**	**I/XII**	**II/IX**	**II/XII**
**5a** R = H, *n* = 2, *p*-SO_2_NH_2_	211	720	75.3	96.8	2.8	2.2	9.6	7.4
**5b** R = OMe, *n* = 2, *p*-SO_2_NH_2_	373	724	92.5	158	4.0	2.4	7.8	4.6
**5c** R = Cl, *n* = 2, *p*-SO_2_NH_2_	881	819	490	90.2	1.8	9.8	1.7	9.1
**5d** R = Br, *n* = 2, *p*-SO_2_NH_2_	457	910	67.6	85.5	6.8	5.3	14	11
**5e** R = I, *n* = 2, *p*-SO_2_NH_2_	471	856	92.7	90.3	5.1	5.2	9.2	9.5
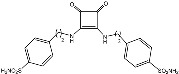 **6**	43.2	56.2	83.0	87.8	0.52	0.49	0.68	0.64
**8** R = H, *n* = 0, *p*-SO_2_NH_2_	98.0	65.9	19.5	6.57	5.0	15	3.4	10
** 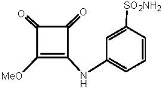 ** **10**	79.9	40.9	26.2	5.78	3.0	14	1.6	7.1
**11** R = H, *n* = 0, *m*-SO_2_NH_2_	580	95.5	29.4	9.15	20	63	3.2	10
** 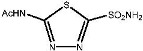 ** **AAZ**	250.0	12.0	25.0	5.7	10	44	0.48	2.1

^a^ Mean from 3 different assays, by a stopped flow technique (errors were in the range of ±5–10% of the reported values); ^b^ S.I. = *K*_i_ (CA I or II)/*K*_i_ (CA XI or XII).

**Table 2:** Inhibition constants of coumarin-containing squaramides **16** against hCAs I, II, IX and XII ^a,b^.

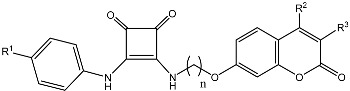				
**Compound**	***K*_i_ (nM)**			
	**hCA I**	**hCA II**	**hCA IX**	**hCA XII**
**16a** R^1^ = R^3^ = H, R^2^ = Me, *n* = 3	>10,000	>10,000	79.3	42.1
**16b** R^1^ = R^3^ = H, R^2^ = Me, *n* = 5	>10,000	>10,000	44.1	14.3
**16c** R^1^ = R^3^ = H, R^2^ = Me, *n* = 9	>10,000	>10,000	19.2	7.23
**16d** R^1^ = R^3^ = H, R^2^ = Me, *n* = 12	>10,000	>10,000	18.1	7.91
**16e** R^1^ = OMe, R^2^ = Me, R^3^ = H, *n* = 5	>10,000	>10,000	59.7	19.5
**16f** R^1^ = F, R^2^ = Me, R^3^ = H, *n* = 5	>10,000	>10,000	43.7	15.9
**16g** R^1^ = Cl, R^2^ = Me, R^3^ = H, *n* = 5	>10,000	>10,000	65.3	24.4
**16h** R^1^ = Br, R^2^ = Me, R^3^ = H, *n* = 5	>10,000	>10,000	46.2	13.9
**16i** R^1^ = I, R^2^ = Me, R^3^ = H, *n* = 5	>10,000	>10,000	57.0	21.5
**16j** R^1^ = H, R^2^ = R^3^ = Me, *n* = 5	>10,000	>10,000	107	58.3
**16k** R^1^ = OMe, R^2^ = R^3^ = Me, *n* = 5	>10,000	>10,000	86.5	45.4
**16l** R^1^ = F, R^2^ = R^3^ = Me, *n* = 5	>10,000	>10,000	113	62.9
**16m** R^1^ = Cl, R^2^ = R^3^ = Me, *n* = 5	>10,000	>10,000	96.6	43.0
**16n** R^1^ = Br, R^2^ = R^3^ = Me, *n* = 5	>10,000	>10,000	132	69.6
**16o** R^1^ = I, R^2^ = R^3^ = Me, *n* = 5	>10,000	>10,000	92.1	50.8
**16p** R^1^ = R^3^ = H, R^2^ = Ph, *n* = 5	>10,000	>10,000	267	87.9

^a^ Mean from three different assays, by a stopped flow technique (errors were in the range of ±5–10% of the reported values); ^b^ incubation time 6 h.

**Table 3:** Docking interaction energy of **16c**-CA XII complex.

**Structure**	Non-Hydrolyzed Form	Hydrolyzed Form at the Cavity	Hydrolyzed Form Outside the Cavity
**Energy (kcal/mol)**	−7.24	−10.00	−6.90

**Table 4:** Predicted physicochemical properties for squaramides derivatives.

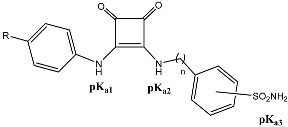										
**Compound**	**Physicochemical Properties**									
	***M*** **_w_** ** (g/mol)**	**Consensus logP**	**logS** **(Ali)**	**TPSA (Å^2^)**	**#H-Bond Acceptor**	**#H-Bond Donor**	**Druglikeness** **(Lipinski)**	**pK_a1_**	**pK_a2_**	**pK_a3_**
**5a**	371.41	1.27	−4.33 (m.s.) ^a^	126.74	5	3	Yes	10.2	10.8	10.0
**5b**	401.44	1.26	−4.49 (m.s.)	135.97	6	3	Yes	10.2	10.8	10.0
**5c**	405.86	1.87	−4.97 (m.s.)	126.74	5	3	Yes	9.9	10.7	10.0
**5d**	450.31	1.96	−5.05 (m.s.)	126.74	5	3	Yes	9.9	10.7	10.0
**5e**	497.31	1.93	−5.00 (m.s.)	126.74	5	3	Yes	10.0	10.7	10.0
**6**	478.54	0.37	−4.69 (m.s.)	195.28	8	4	Yes	9.7	9.7	9.8
**8**	343.36	0.88	−3.91 (s.) ^b^	126.74	5	3	Yes	10.7	10.3	10.2
**10**	282.27	−0.16	−2.33 (s.)	123.94	6	2	Yes	-	10.3	10.0
**11**	343.36	0.89	−3.91 (s.)	126.74	5	3	Yes	10.7	10.2	9.9
**16a**	404.42	2.81	−5.17 (m.s.)	97.64	5	2	Yes	10.4	11.5	-
**16b**	432.47	3.45	−5.92 (m.s.)	97.64	5	2	Yes	10.4	11.7	-
**16c**	488.57	4.88	−7.97 (p.s.) ^c^	97.64	5	2	Yes	10.4	11.8	-
**16d**	530.65	5.98	−9.65 (p.s.)	97.64	5	2	Yes (1 violation *M*_w_ > 500)	10.4	11.8	-
**16e**	462.49	3.49	−6.08 (p.s.)	106.87	6	2	Yes	10.5	11.7	-
**16f**	450.46	3.79	−6.02 (p.s.)	97.64	6	2	Yes	10.3	11.6	-
**16g**	466.91	4.06	−6.57 (p.s.)	97.64	5	2	Yes	10.2	11.6	-
**16h**	511.36	4.17	−6.63 (p.s.)	97.64	5	2	Yes (1 violation *M*_w_ > 500)	10.2	11.6	-
**16i**	558.36	4.16	−6.59 (p.s.)	97.64	5	2	Yes (1 violation *M*_w_ > 500)	10.2	11.6	-
**16j**	446.50	3.87	−6.30 (p.s.)	97.64	5	2	Yes	10.4	11.7	-
**16k**	476.52	3.81	−6.47 (p.s.)	106.87	6	2	Yes	10.5	11.7	-
**16l**	464.49	4.15	−6.41 (p.s.)	97.64	6	2	Yes	10.3	11.6	-
**16m**	480.94	4.36	−6.96 (p.s.)	97.64	5	2	Yes	10.2	11.5	-
**16n**	525.39	4.44	−7.02 (p.s.)	97.64	5	2	Yes (1 violation *M*_w_ > 500)	10.2	11.6	-
**16o**	572.39	4.52	−6.98 (p.s.)	97.64	5	2	Yes (1 violation *M*_w_ > 500)	10.2	11.6	-
**16p**	494.54	4.51	−7.23 (p.s.)	97.64	5	2	Yes	10.4	11.7	-
**AAZ**	222.25	−0.62	−2.47 (s.)	151.66	6	2	Yes	-	-	7.2

^a^ m.s.: moderately soluble; ^b^ s.: soluble; ^c^ p.s.: poorly soluble.

## Data Availability

Not applicable.

## Supplementary Materials

The following supporting information can be downloaded at: https://www.mdpi.com/article/10.3390/ijms23147685/s1.
