# Supplementary material for: Squaramide-Tethered Sulfonamides and Coumarins: Synthesis, Inhibition of Tumor-Associated CAs IX and XII and Docking Simulations
Source: Int J Mol Sci. 2022 Jul 12;23(14):7685. doi: 10.3390/ijms23147685 (PMC9318203; doi:10.3390/ijms23147685)
Supplement: Supplementary file 1 [file ijms-23-07685-s001.zip › ijms-1776771-supplementary.pdf]

Squaramide-Tethered Sulfonamides and Coumarins: Synthesis, Inhibition of Tumor-Associated CAs IX and XII and Docking Simulations

Giulia Arrighi [1], Adrián Puerta [2], Andrea Petrini [3], Francisco J. Hicke [1], Alessio Nocentini [3], Miguel X. Fernandes [2], José M. Padrón [2], Claudiu T. Supuran [3,*], José G. Fernández-Bolaños [1] and Óscar López [1,*]

[1] Departamento de Química Orgánica, Facultad de Química, Universidad de Sevilla, Apartado 1203, E-41071 Seville, Spain; giulia.arrighi1@stud.unifi.it (G.A.); javipoke@hotmail.es (F.J.H.); bolanos@us.es (J.G.F.-B.)

[2] BioLab, Instituto Universitario de Bio-Orgánica Antonio González (IUBO-AG), Universidad de La Laguna, c/Astrofísico Francisco Sánchez 2, E-38206 La Laguna, Spain; apuertaa@ull.es (A.P.); mfernand@ull.es (M.X.F.); jmpadron@ull.es (J.M.P.)

[3] NEUROFARBA Department, Sezione di Scienze Farmaceutiche e Nutraceutiche, University of Florence, 50019 Florence, Italy; andrea.petreni@unifi.it (A.P.); alessio.nocentini@unifi.it (A.N.)

* Correspondence: claudiu.supuran@unifi.it (C.T.S.); osc-lopez@us.es (Ó.L.)

^{1}H- and ^{13}C-NMR spectra of compounds 5, 6, 8, 10, 11, 16..S2–S53

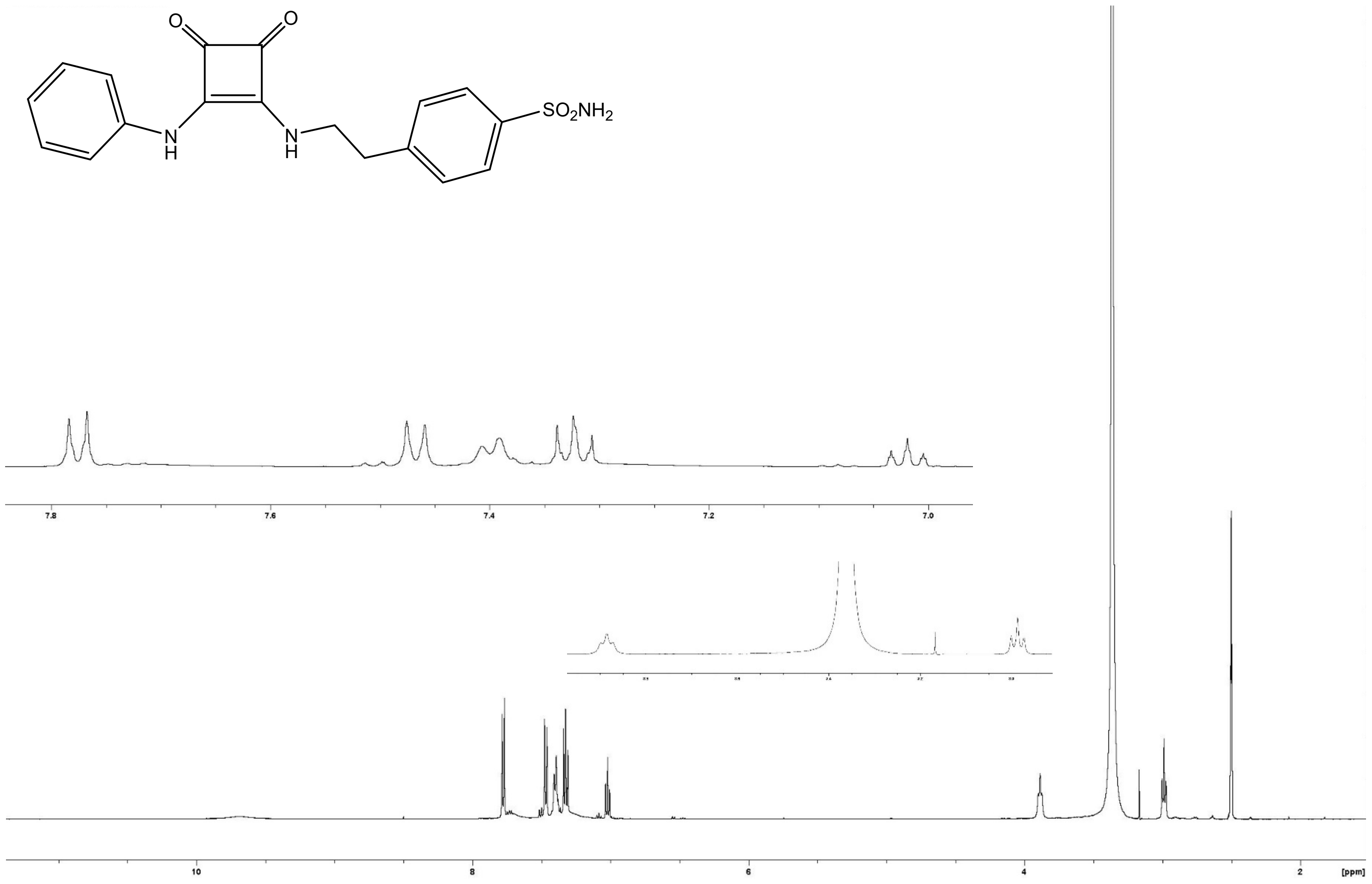

^{1}H-NMR (500 MHz, $(CD_3)_2SO$) of **5a**

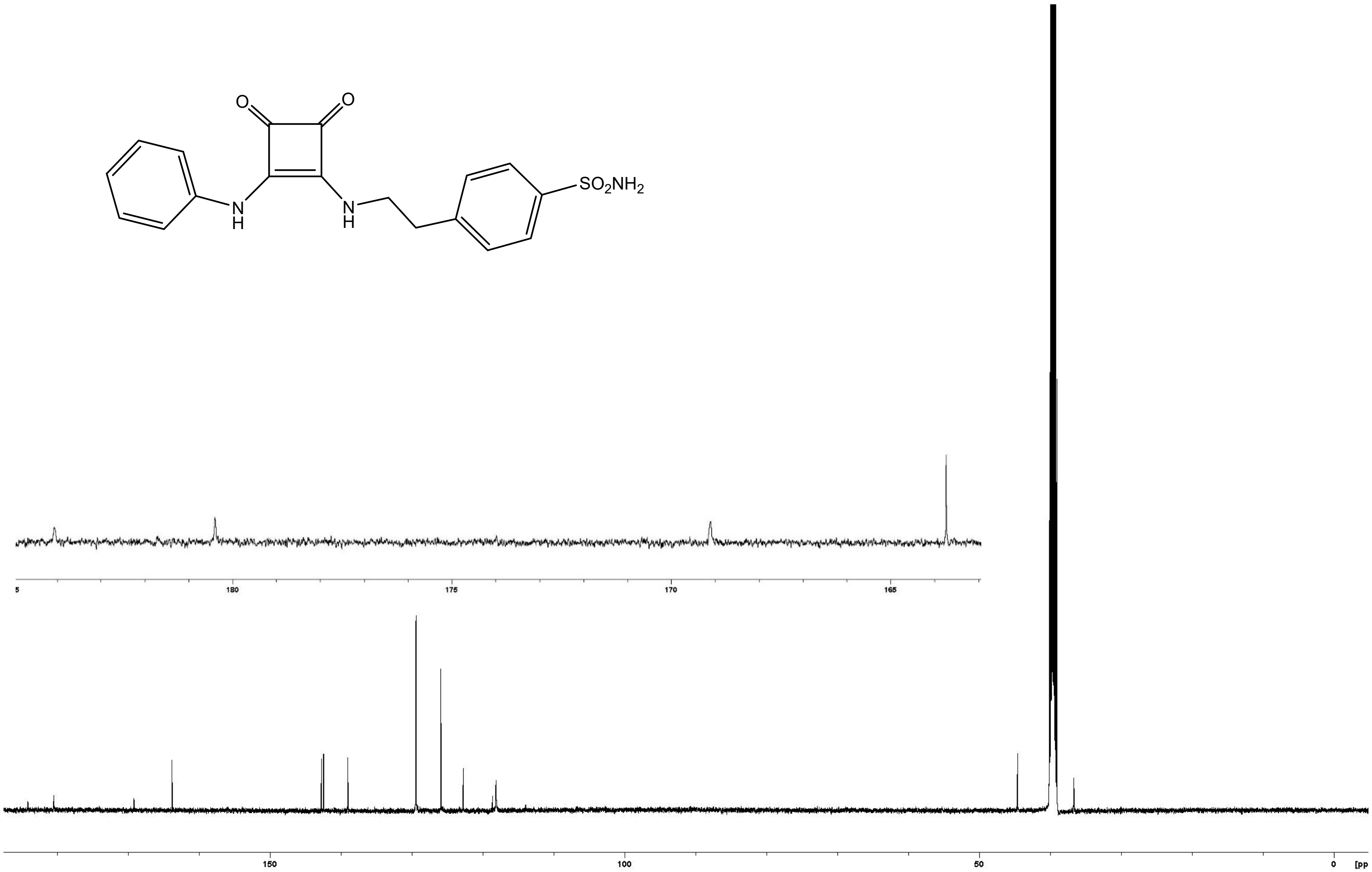

^{13}C-NMR (125.7 MHz, $(CD_3)_2SO$) of **5a**

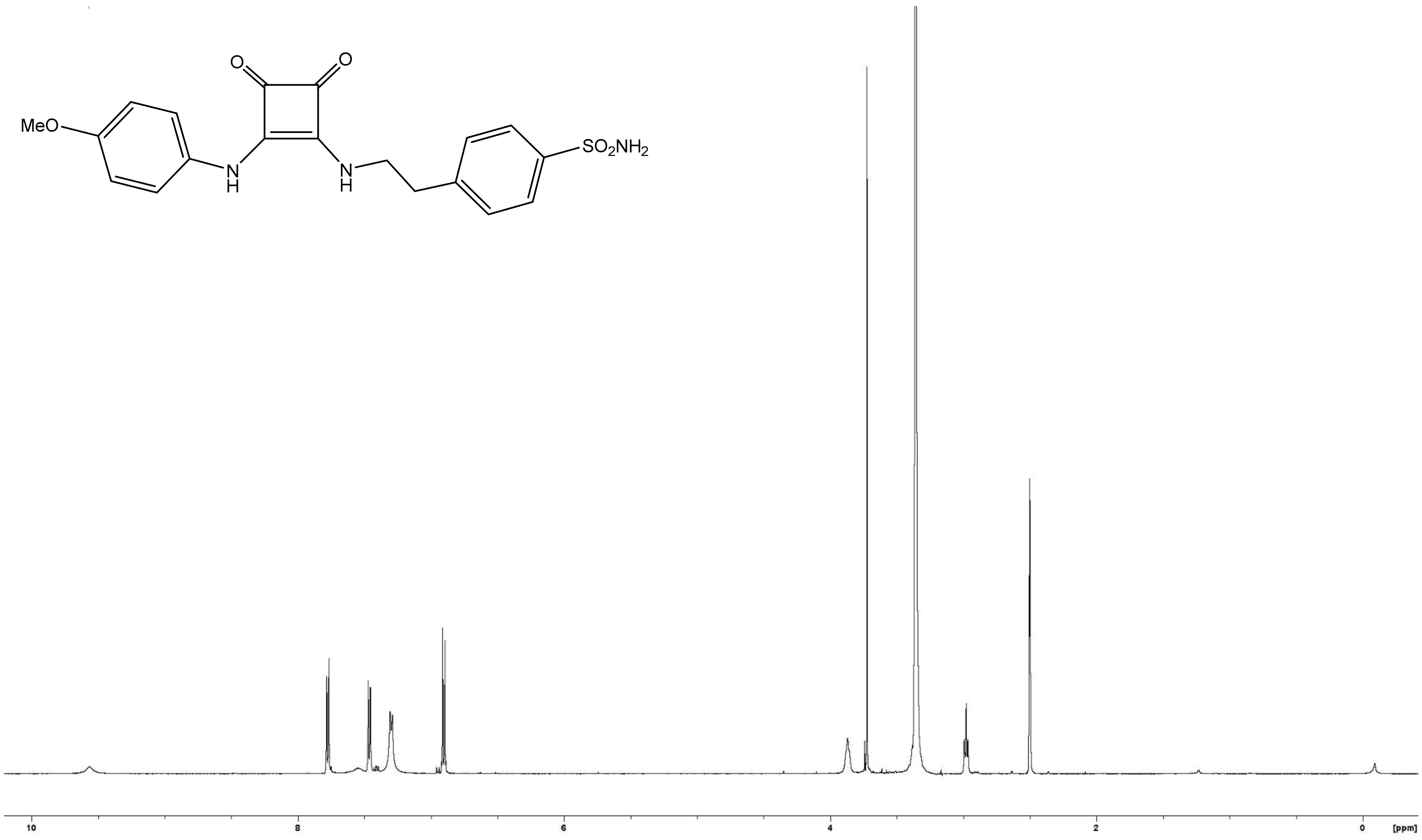

^{1}H-NMR (500 MHz, $(CD_3)_2SO$) of **5b**

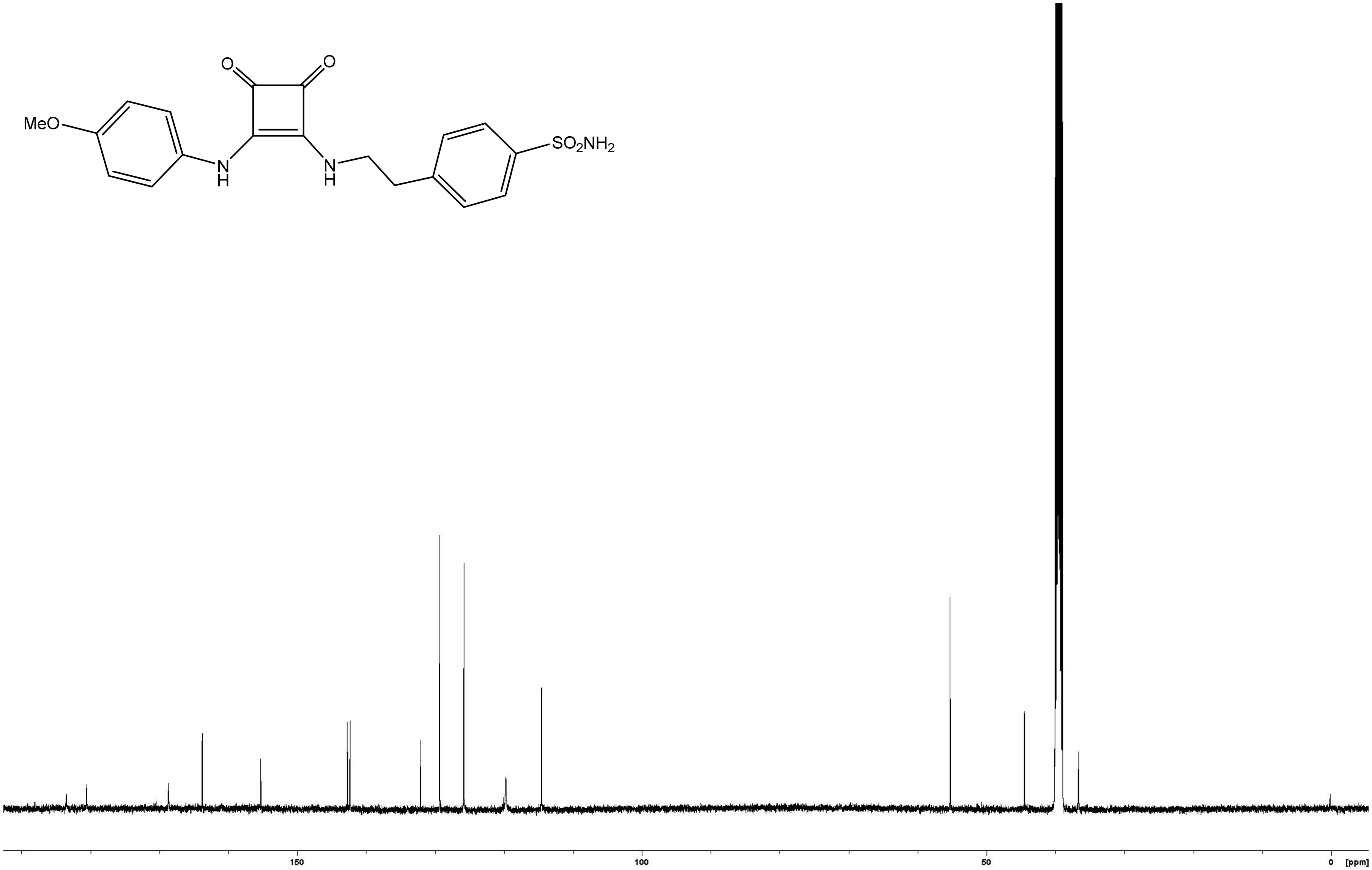

^{13}C-NMR (125.7 MHz, $(CD_3)_2SO$) of **5b**

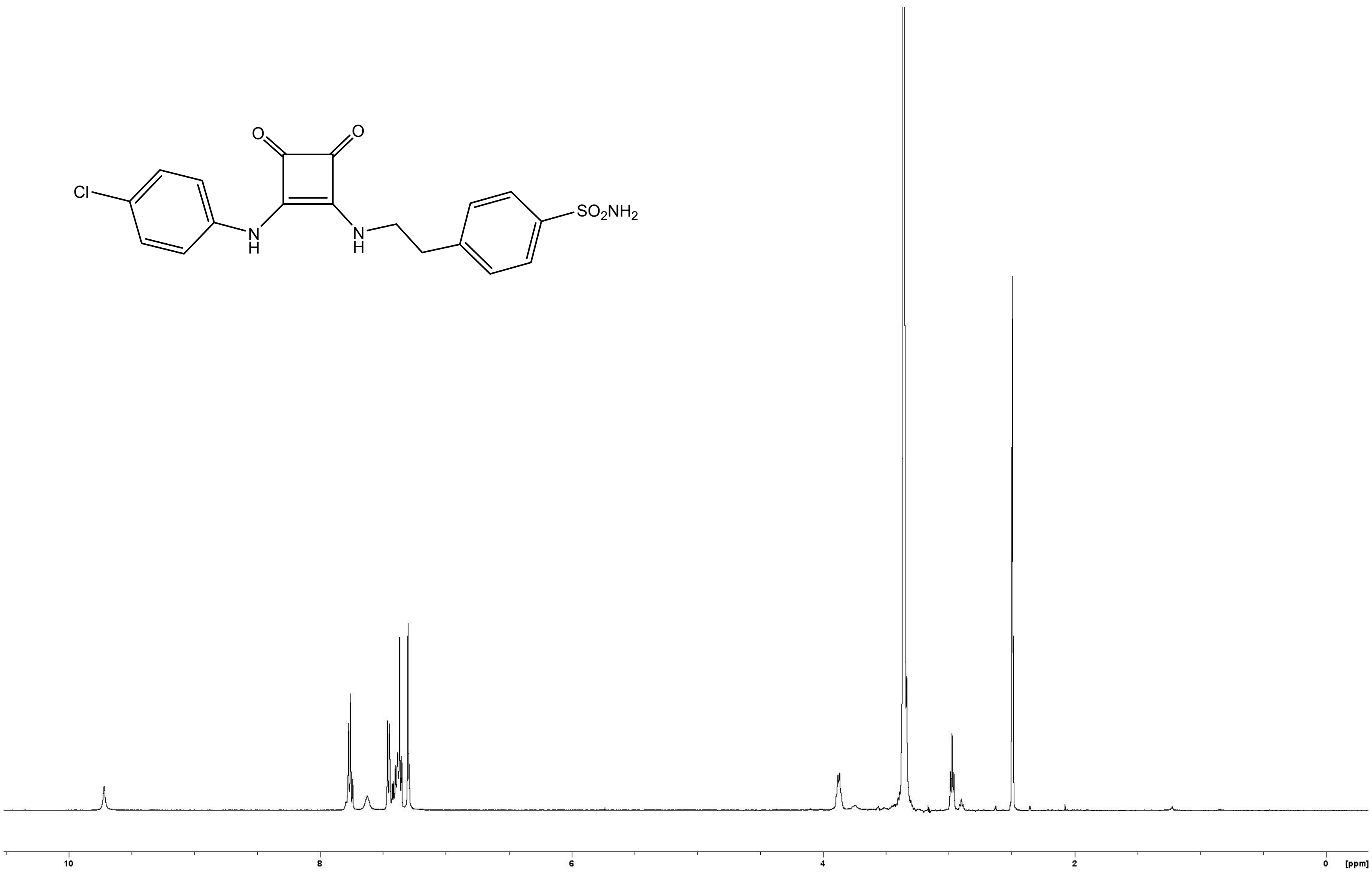

^{1}H-NMR (500 MHz, $(CD_3)_2SO$) of **5c**

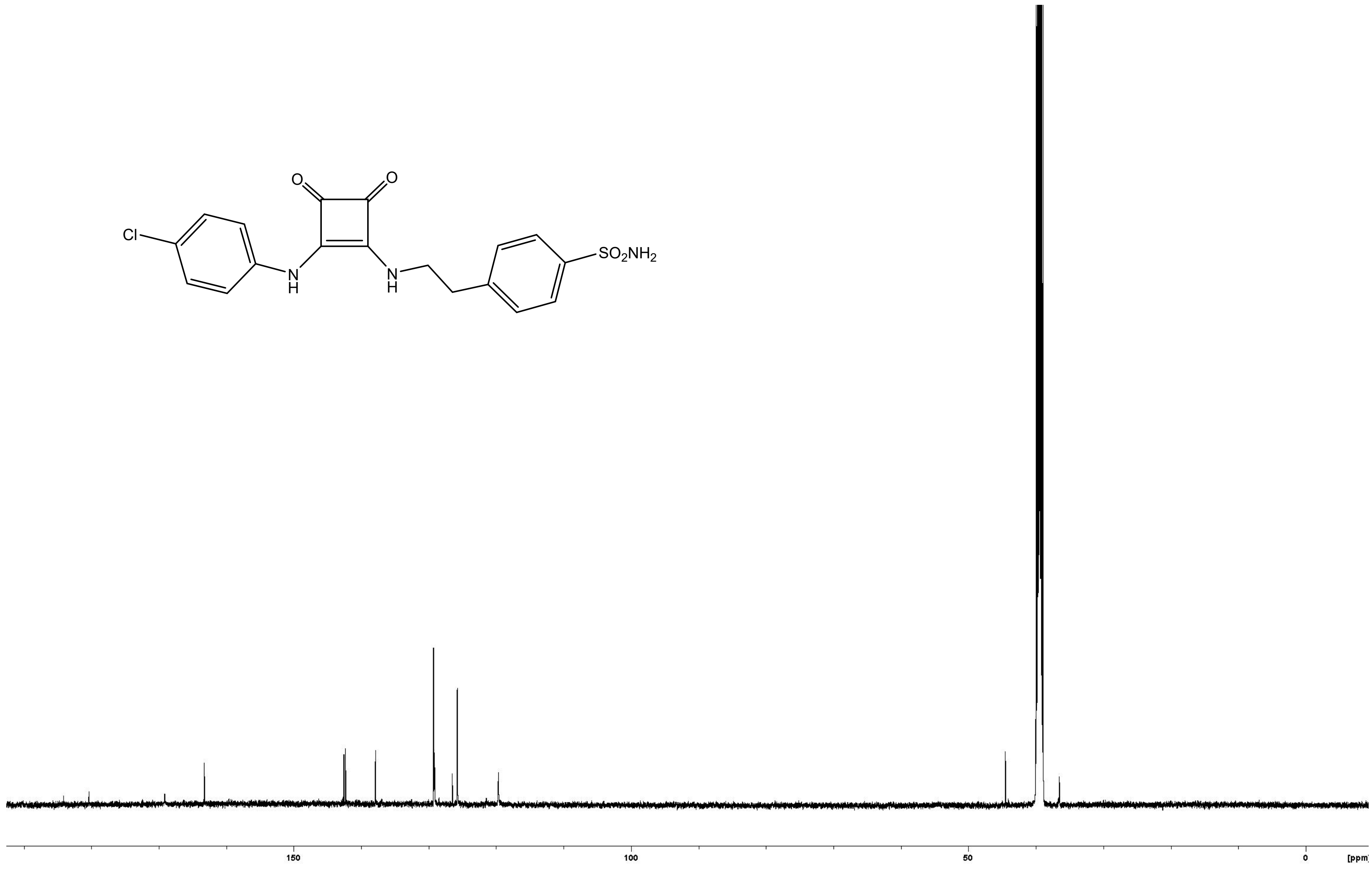

^{13}C-NMR (125.7 MHz, $(CD_3)_2SO$) of **5c**

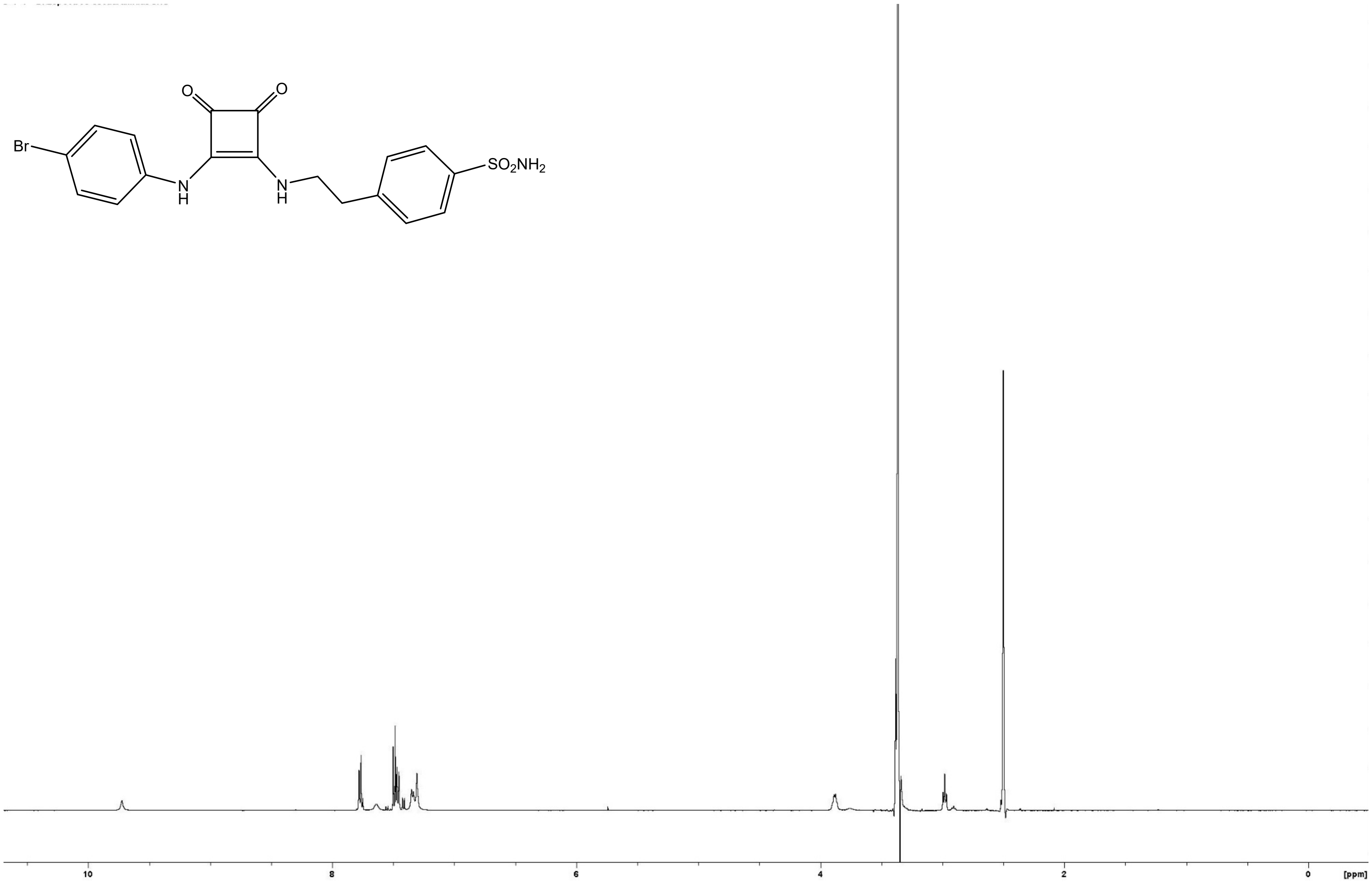

^{1}H-NMR (500 MHz, $(CD_3)_2SO$) of **5d**

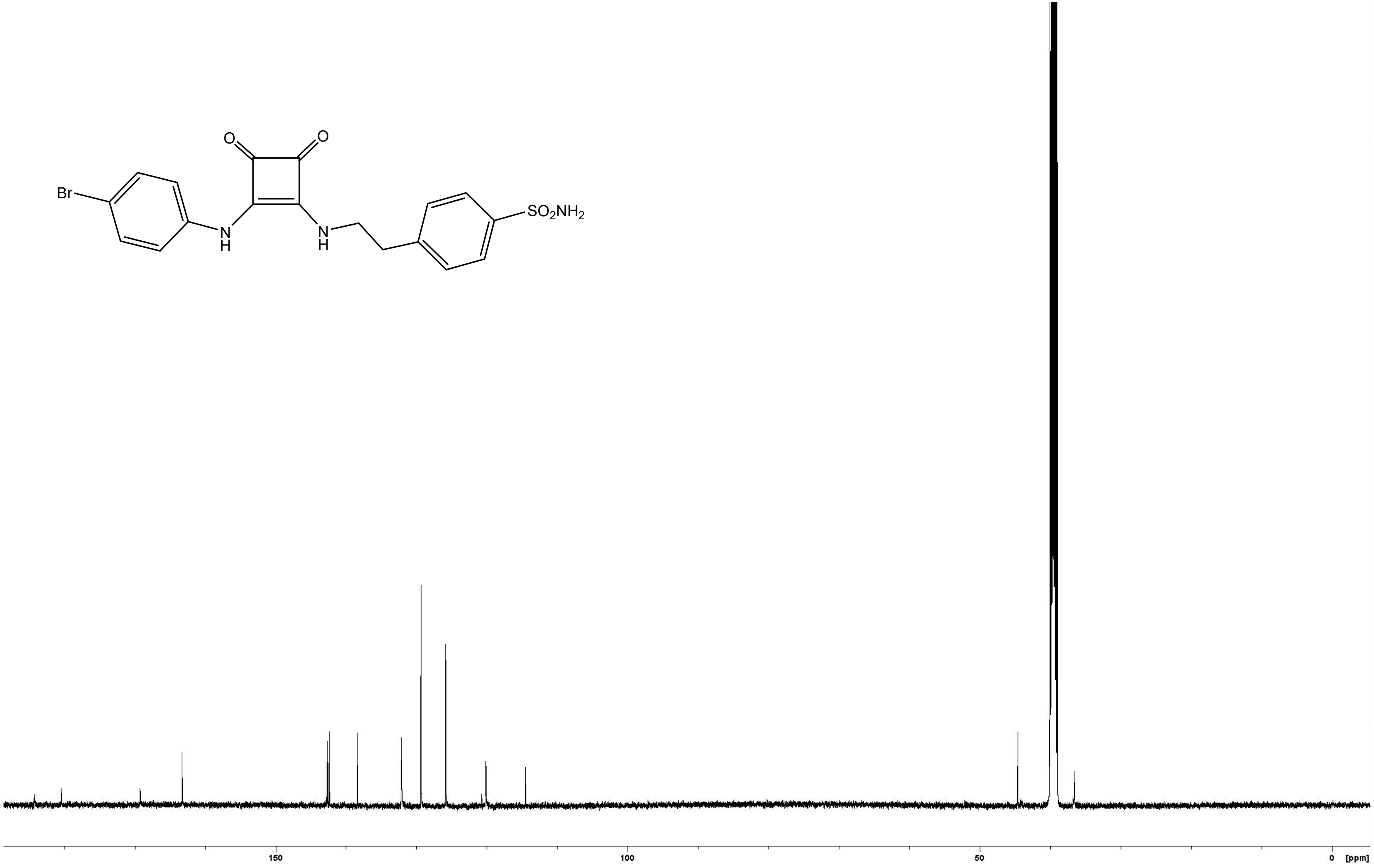

^{13}C-NMR (125.7 MHz, $(CD_3)_2SO$) of **5d**

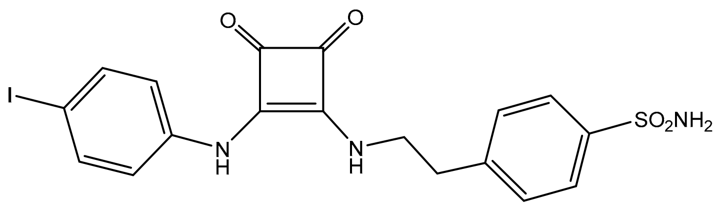

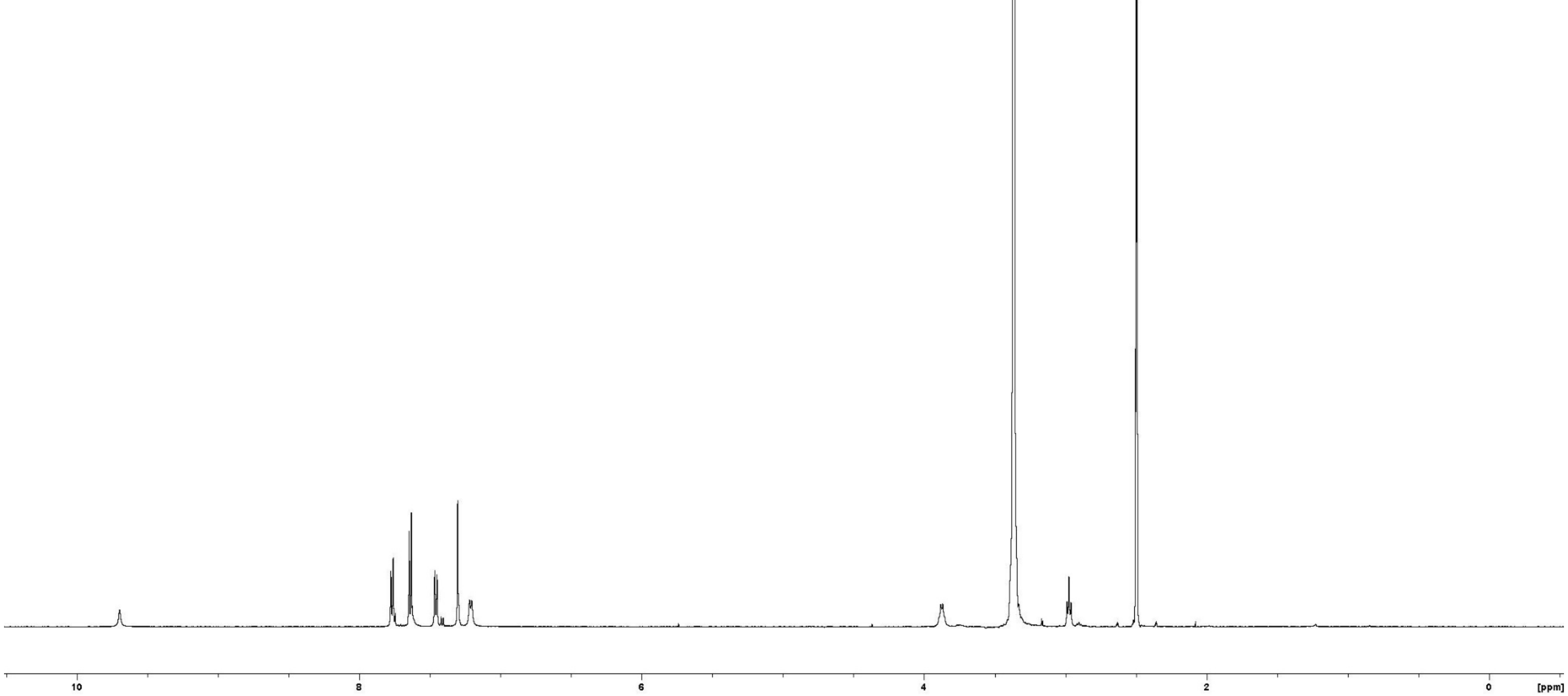

^{1}H-NMR (500 MHz, $(CD_3)_2SO$) of **5e**

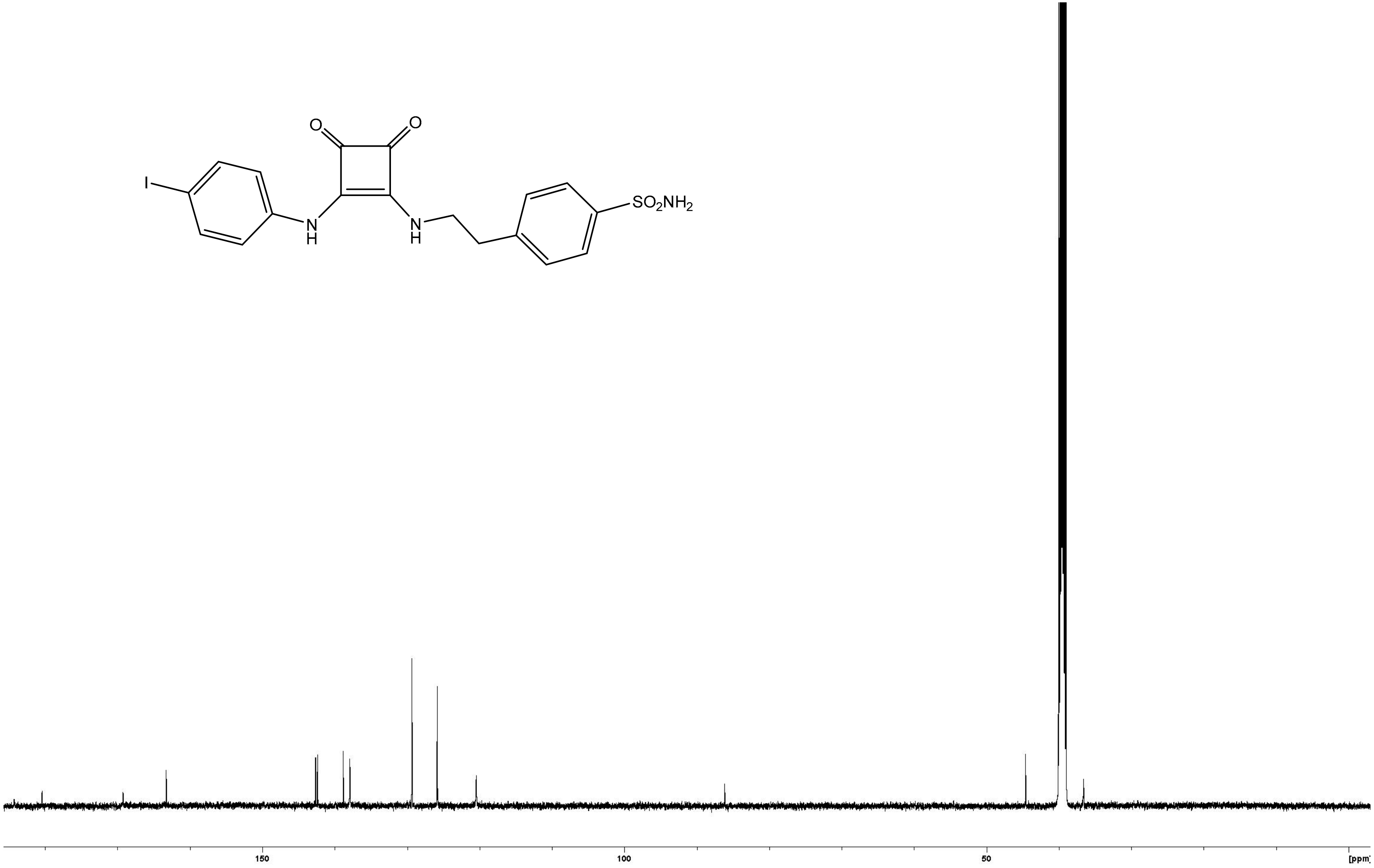

^{13}C-NMR (125.7 MHz, $(CD_3)_2SO$) of **5e**

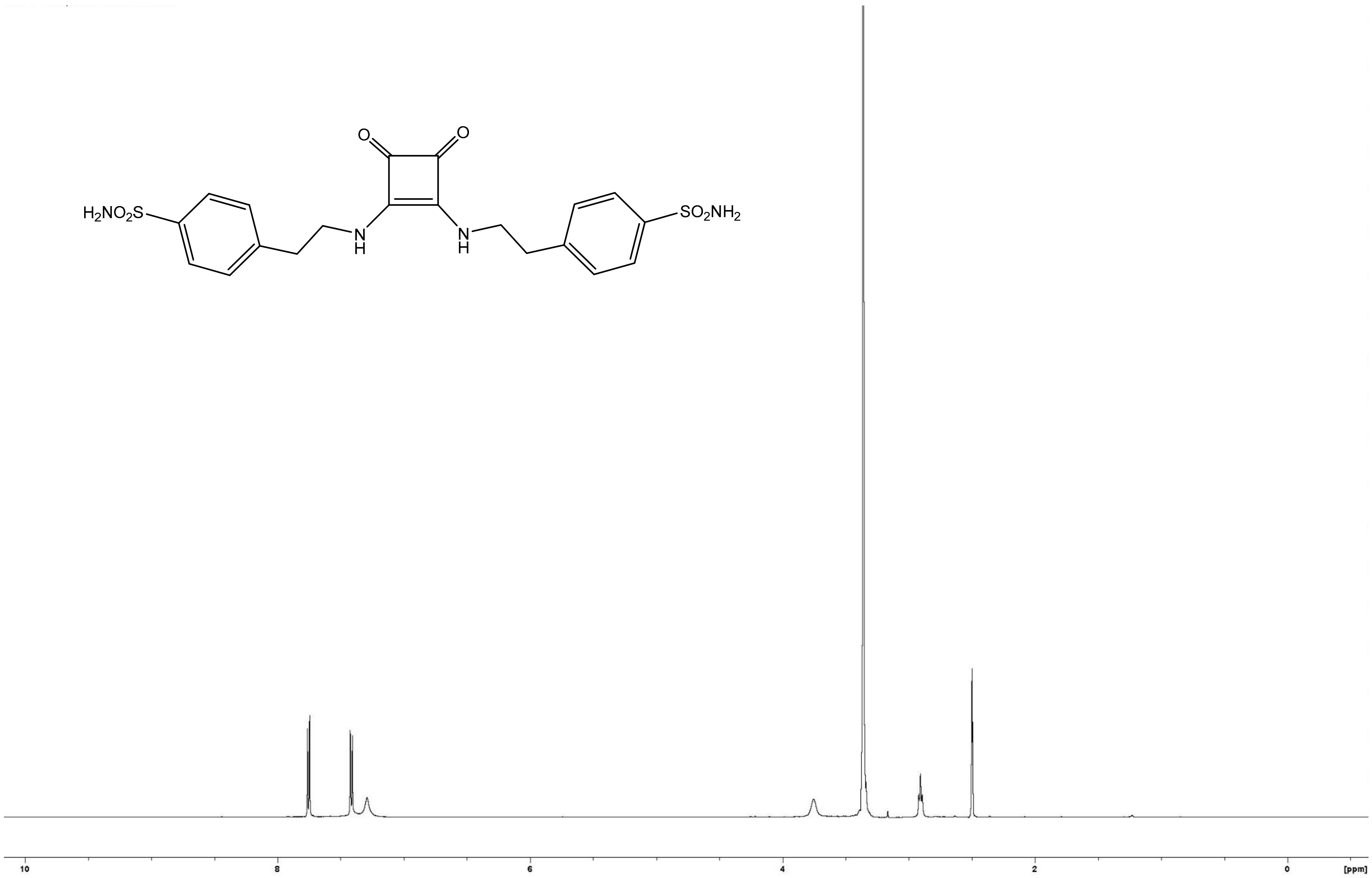

^{1}H-NMR (500 MHz, $(CD_3)_2SO$) of **6**

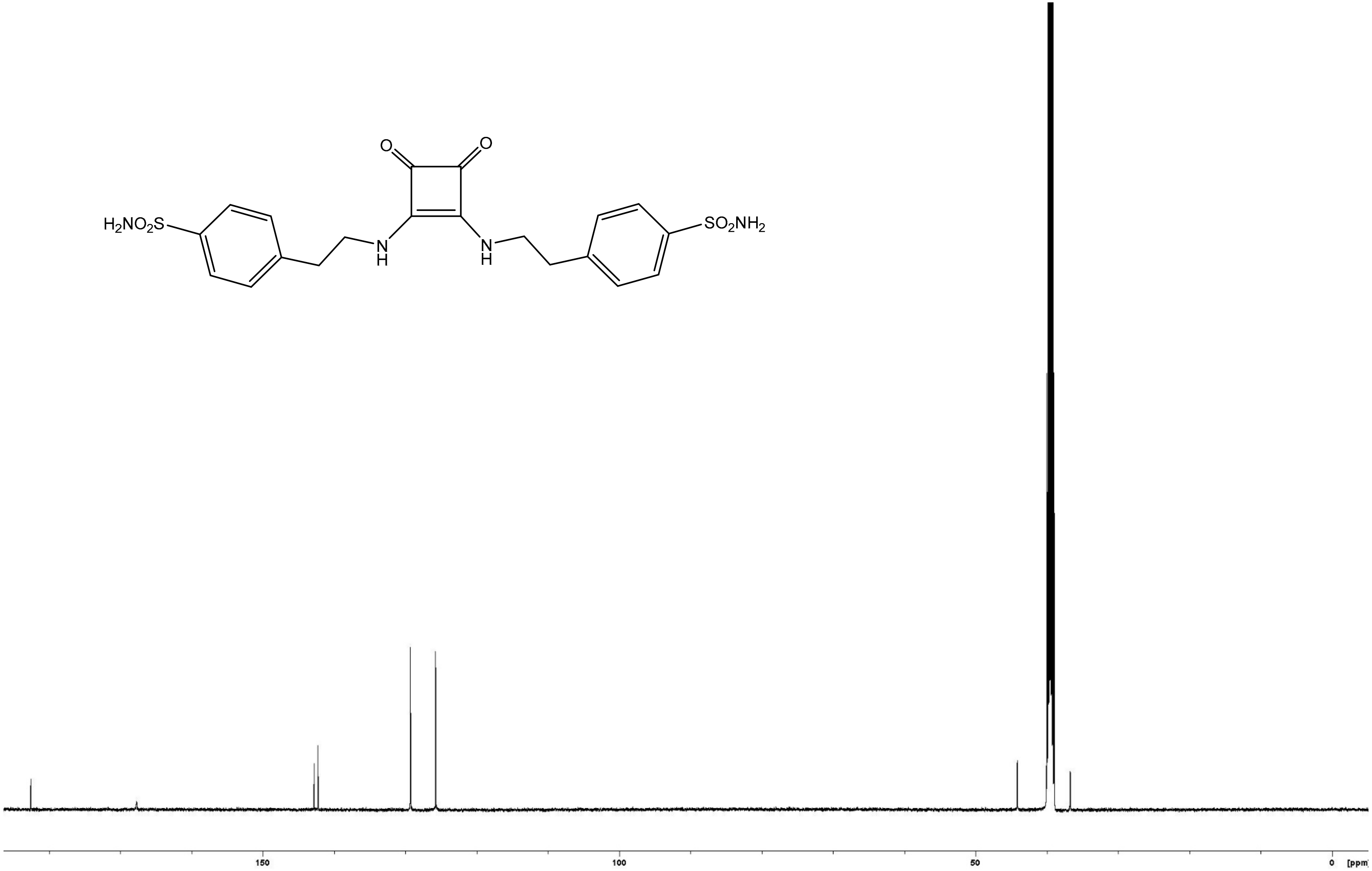

^{13}C-NMR (125.7 MHz, $(CD_3)_2SO$) of **6**

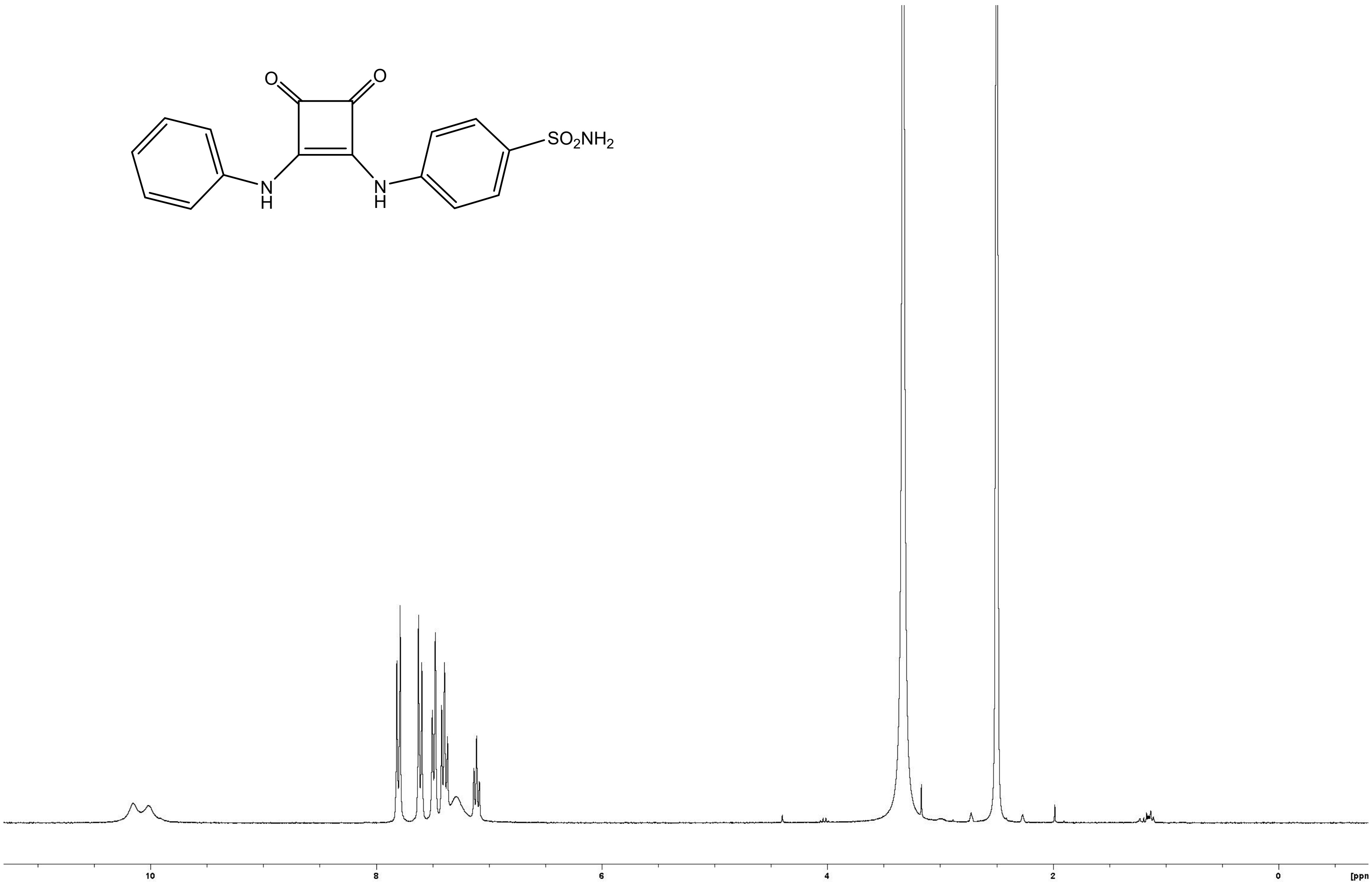

^{1}H-NMR (300 MHz, $(CD_3)_2SO$) of **8**

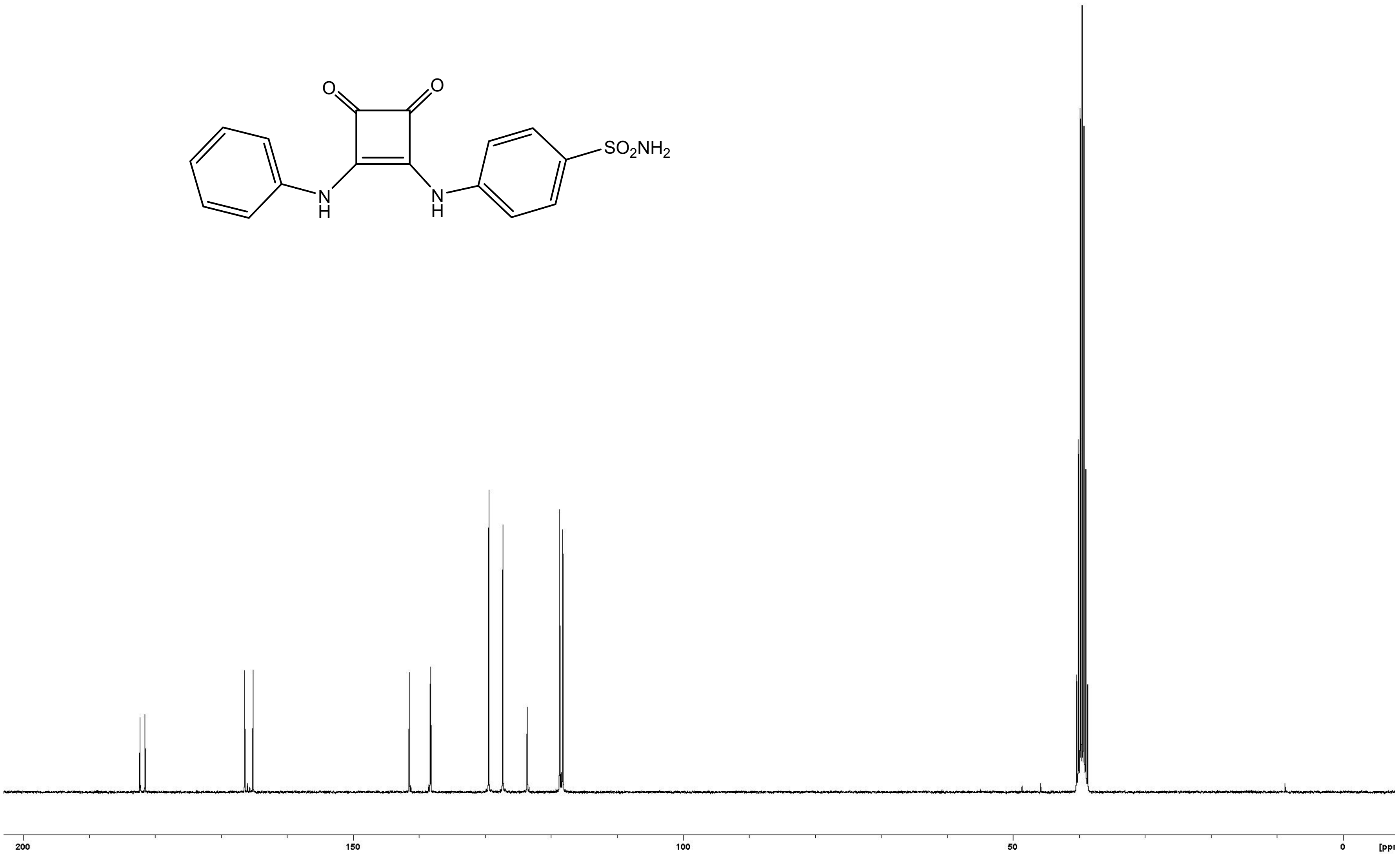

^{13}C-NMR (125.7 MHz, $(CD_3)_2SO$) of **8**

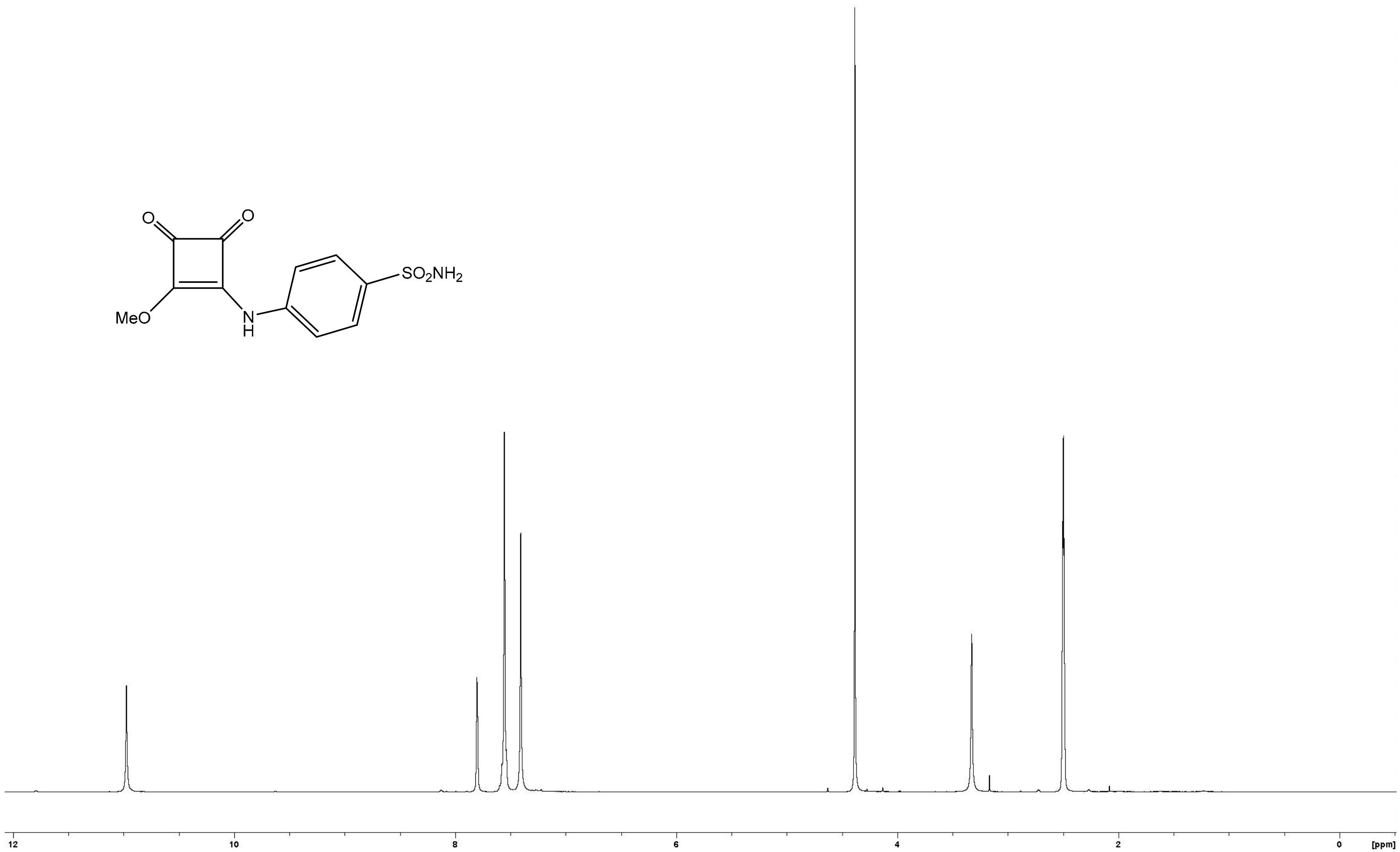

^{1}H-NMR (300 MHz, $(CD_3)_2SO$) of **10**

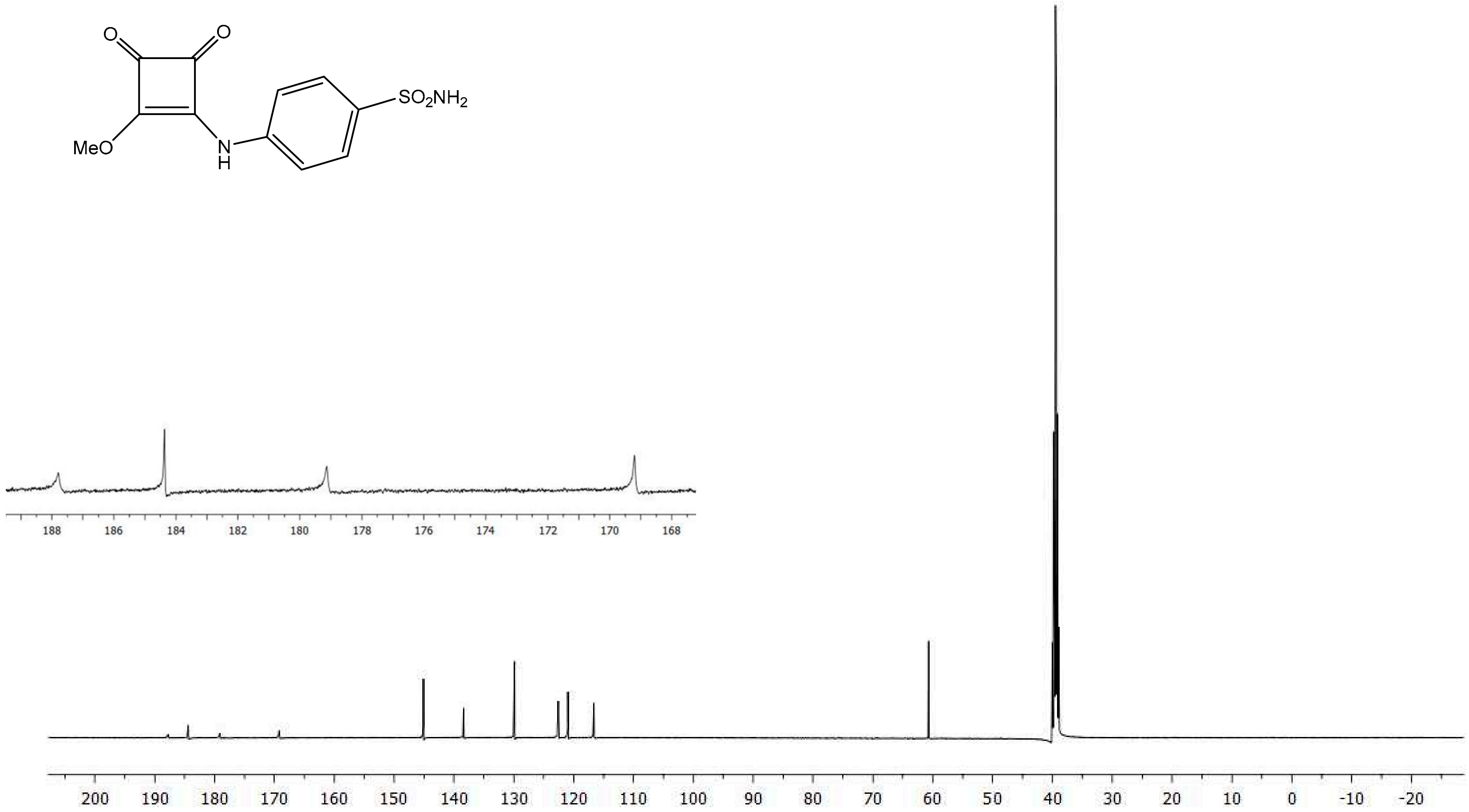

^{13}C-NMR (125.7 MHz, $(CD_3)_2SO$) of **10**

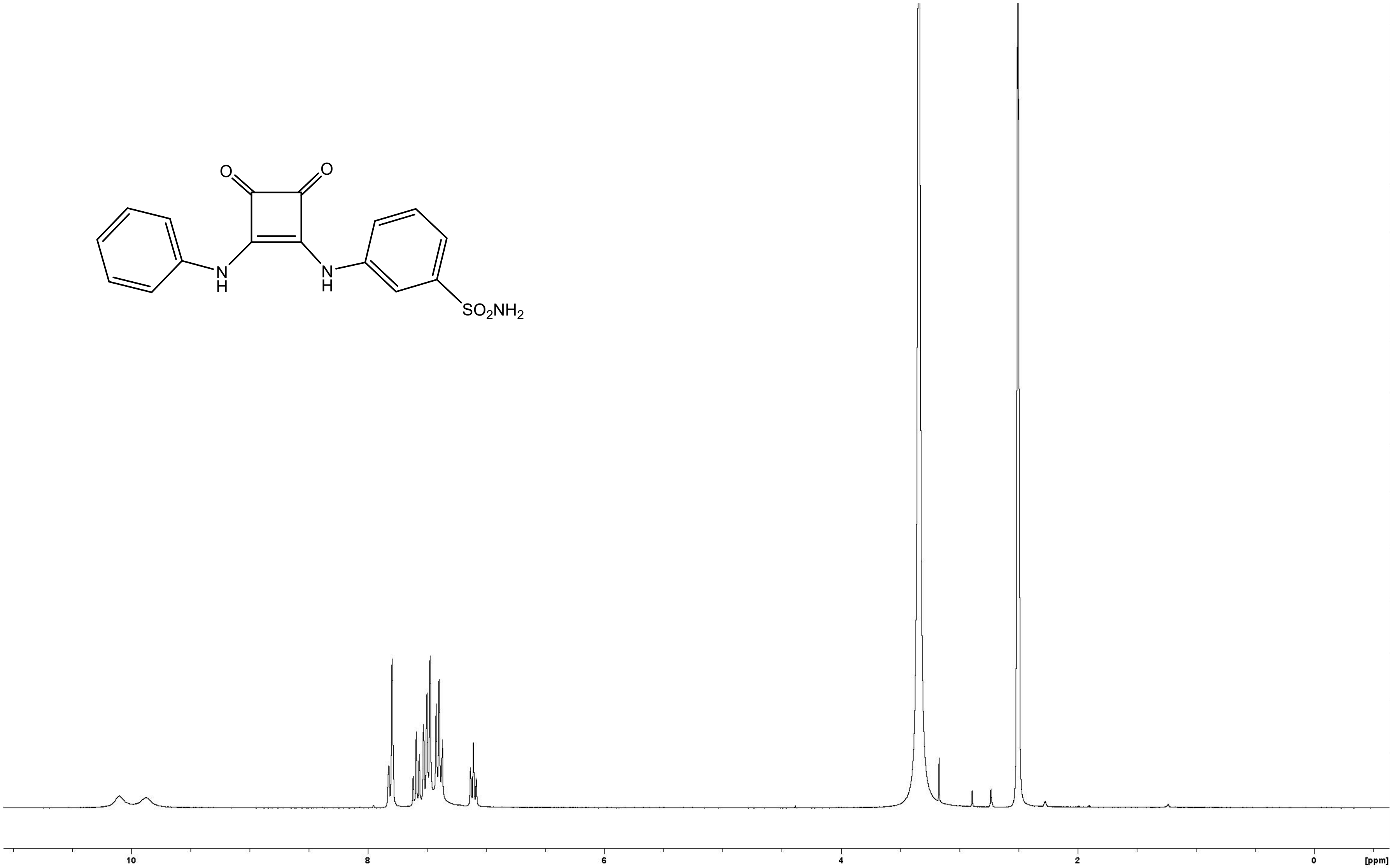

^{1}H-NMR (300 MHz, $(CD_3)_2SO$) of **11**

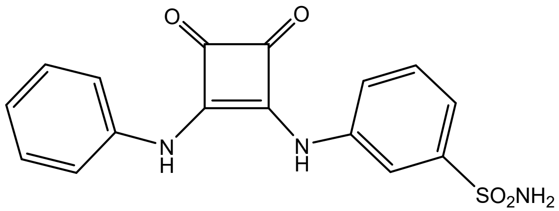

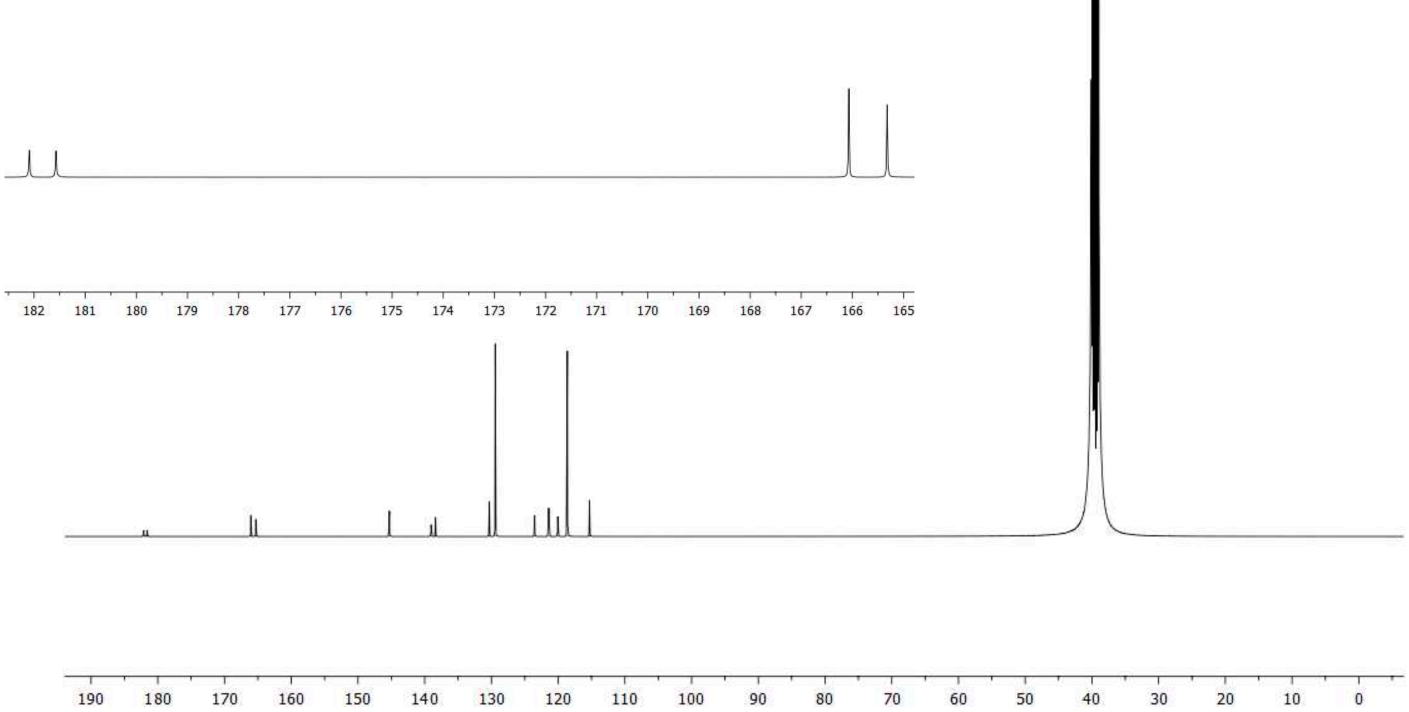

^{13}C-NMR (300 MHz, $(CD_3)_2SO$) of **11**

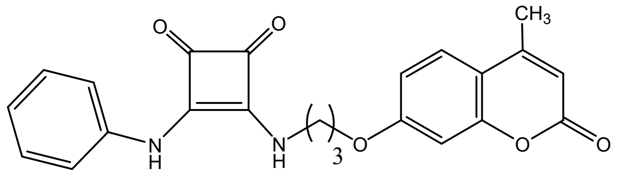

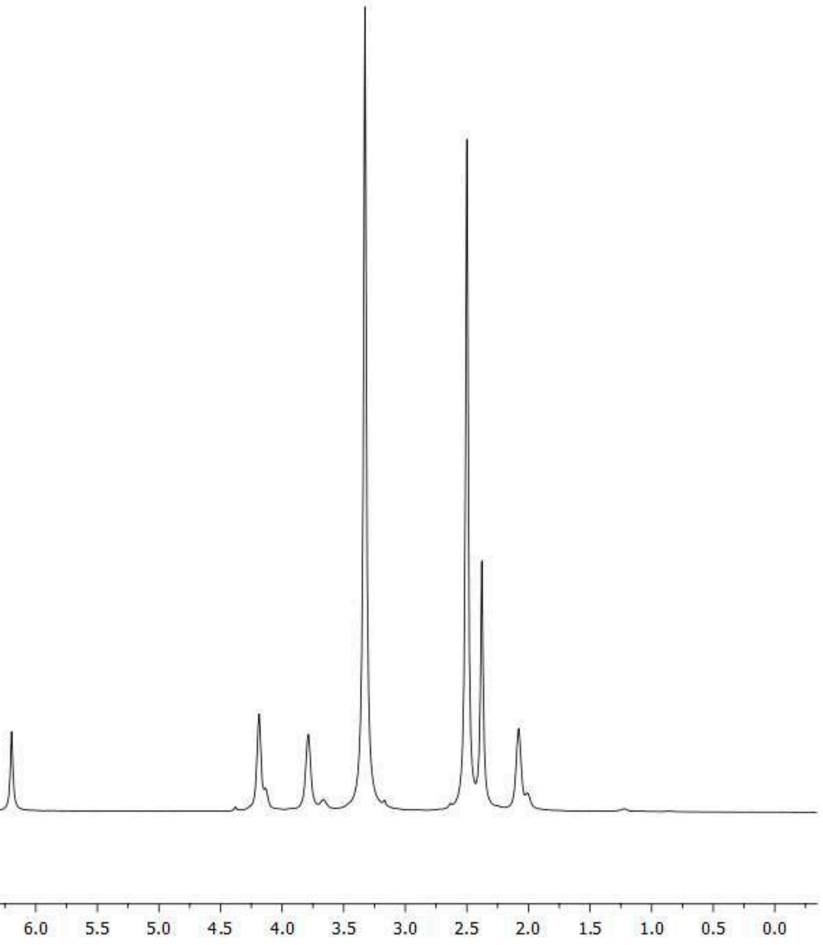

^{1}H-NMR (500 MHz, $(CD_3)_2SO$) of **16a**

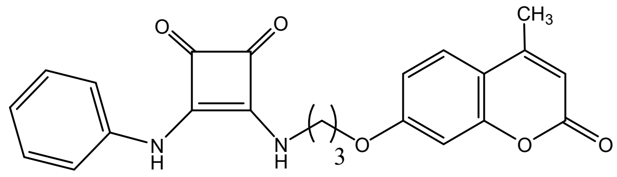

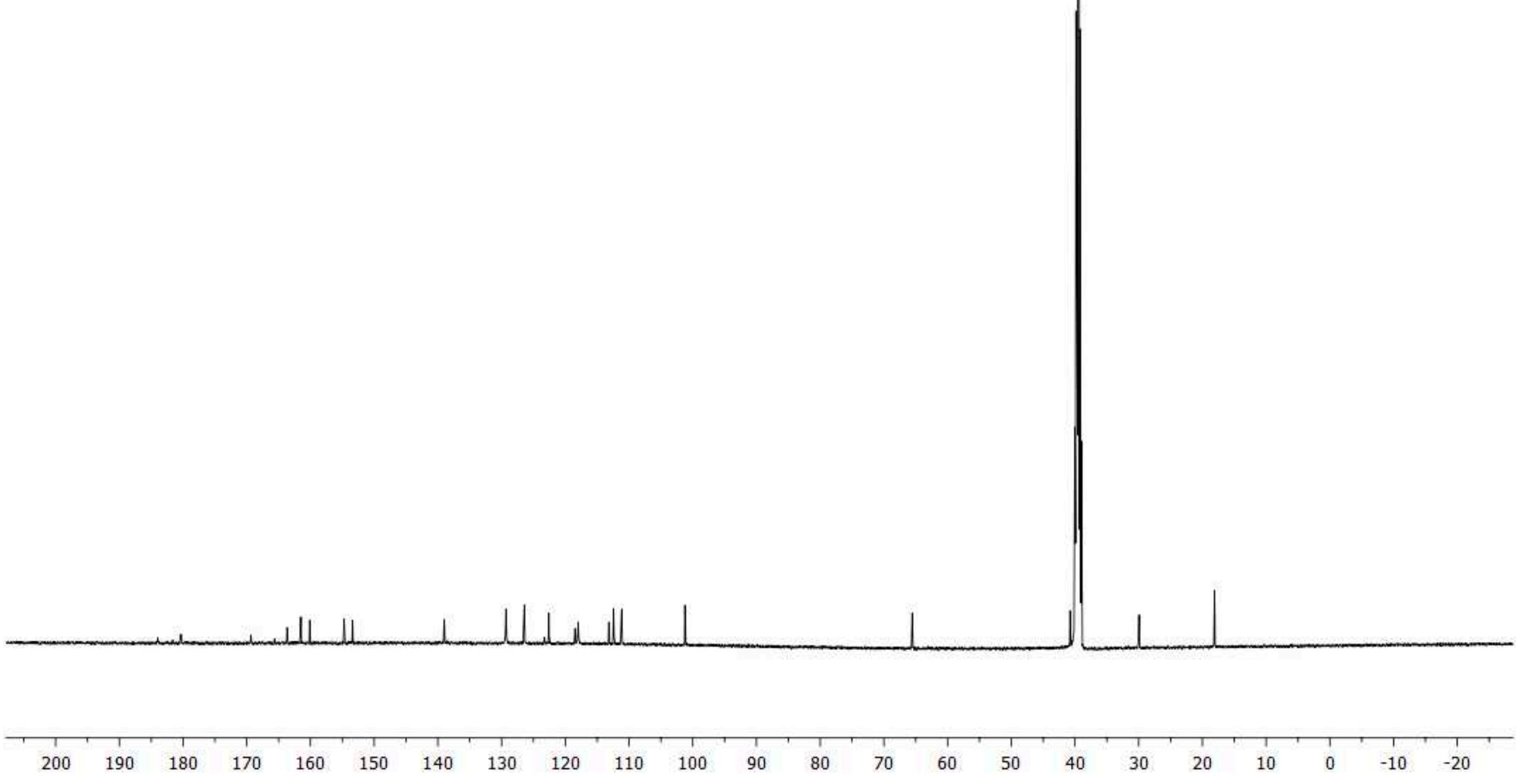

^{13}C-NMR (125.7 MHz, $(CD_3)_2SO$) of **16a**

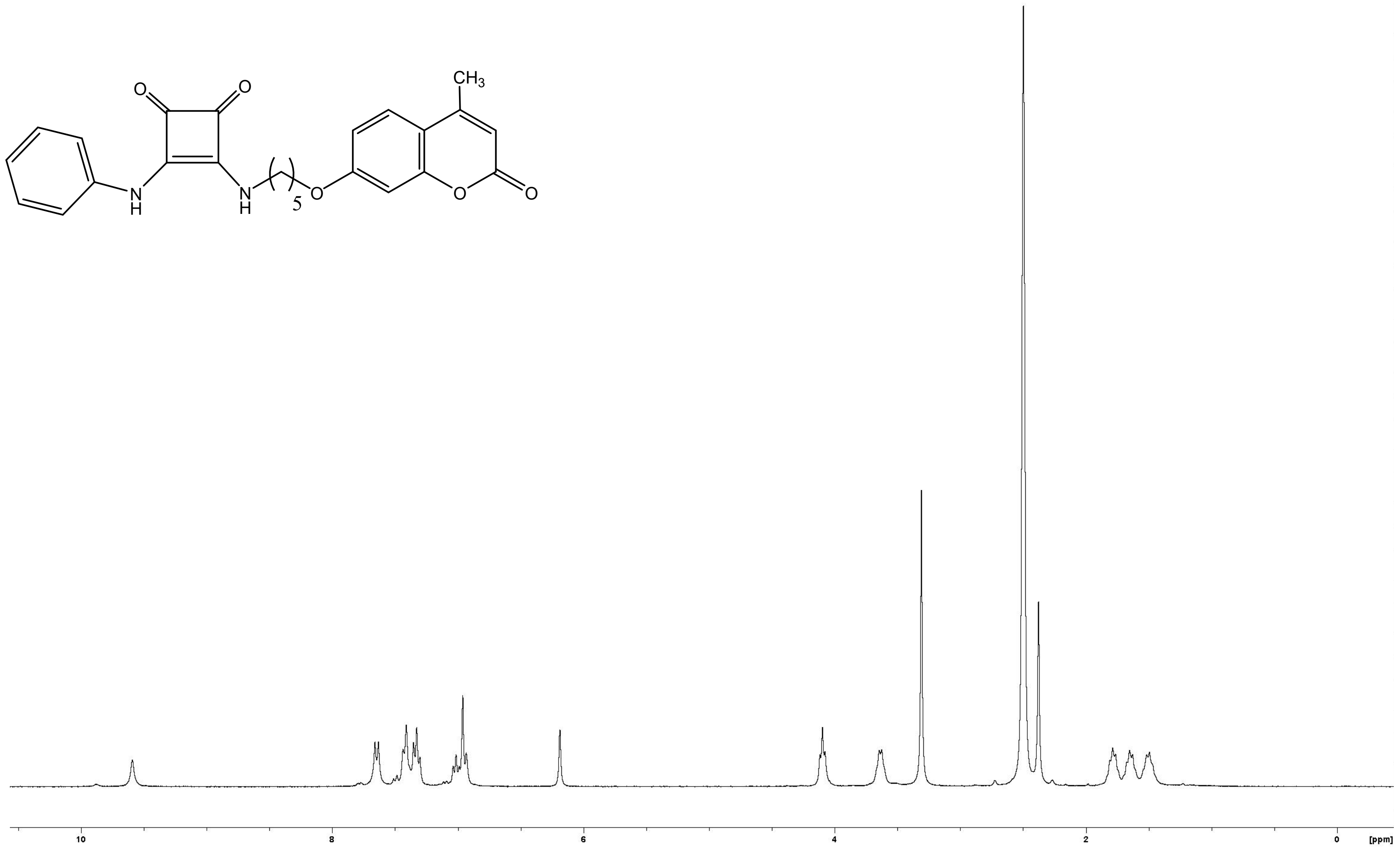

^{1}H-NMR (300 MHz, $(CD_3)_2SO$) of **16b**

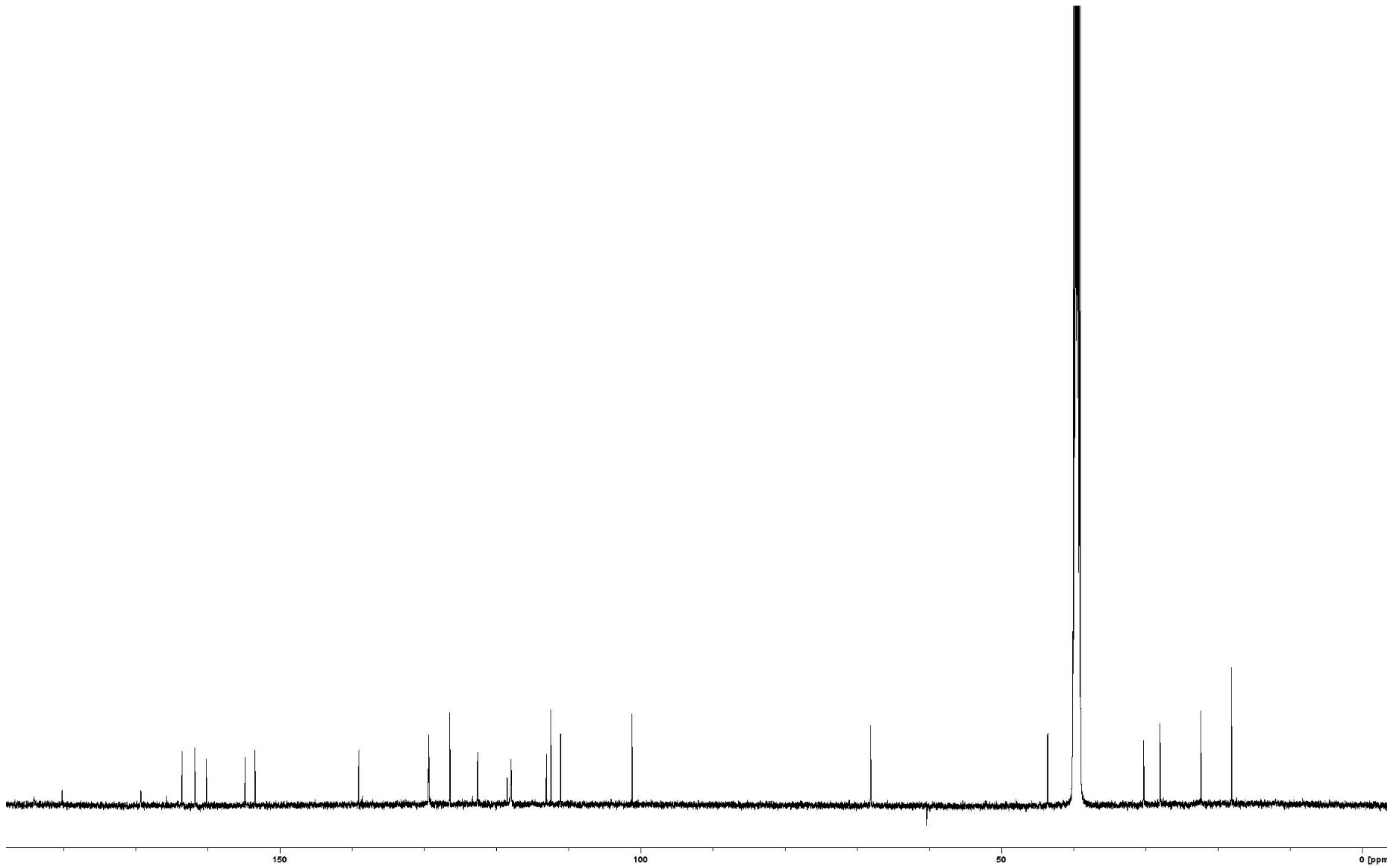

^{13}C-NMR (125.7 MHz, $(CD_3)_2SO$) of **16b**

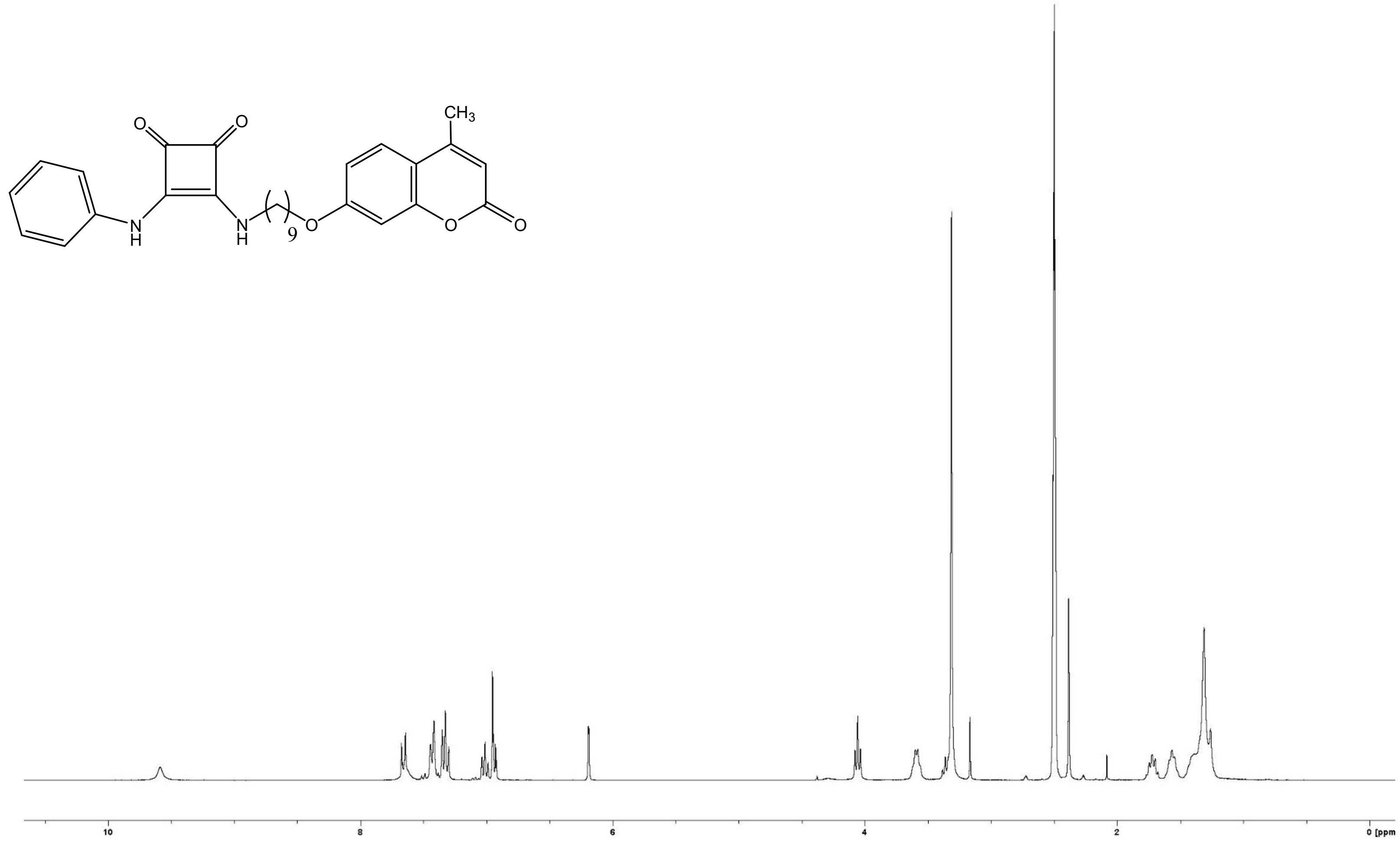

^{1}H-NMR (300 MHz, $(CD_3)_2SO$) of **16c**

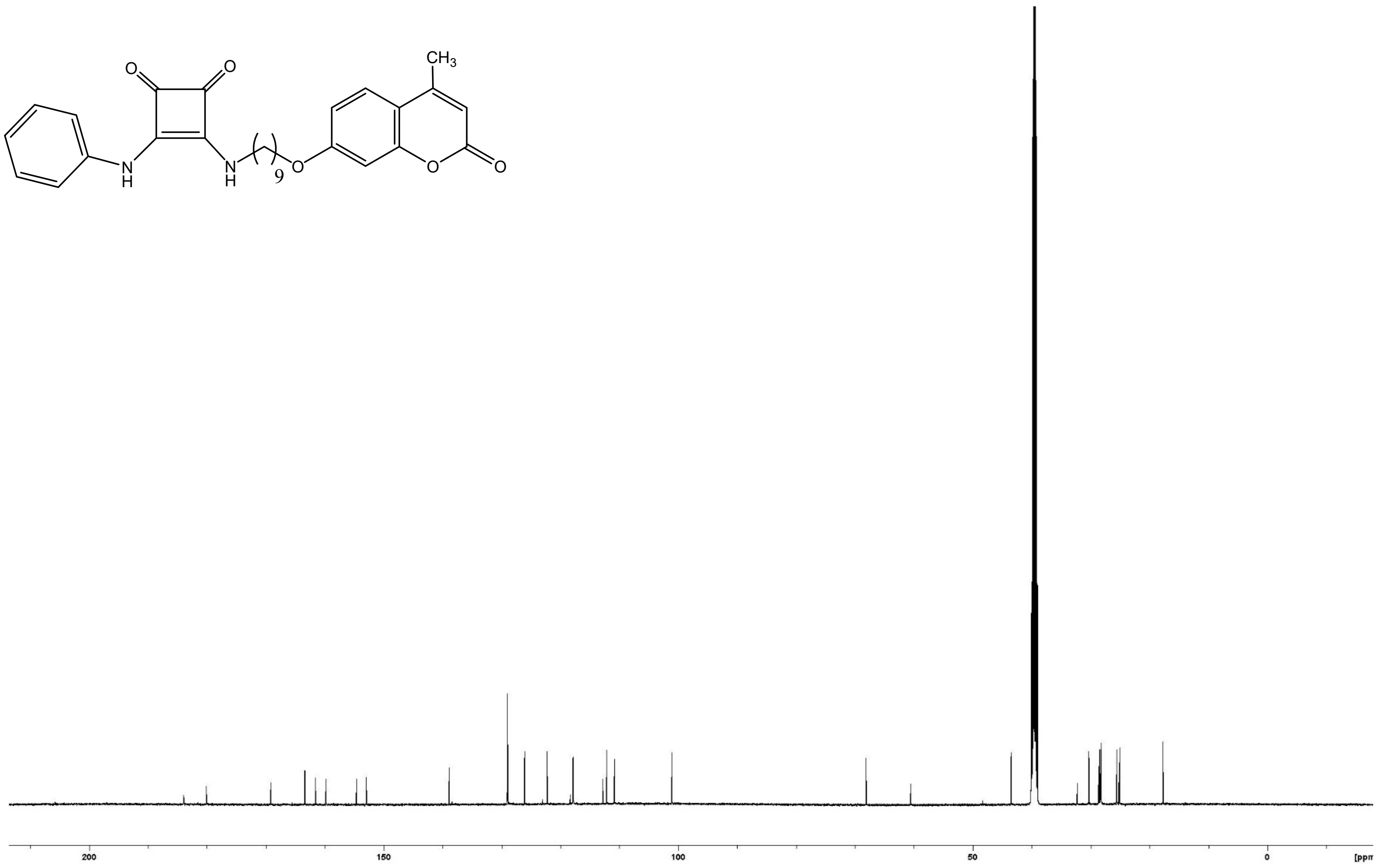

^{13}C-NMR (125.7 MHz, $(CD_3)_2SO$) of **16c**

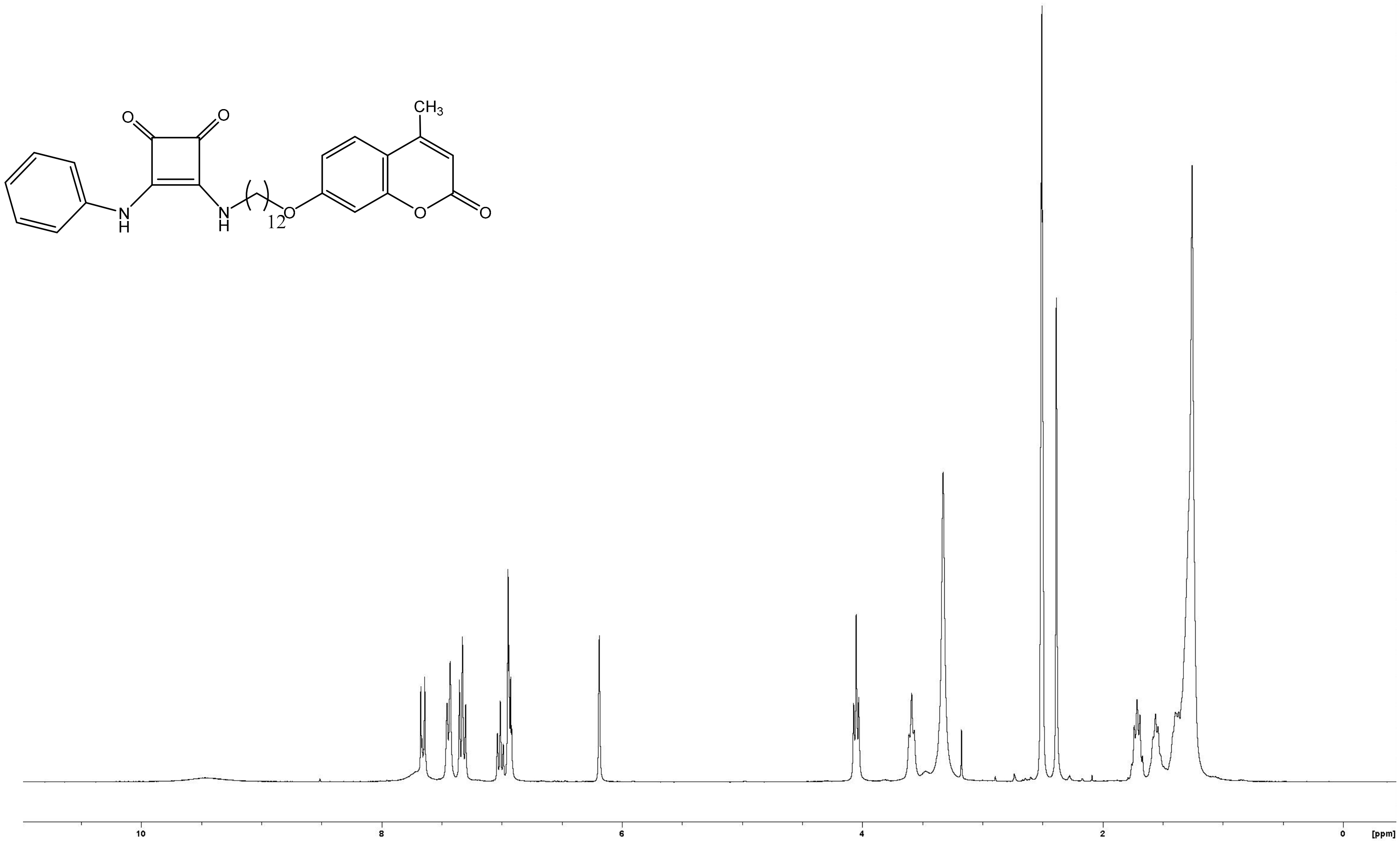

^{1}H-NMR (300 MHz, $(CD_3)_2SO$) of **16d**

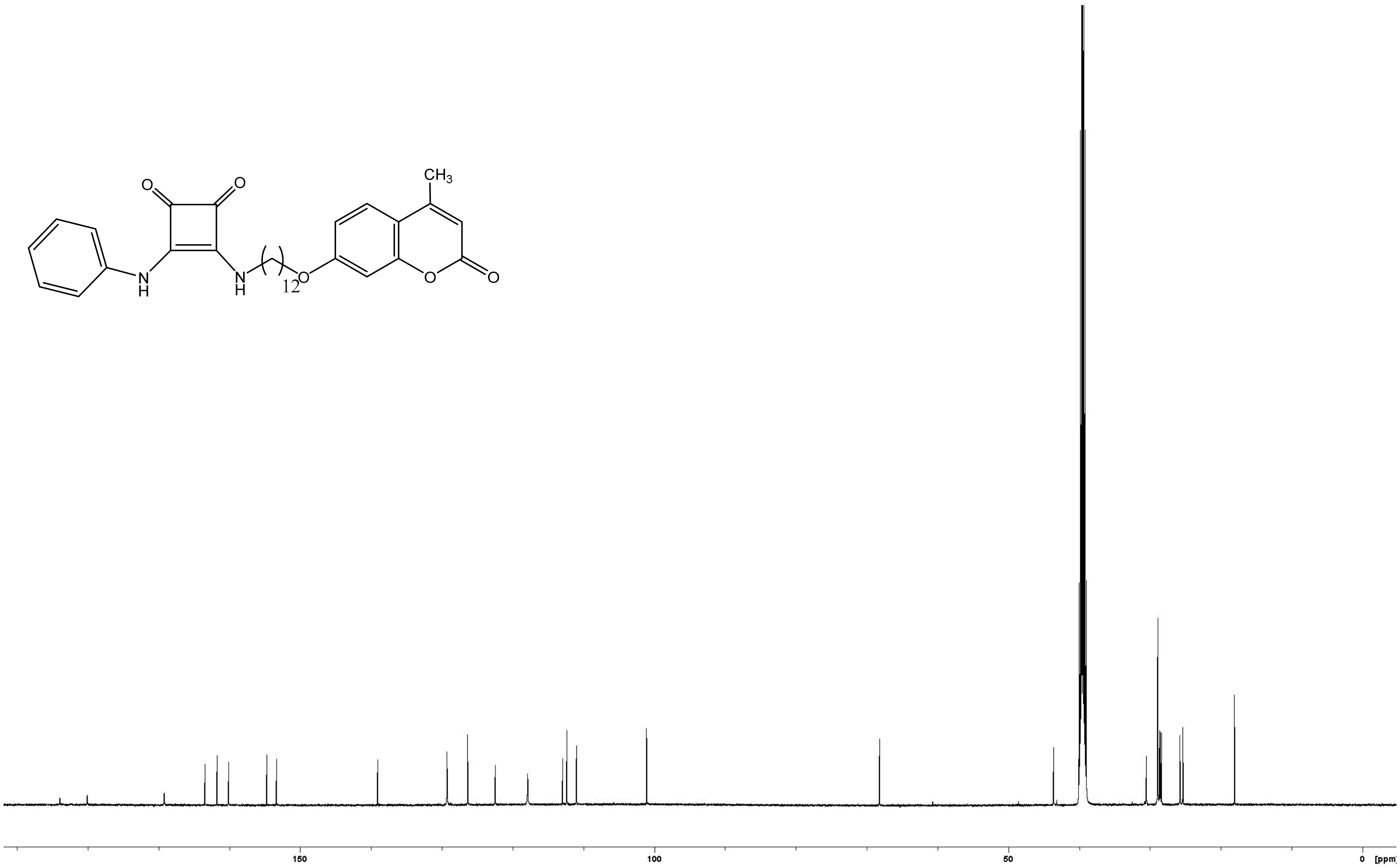

^{13}C-NMR (125.7 MHz, $(CD_3)_2SO$) of **16d**

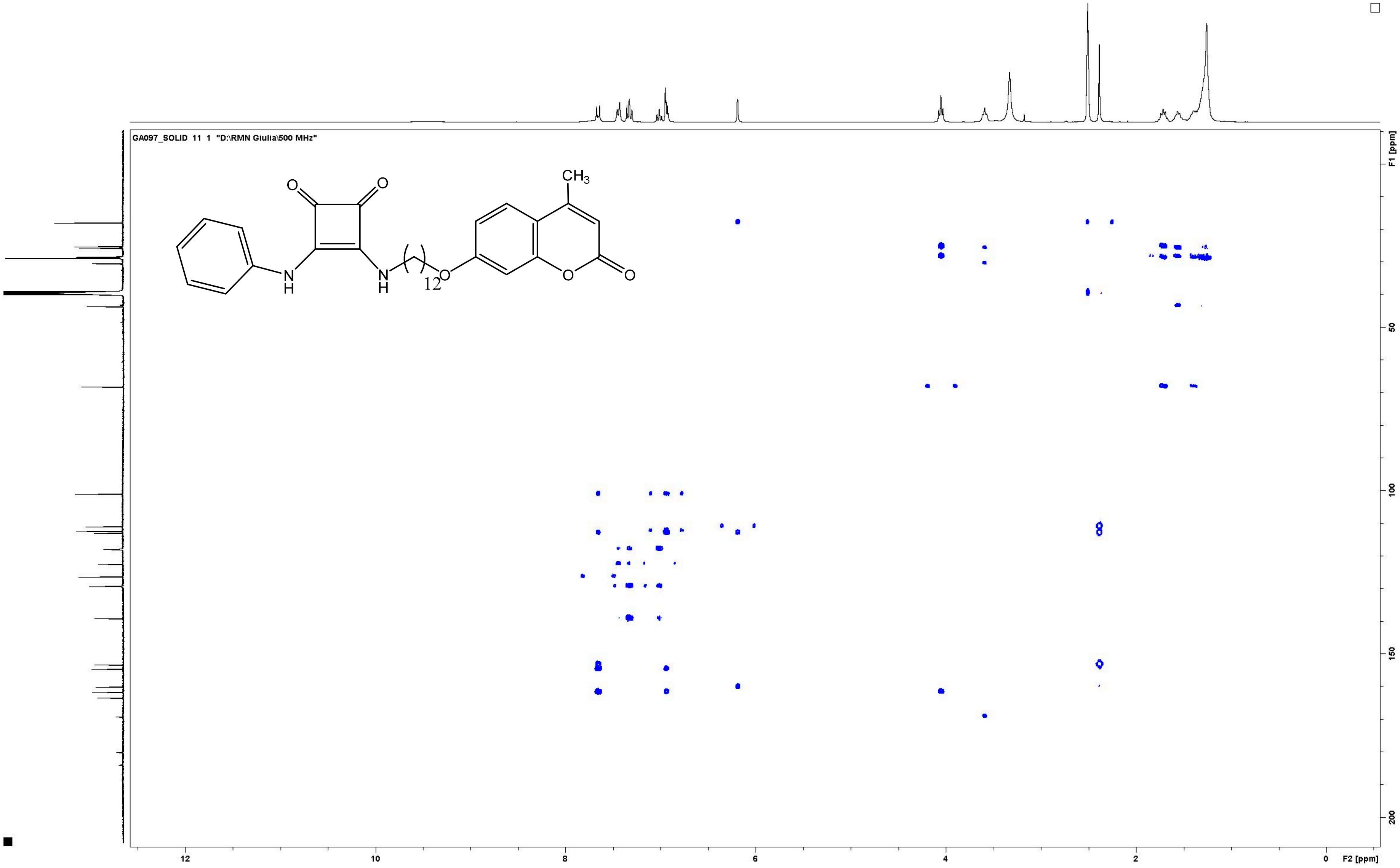

HMBC ^{1}H-^{13}C (500 MHz, $(CD_3)_2SO$) of **16d**

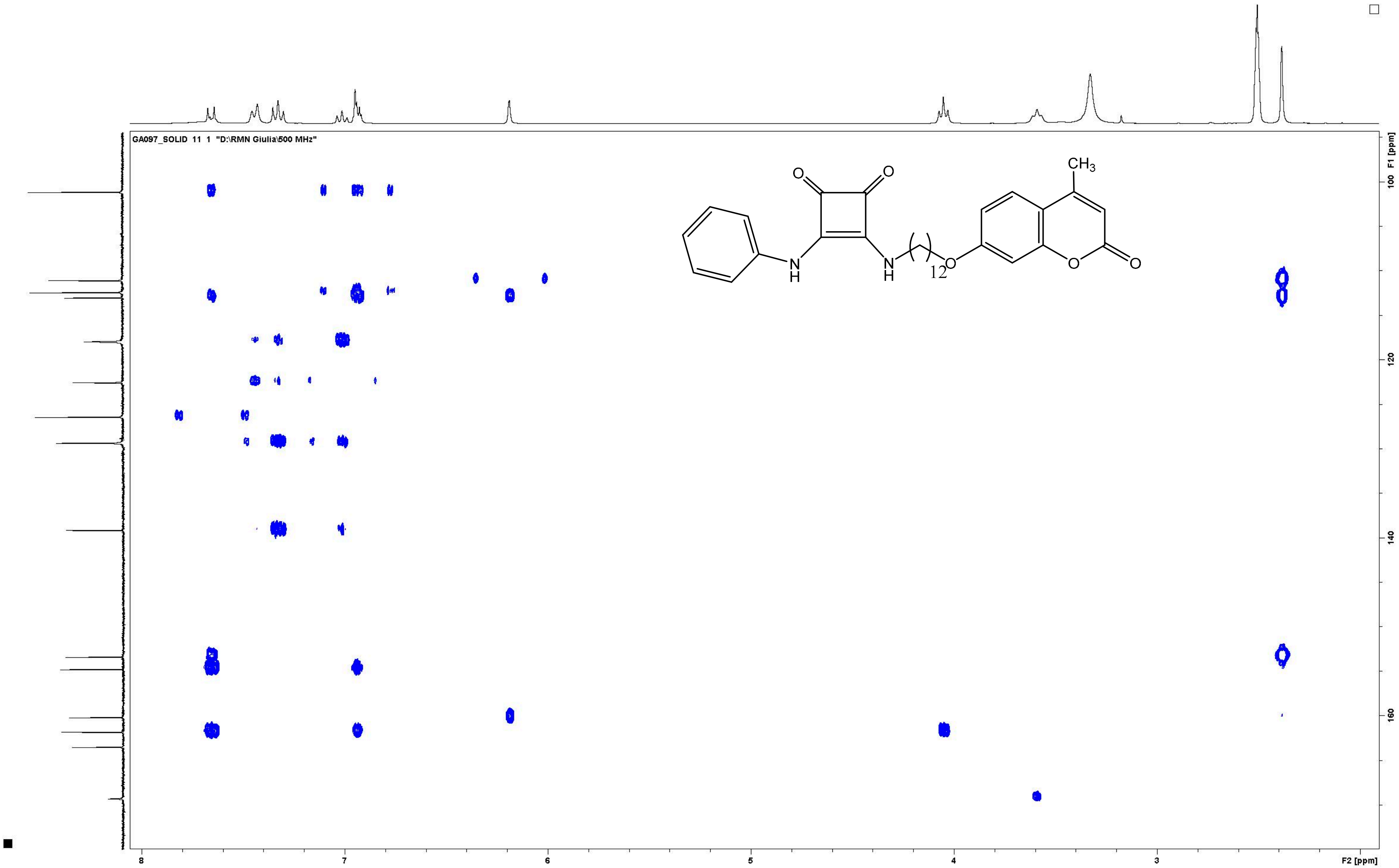

HMBC ^{1}H-^{13}C (500 MHz, $(CD_3)_2SO$) of **16d** (expansion)

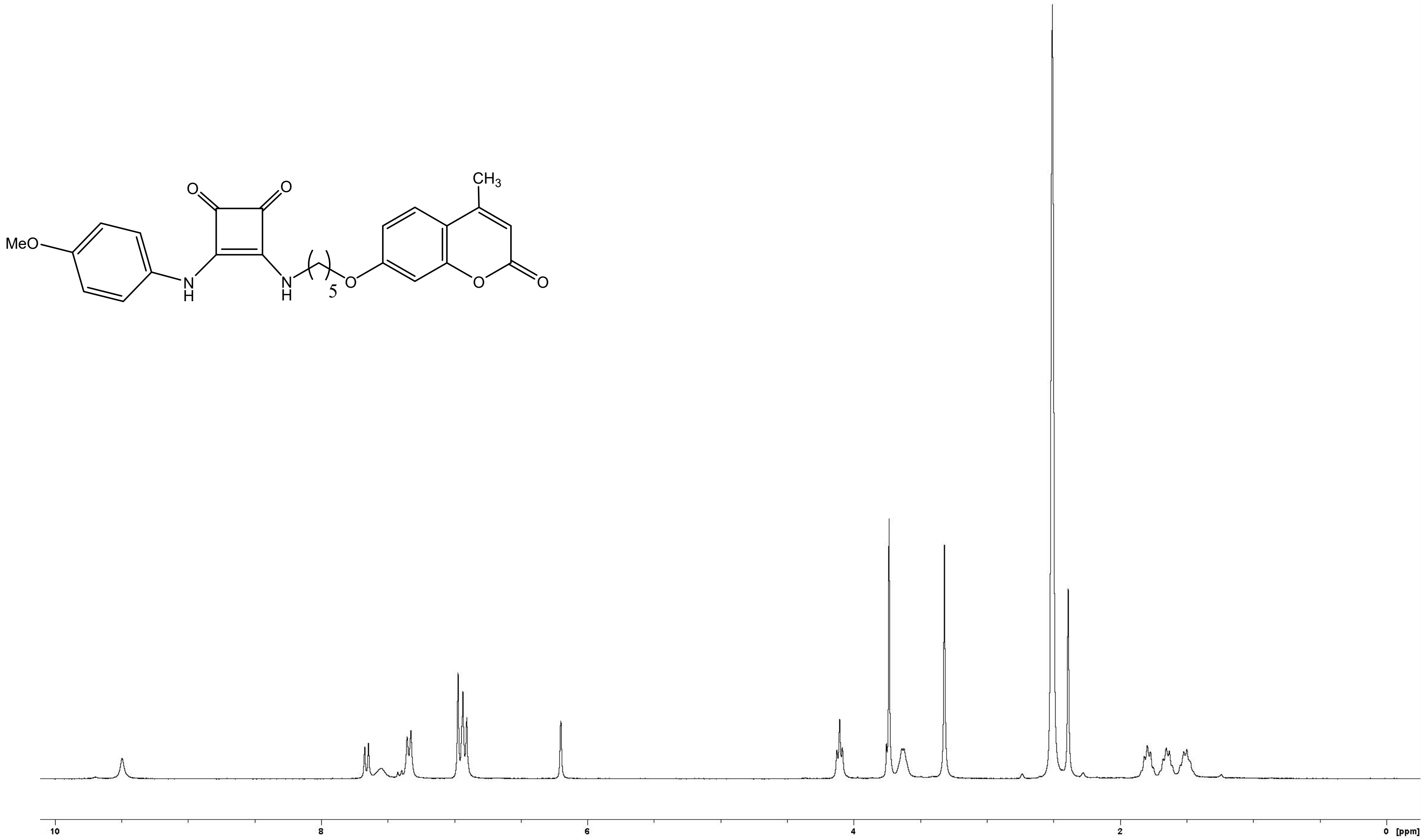

^{1}H-NMR (300 MHz, $(CD_3)_2SO$) of **16e**

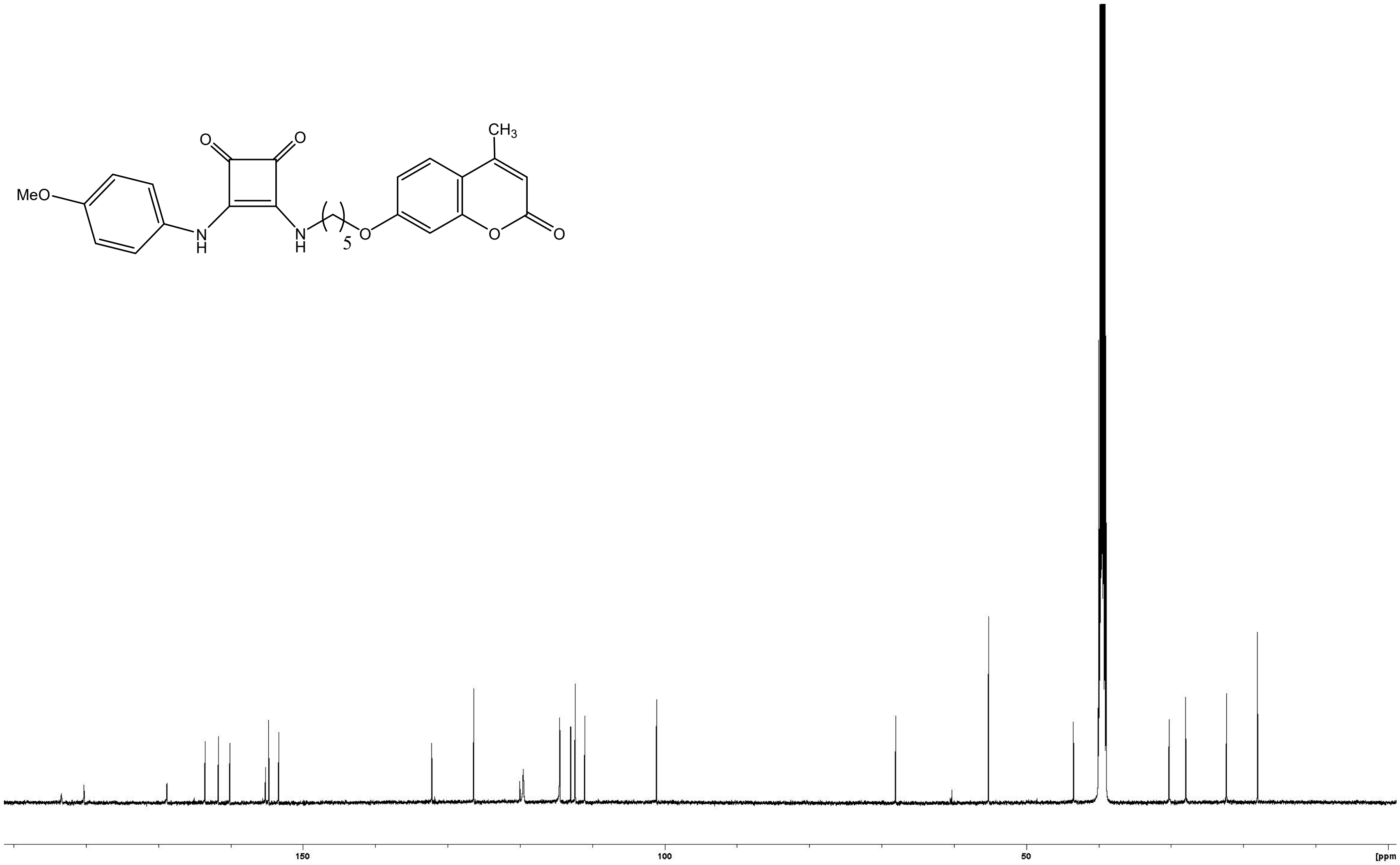

^{13}C-NMR (125.7 MHz, $(CD_3)_2SO$) of **16e**

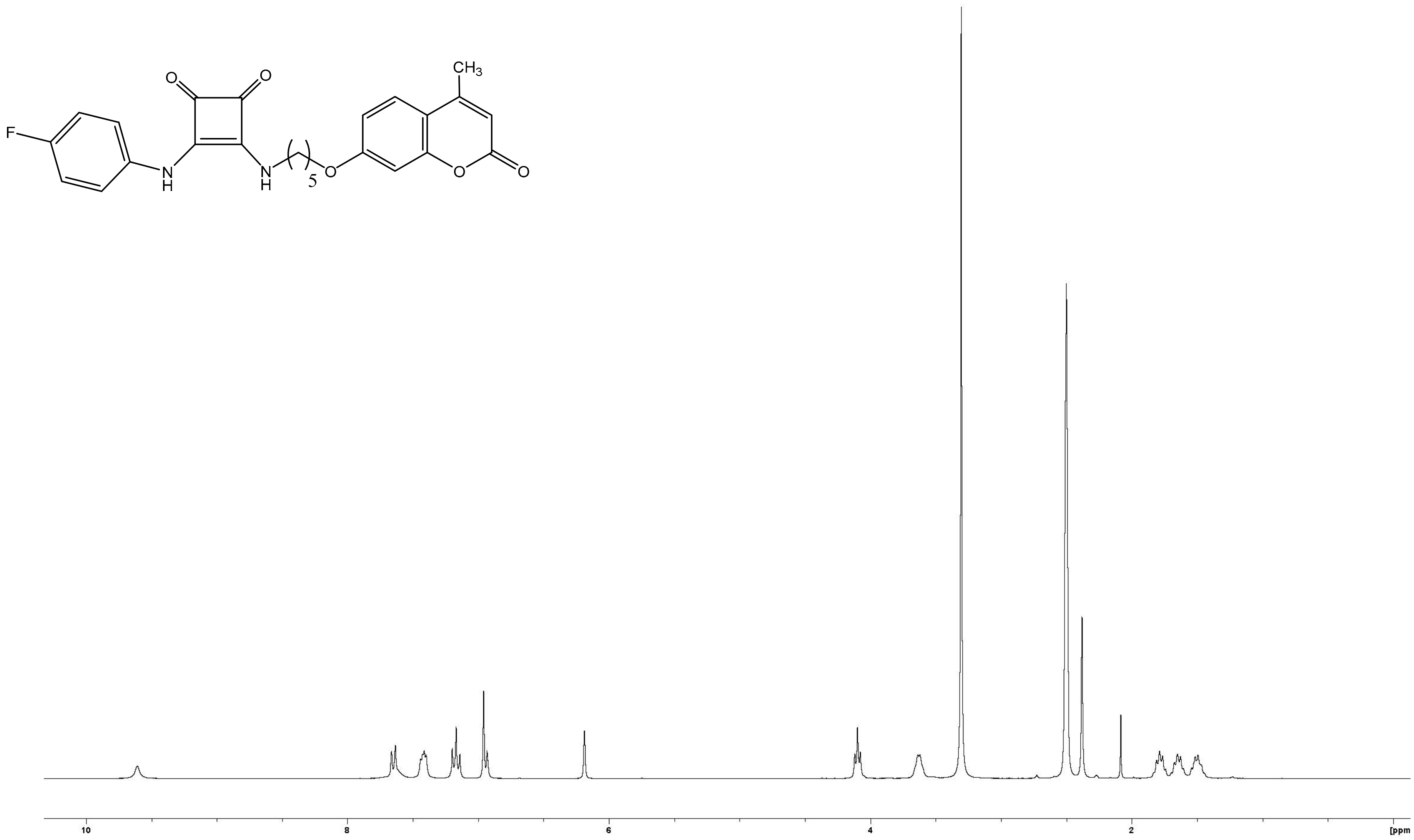

^{1}H-NMR (300 MHz, $(CD_3)_2SO$) of **16f**

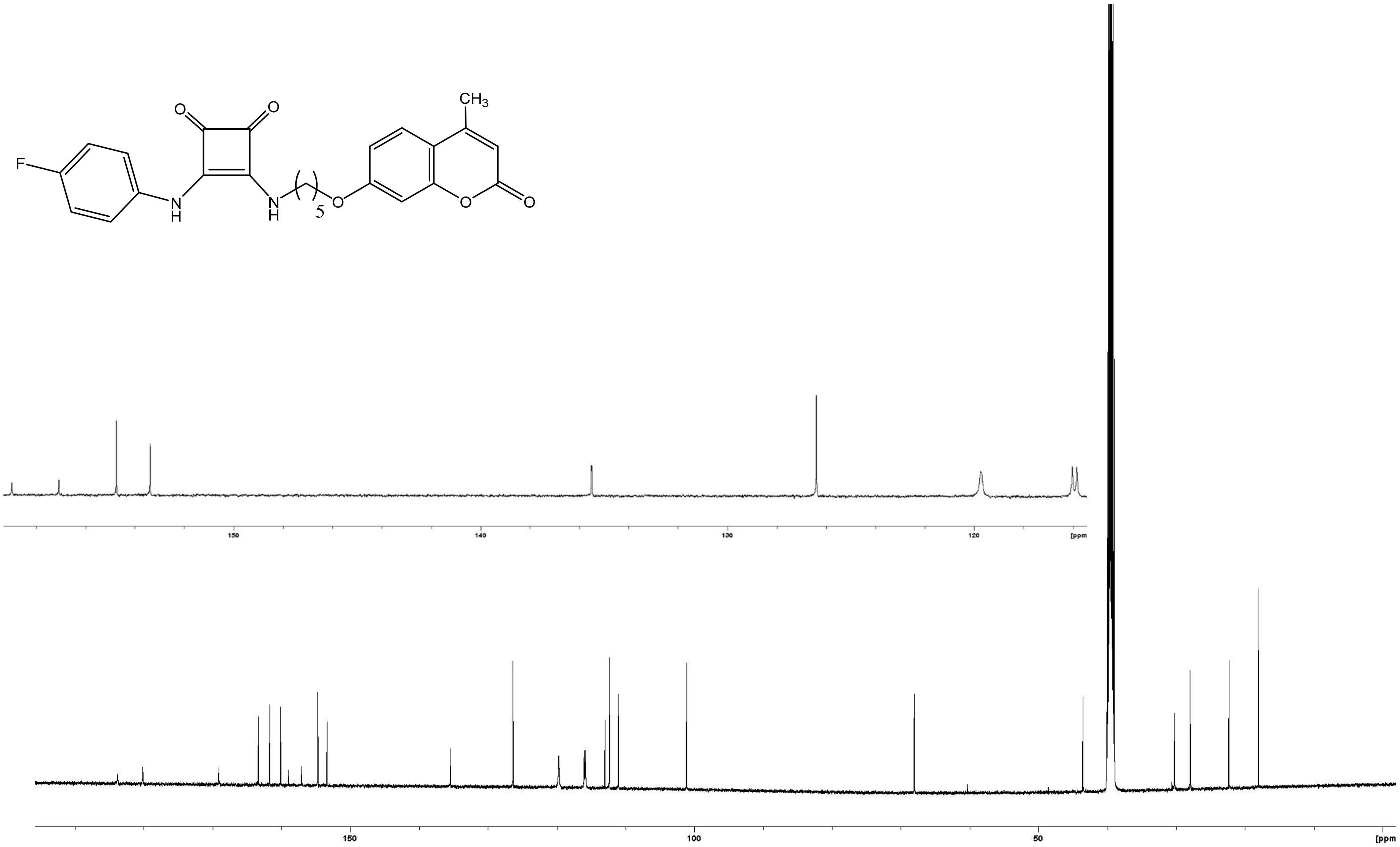

^{13}C-NMR (125.7 MHz, $(CD_3)_2SO$) of **16f**

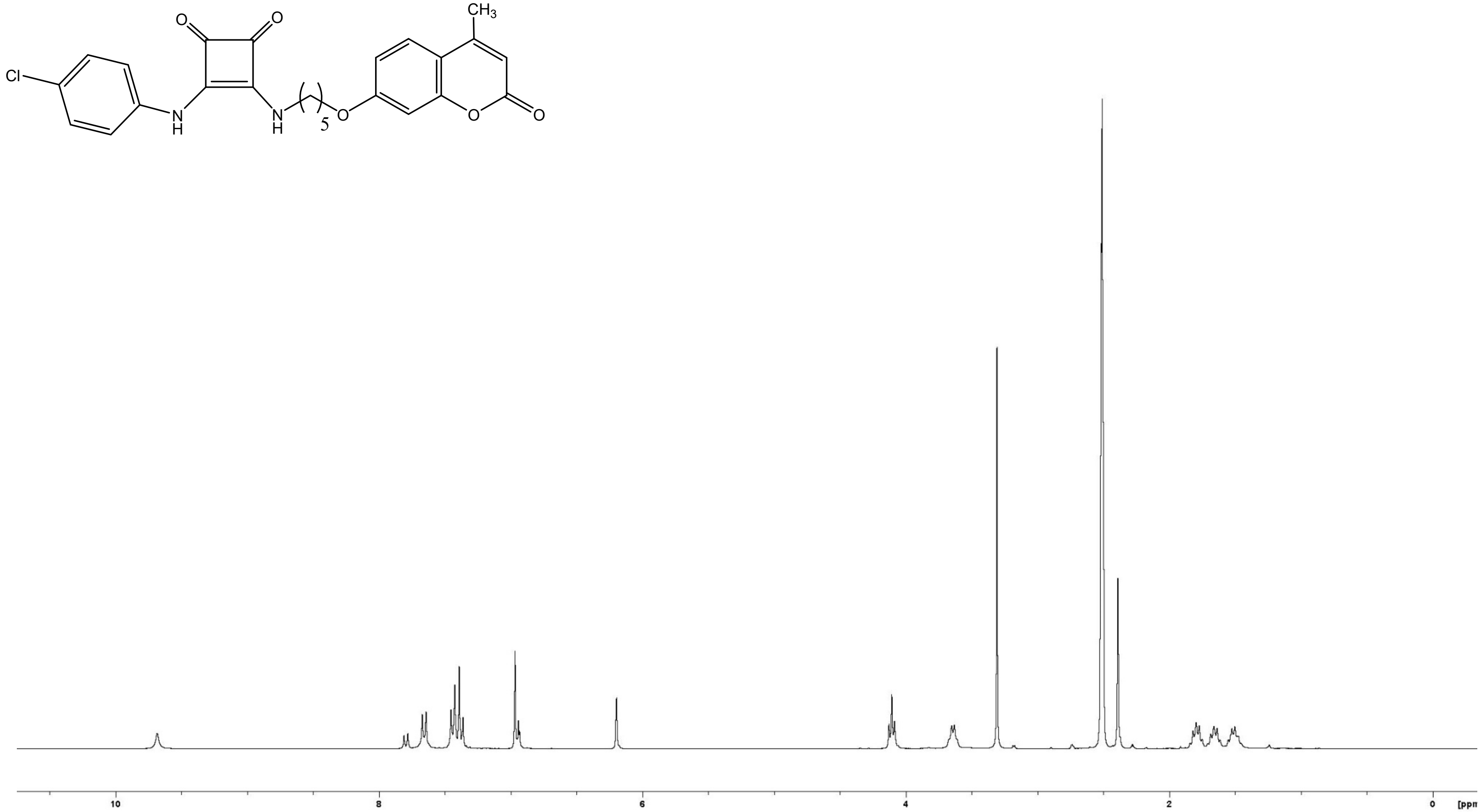

^{1}H-NMR (300 MHz, $(CD_3)_2SO$) of **16g**

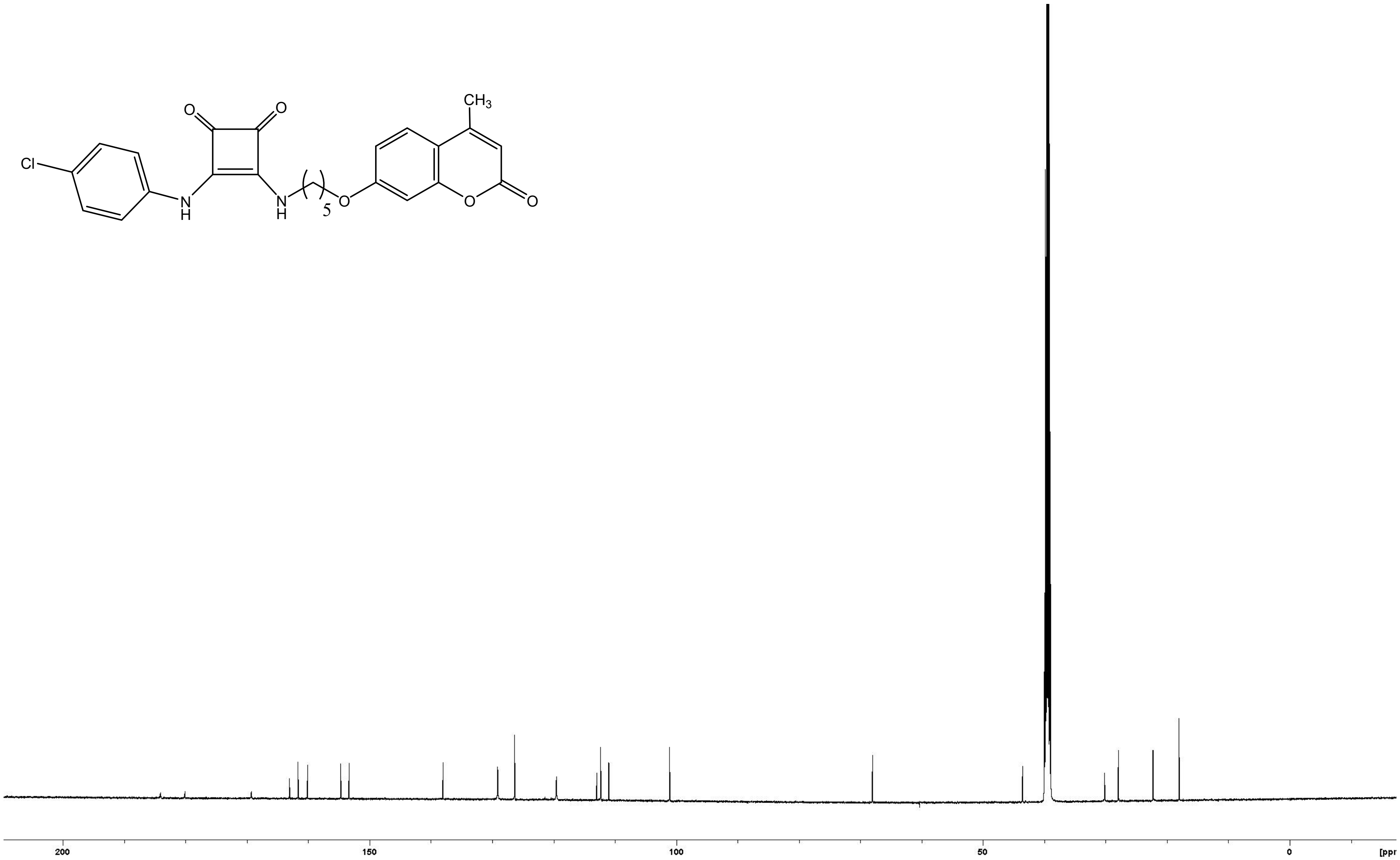

^{13}C-NMR (125.7 MHz, $(CD_3)_2SO$) of **16g**

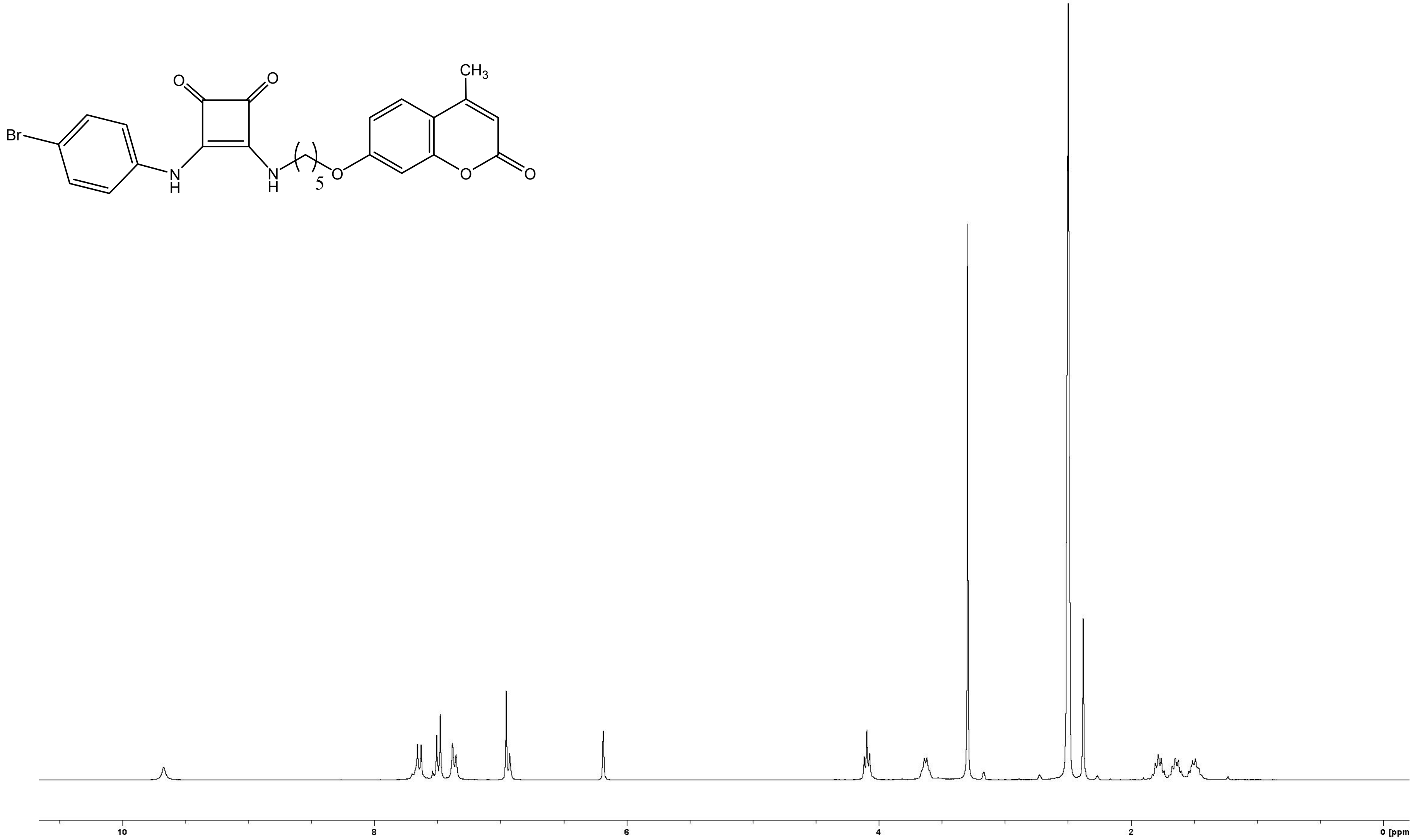

^{1}H-NMR (300 MHz, $(CD_3)_2SO$) of **16h**

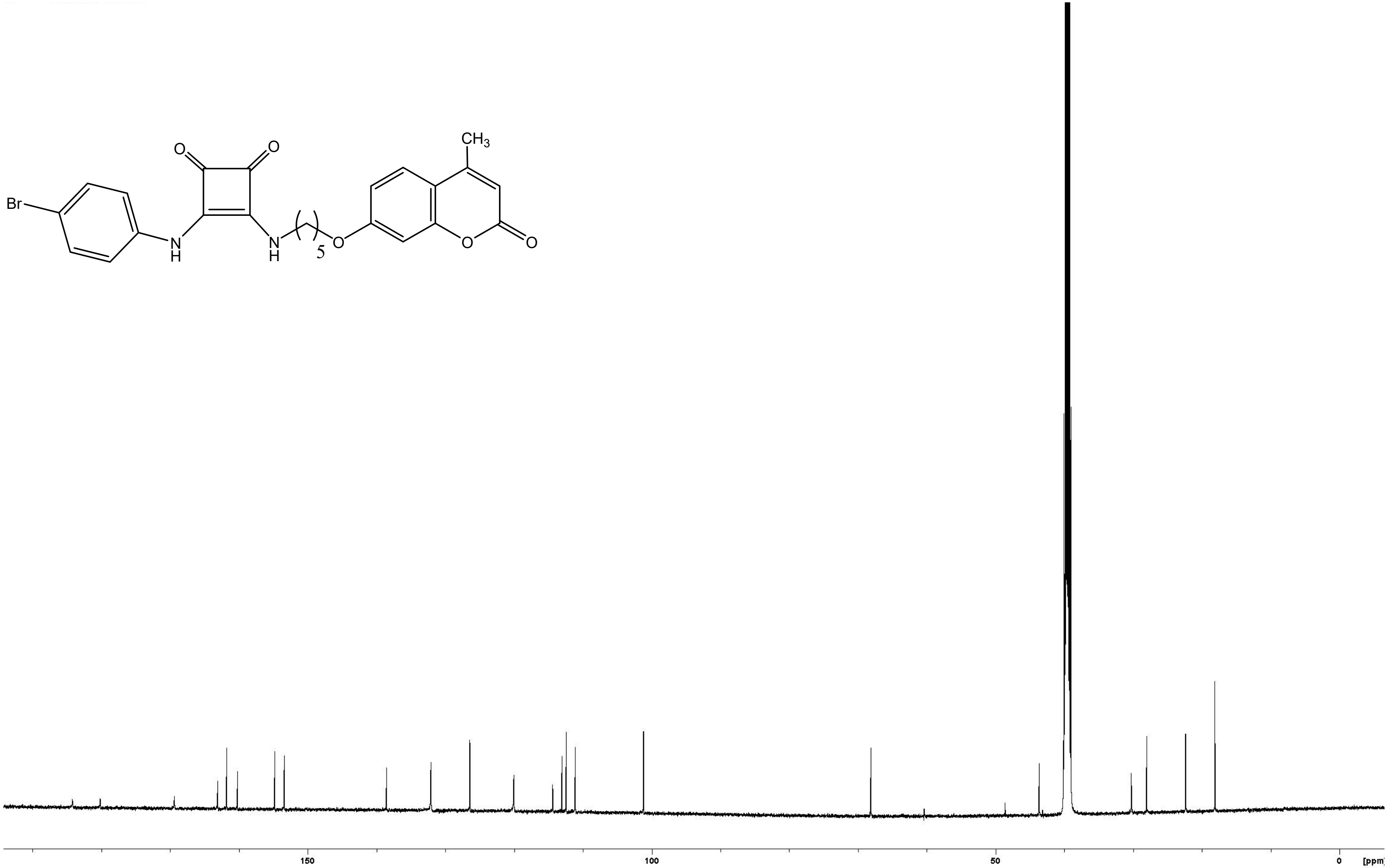

^{13}C-NMR (125.7 MHz, $(CD_3)_2SO$) of **16h**

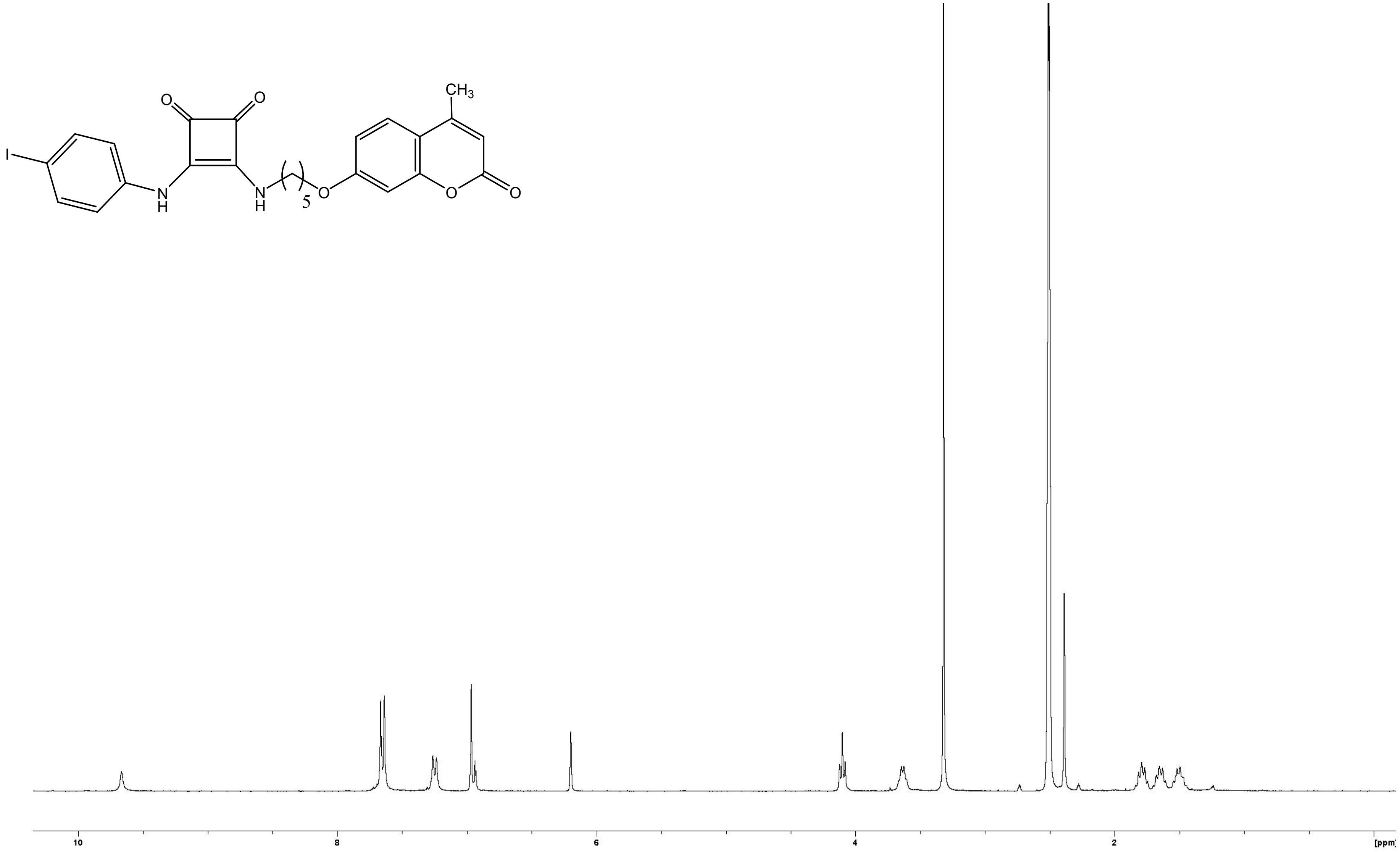

^{1}H-NMR (300 MHz, $(CD_3)_2SO$) of **16i**

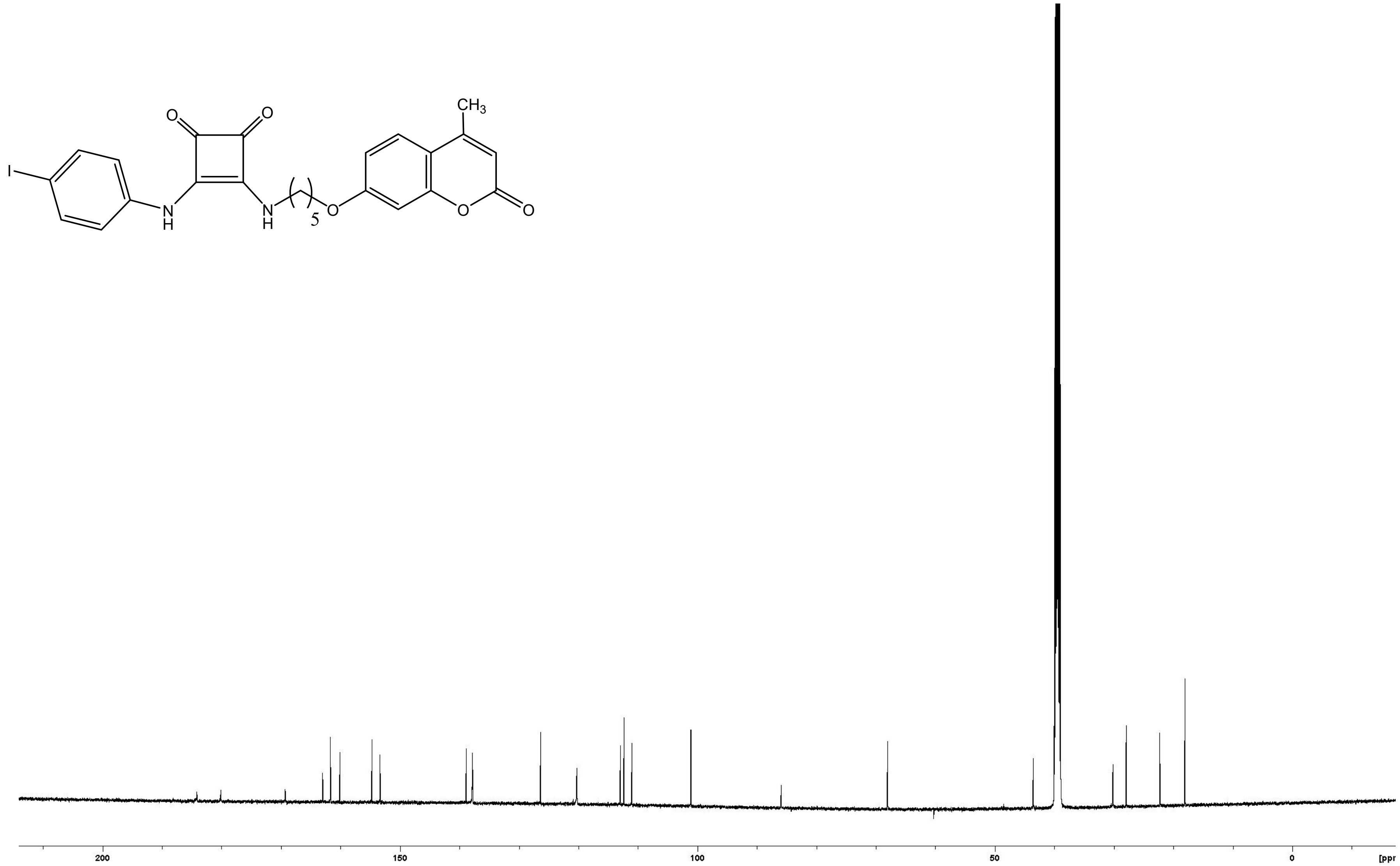

^{13}C-NMR (125.7 MHz, $(CD_3)_2SO$) of **16i**

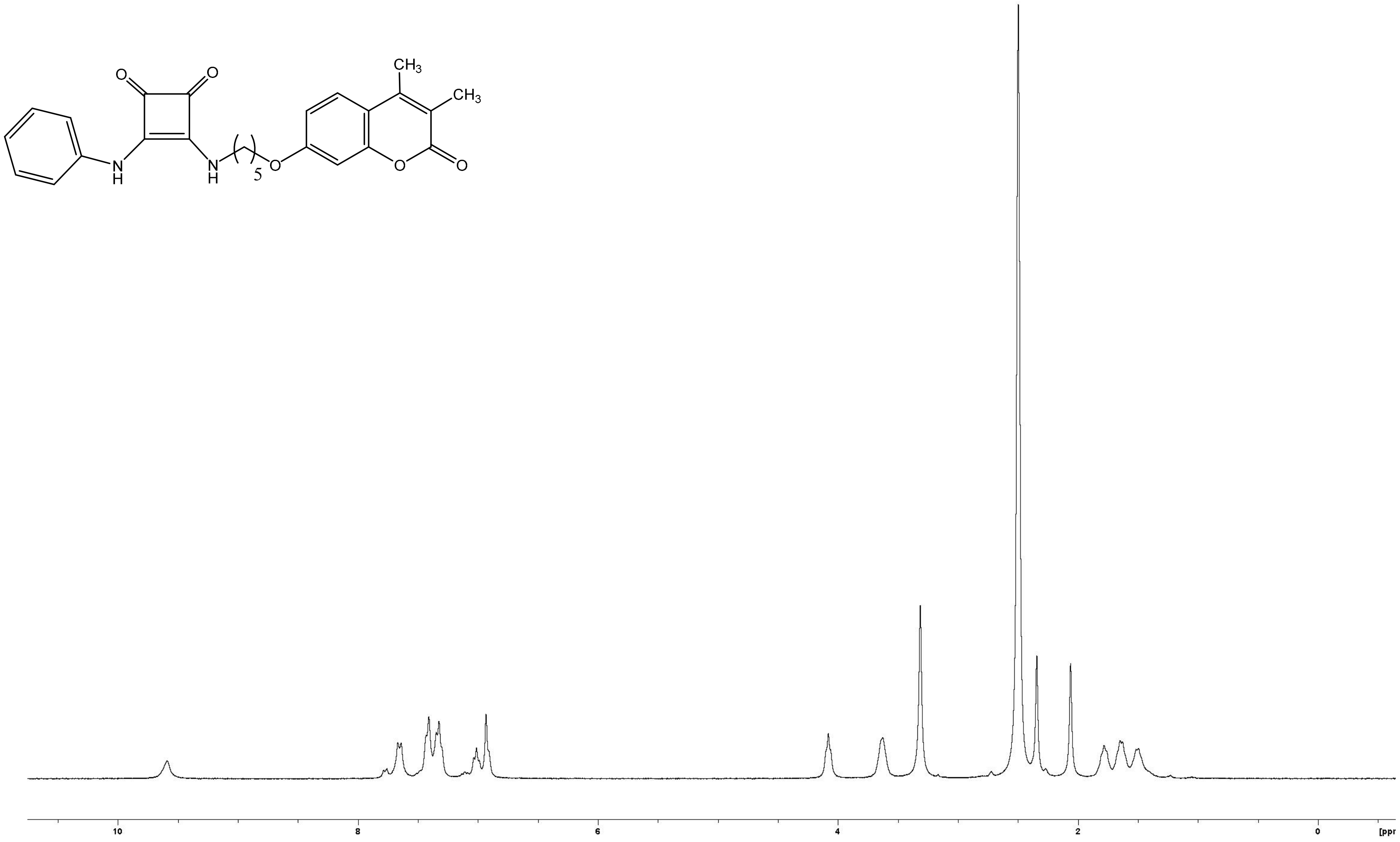

^{1}H-NMR (125.7 MHz, $(CD_3)_2SO$) of **16j**

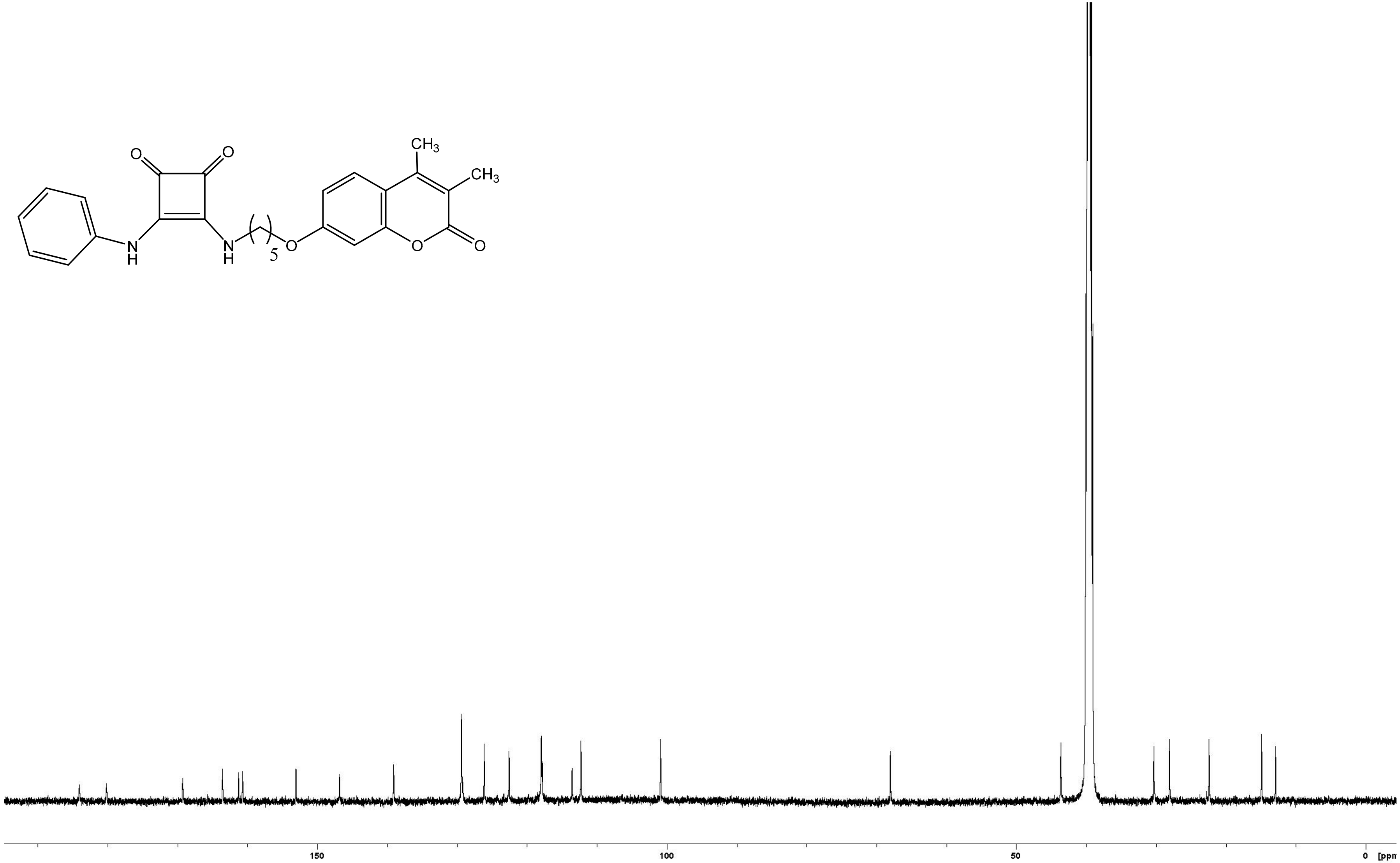

^{13}C-NMR (125.7 MHz, $(CD_3)_2SO$) of **16j**

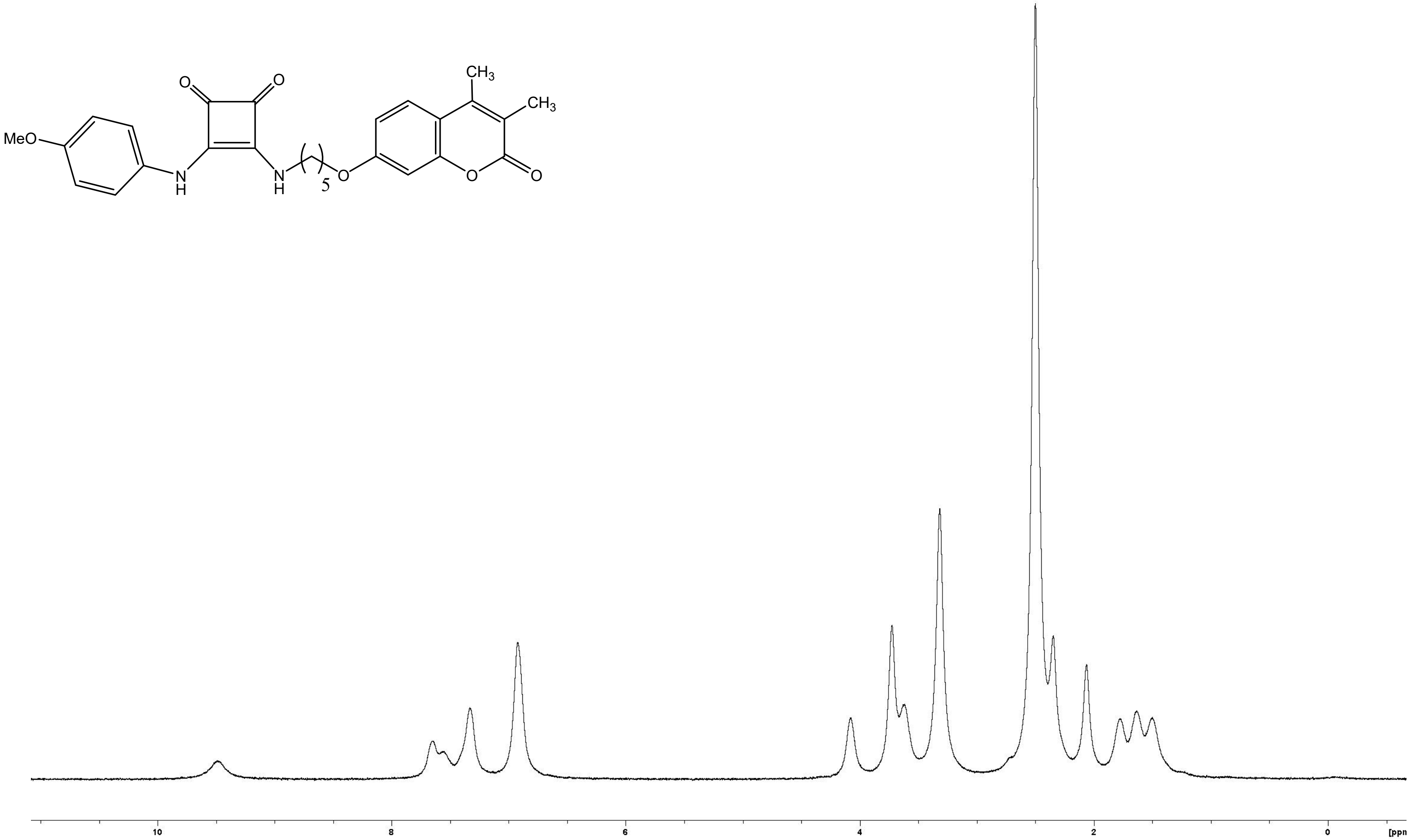

^{1}H-NMR (125.7 MHz, $(CD_3)_2SO$) of **16k**

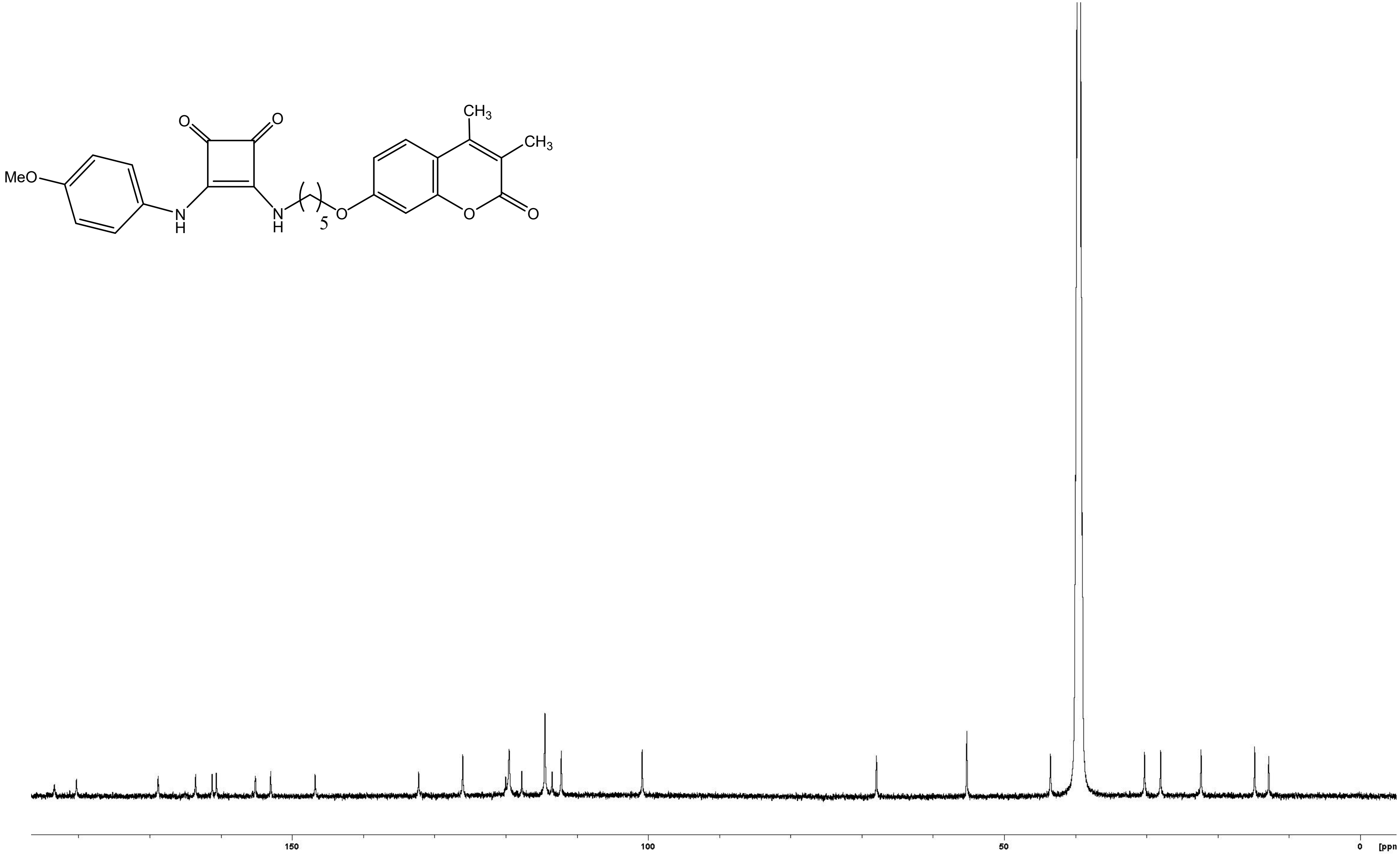

^{13}C-NMR (125.7 MHz, $(CD_3)_2SO$) of **16k**

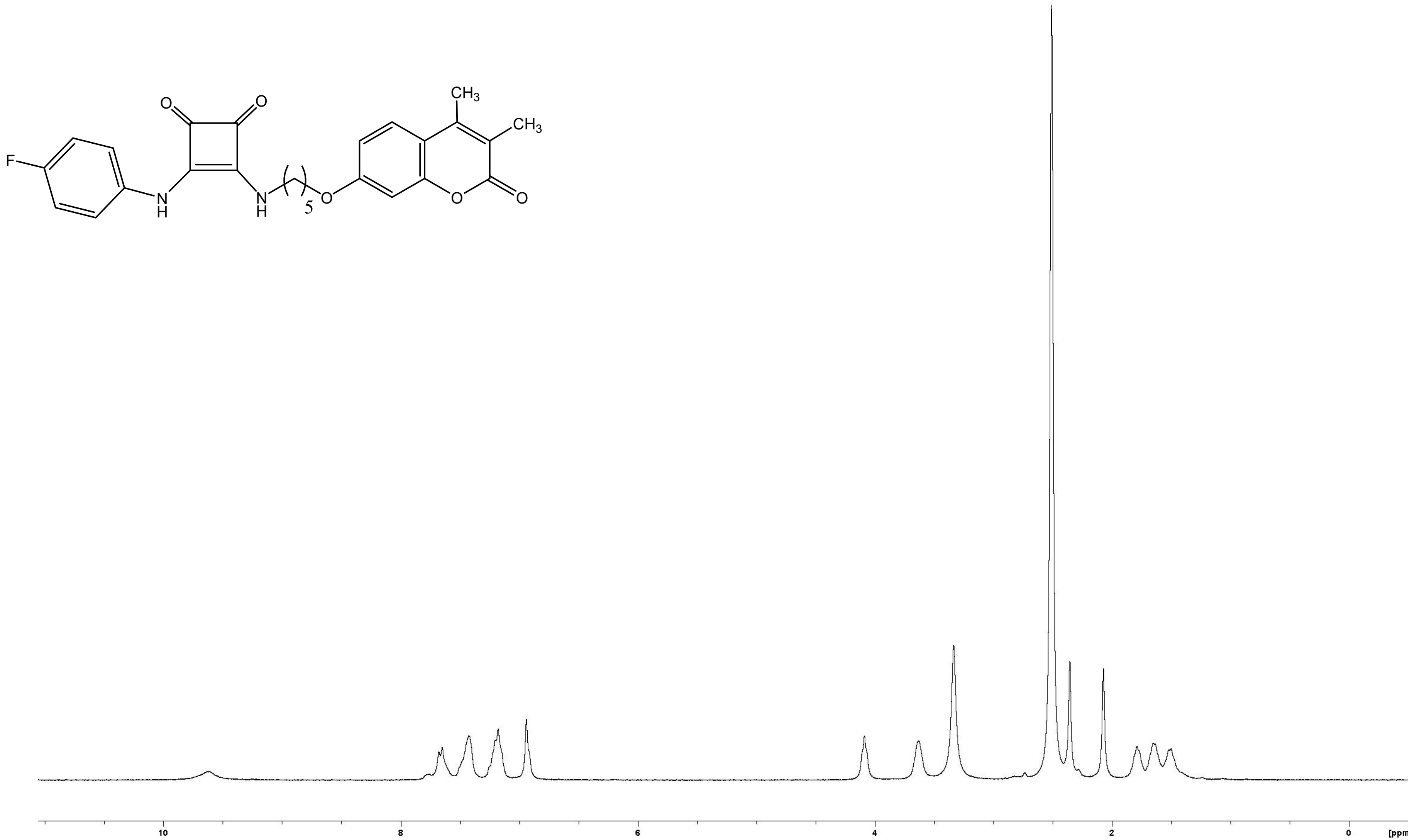

^{1}H-NMR (125.7 MHz, $(CD_3)_2SO$) of **16l**

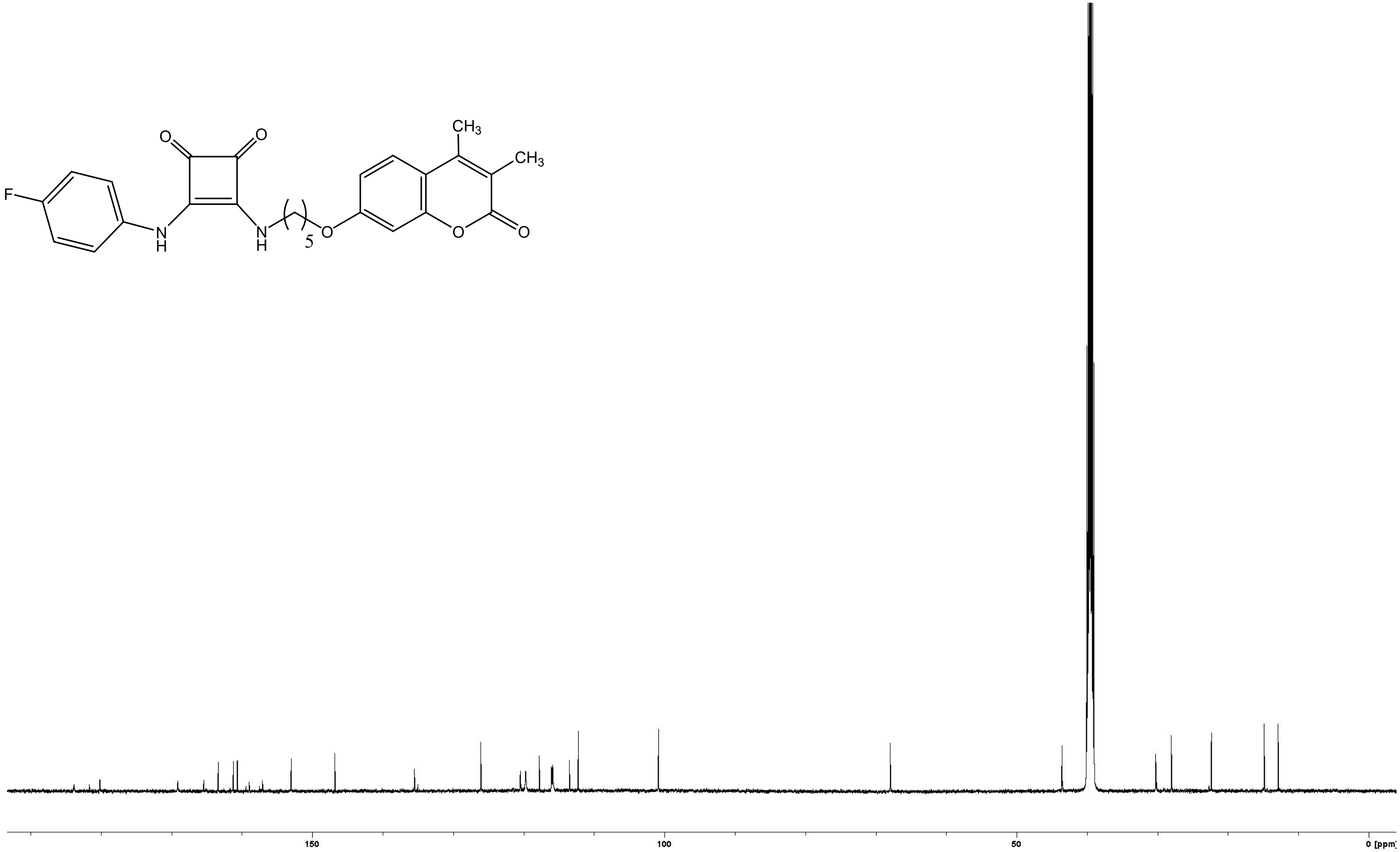

^{13}C-NMR (125.7 MHz, $(CD_3)_2SO$) of **16l**

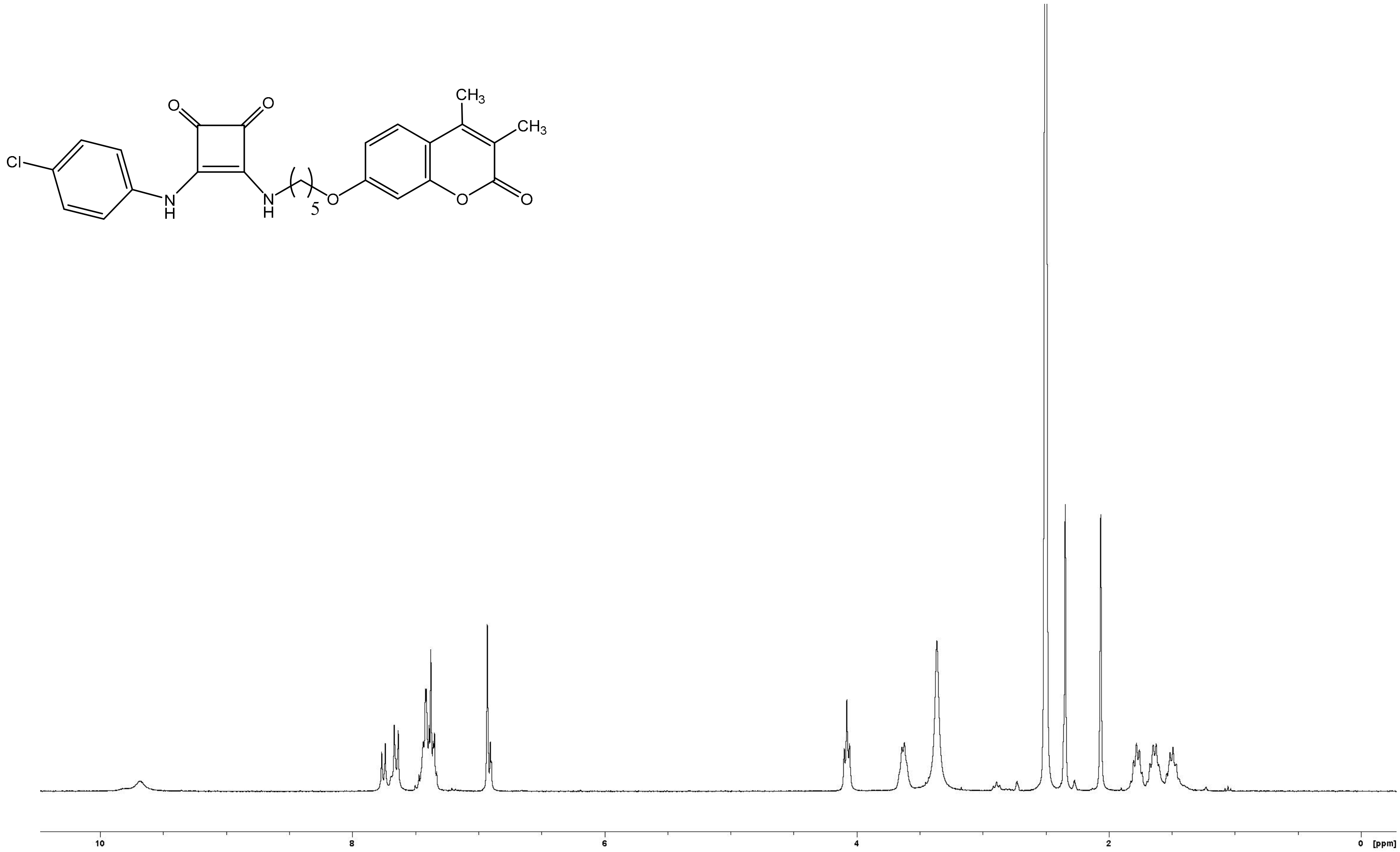

^{1}H-NMR (125.7 MHz, $(CD_3)_2SO$) of **16m**

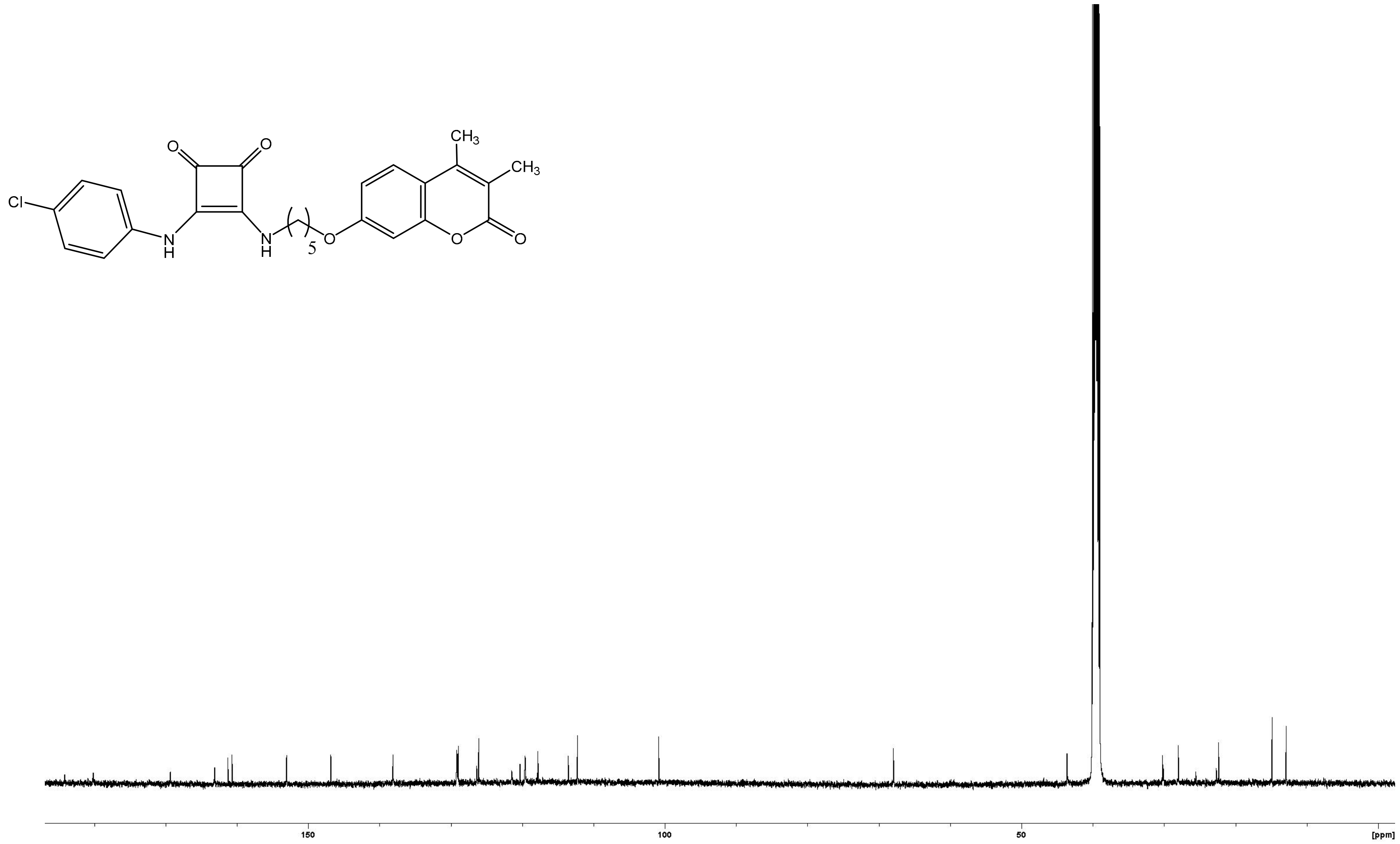

^{13}C-NMR (125.7 MHz, $(CD_3)_2SO$) of **16m**

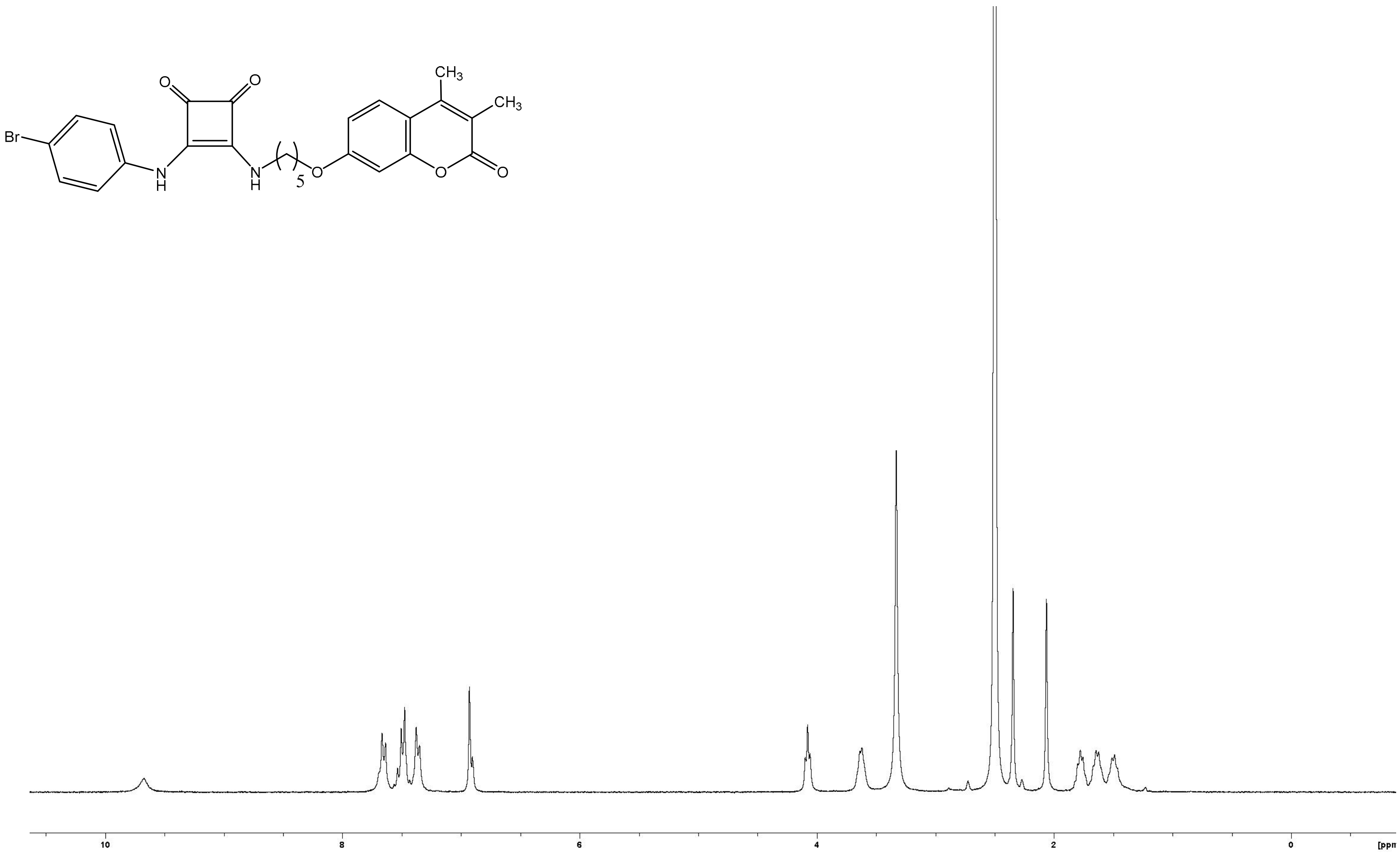

^{1}H-NMR (125.7 MHz, $(CD_3)_2SO$) of **16n**

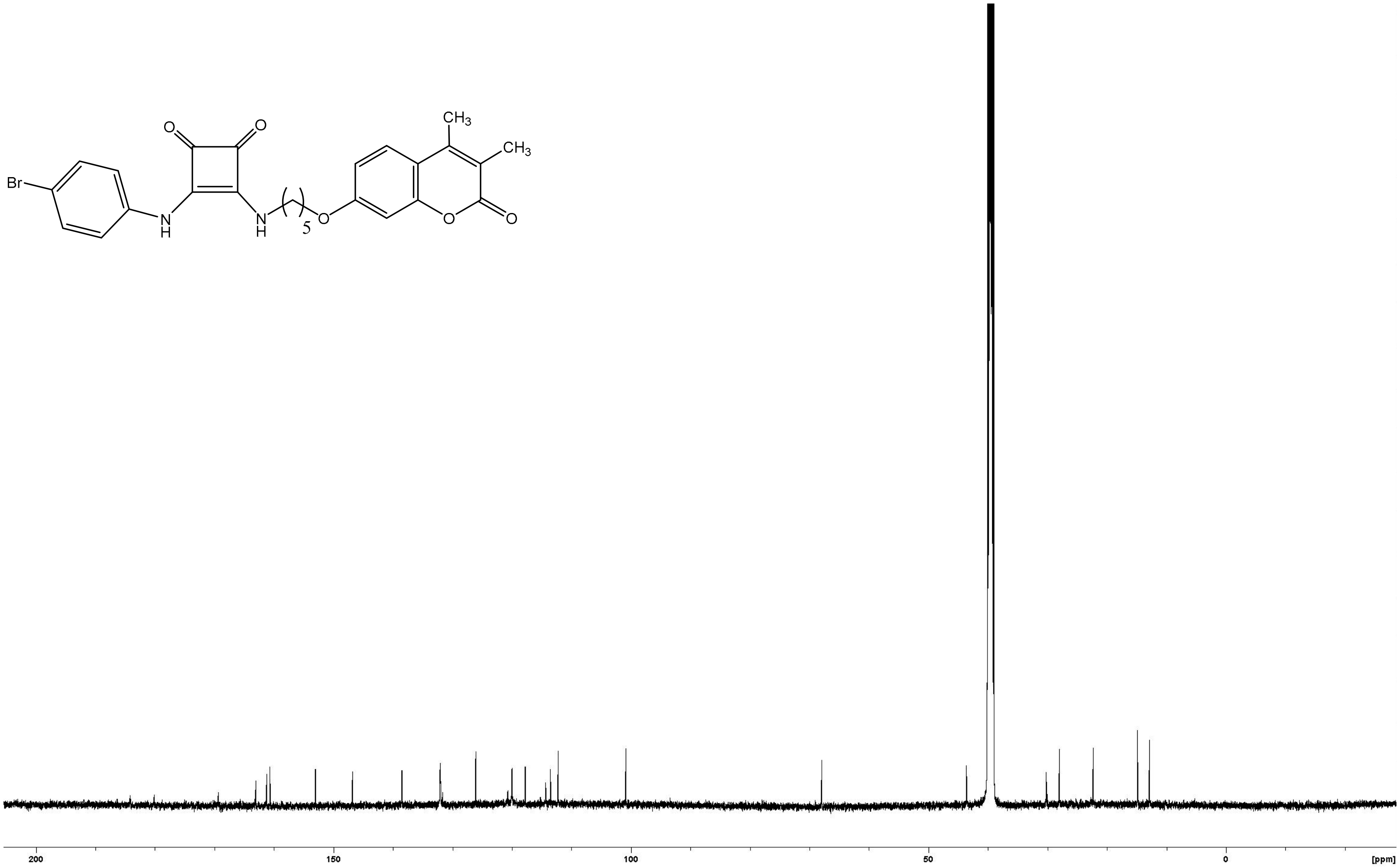

^{13}C-NMR (125.7 MHz, $(CD_3)_2SO$) of **16n**

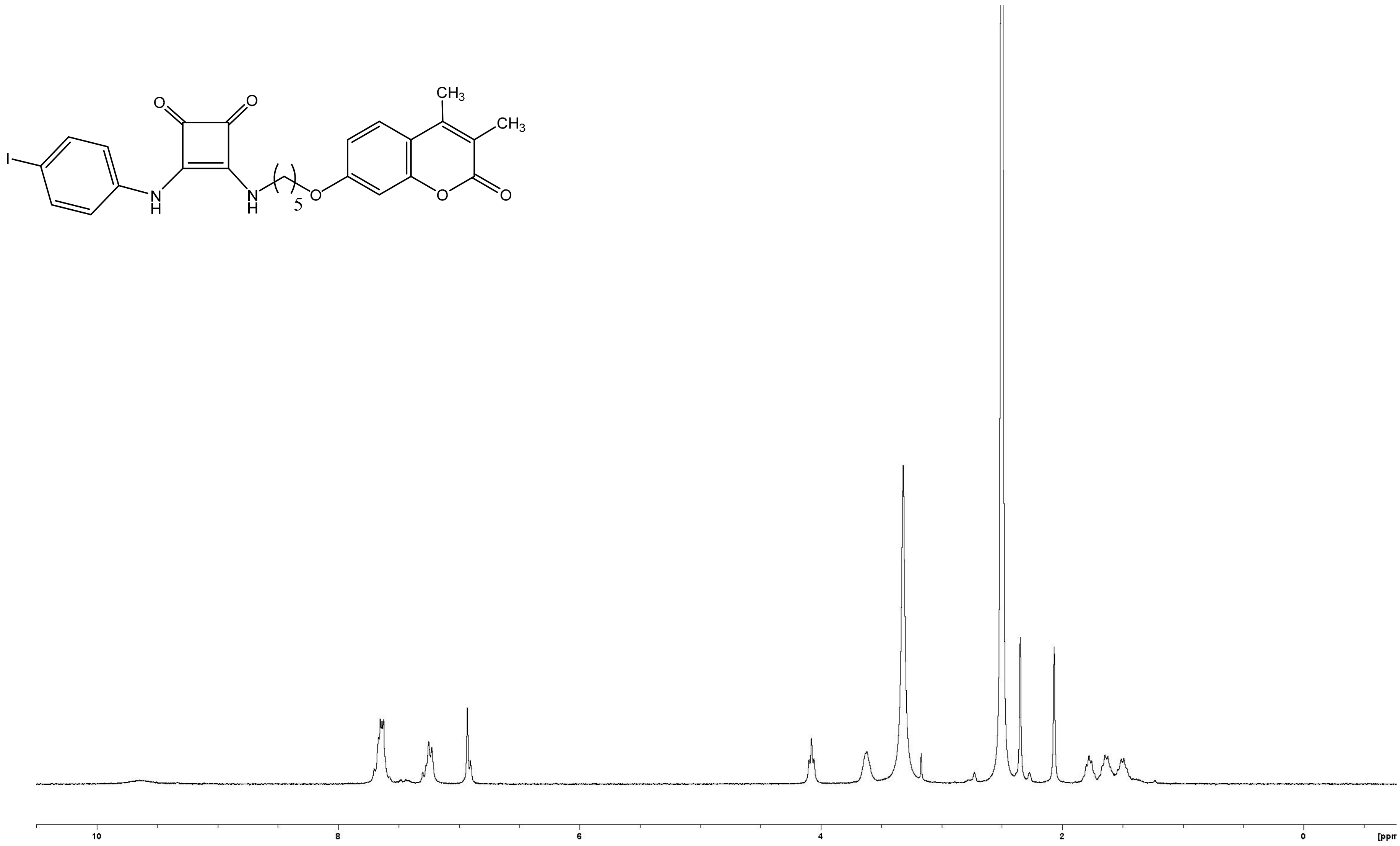

^{1}H-NMR (125.7 MHz, $(CD_3)_2SO$) of **16o**

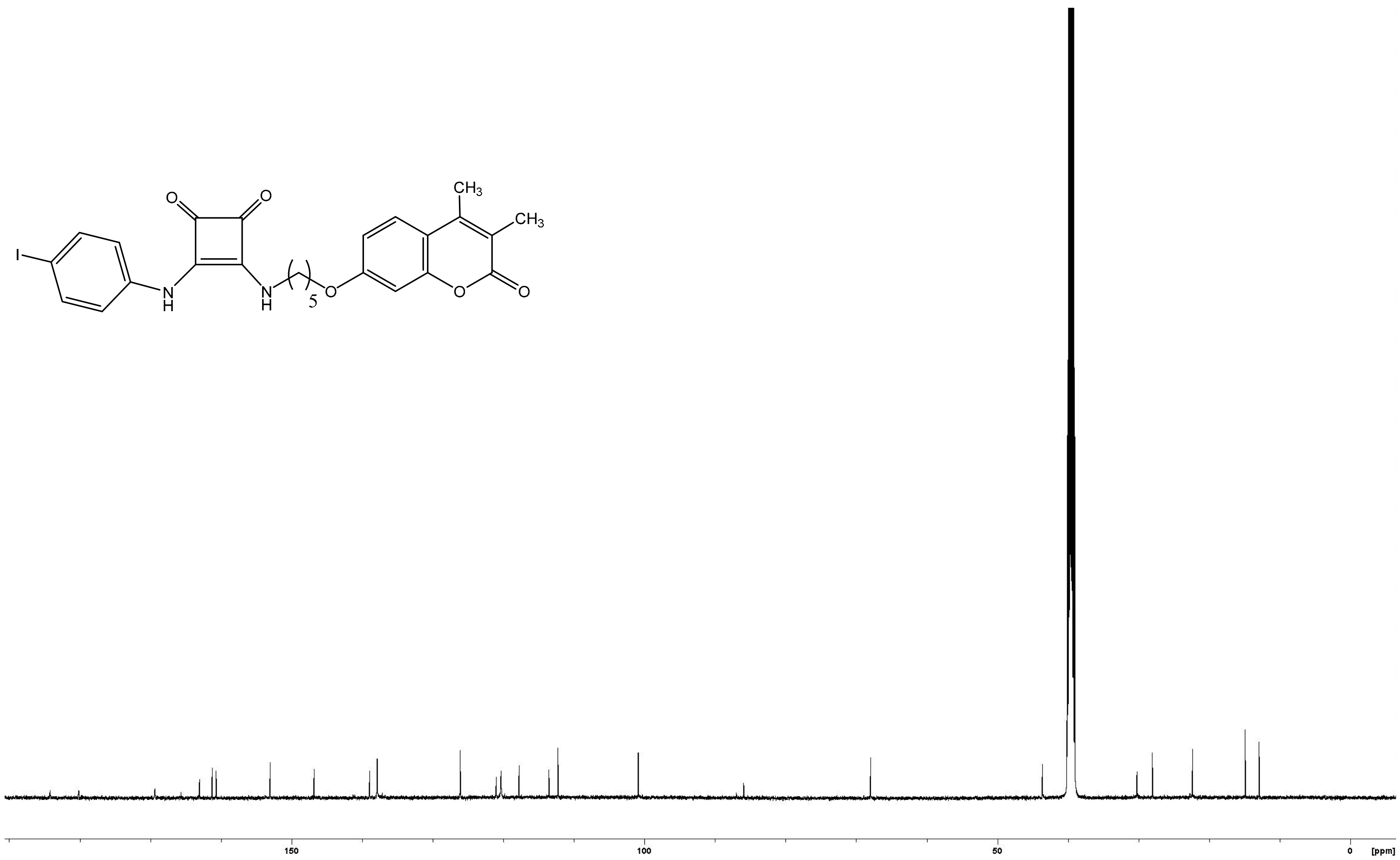

^{13}C-NMR (125.7 MHz, $(CD_3)_2SO$) of **16o**

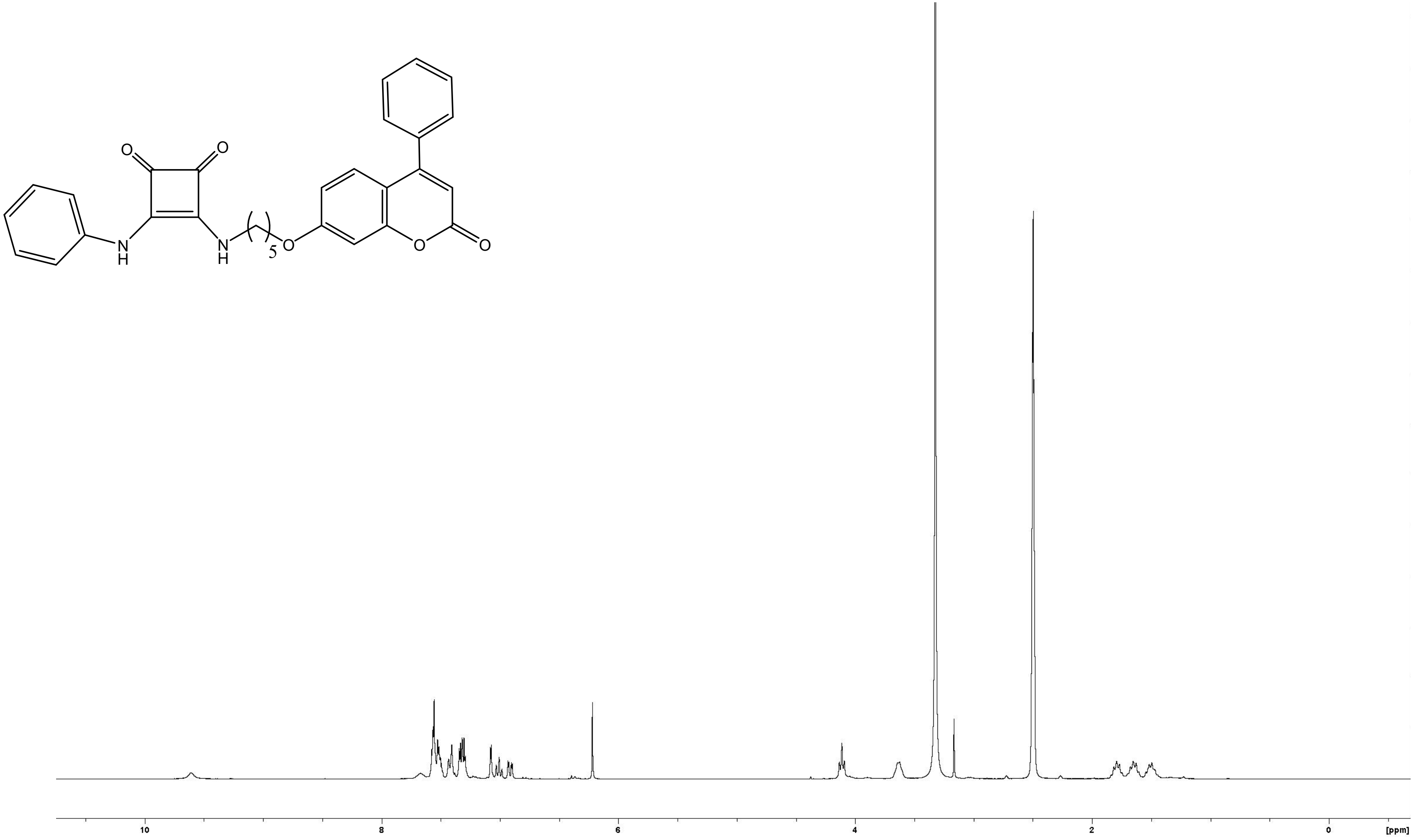

^{1}H-NMR (125.7 MHz, $(CD_3)_2SO$) of **16p**

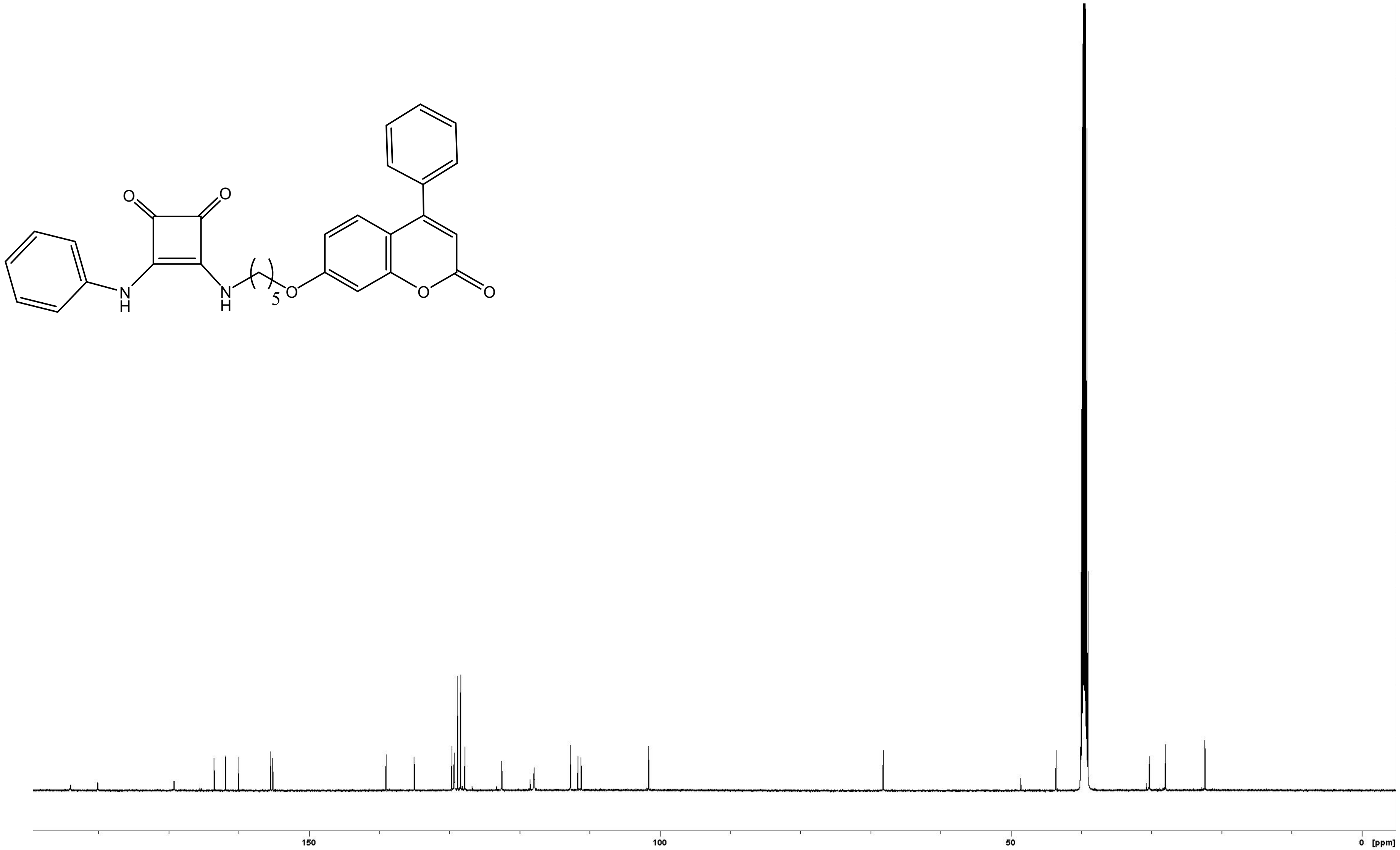

^{13}C-NMR (125.7 MHz, $(CD_3)_2SO$) of **16p**
